# A Liquid Chromatography with Tandem Mass Spectrometry-Based Proteomic Analysis of Primary Cultured Cells and Subcultured Cells Using Mouse Adipose-Derived Mesenchymal Stem Cells

**DOI:** 10.1155/2019/7274057

**Published:** 2019-01-10

**Authors:** Yoshiki Nakashima, Saifun Nahar, Chika Miyagi-Shiohira, Takao Kinjo, Naoya Kobayashi, Issei Saitoh, Masami Watanabe, Jiro Fujita, Hirofumi Noguchi

**Affiliations:** ^1^Department of Regenerative Medicine, Graduate School of Medicine, University of the Ryukyus, Okinawa 903-0215, Japan; ^2^Department of Infectious, Respiratory, and Digestive Medicine, University of the Ryukyus, Okinawa 903-0215, Japan; ^3^Department of Basic Laboratory Sciences, School of Health Sciences in Faculty of Medicine, University of the Ryukyus, Okinawa 903-0215, Japan; ^4^Okayama Saidaiji Hospital, Okayama 704-8192, Japan; ^5^Division of Pediatric Dentistry, Graduate School of Medical and Dental Science, Niigata University, Niigata 951-8514, Japan; ^6^Department of Urology, Okayama University Graduate School of Medicine, Dentistry and Pharmaceutical Sciences, Okayama 700-8558, Japan

## Abstract

Adipose-derived mesenchymal stem cells (MSC-ATs) are representative cell sources for cell therapy. However, how cell stress resulting from passage influences the MSC-AT protein expression has been unclear. In this study, a protein expression analysis was performed by liquid chromatography with tandem mass spectrometry (LC-MS/MS) using mouse primary cultured cells (P0) and cells passaged three times (P3) as samples. A total of 256 proteins were classified as cellular process-related proteins, while 179 were classified as metabolic process-related proteins in P0. These were considered to be adaptive responses of the cells to an in vitro environment. However, seven proteins of growth were identified (Csf1, App, Adam15, Alcam, Tbl1xr1, Ninj1, and Sbds) in P0. In addition, four proteins of antioxidant activity were also identified (Srxn1, Txndc17, Fam213b, and Apoe) in P0. We identified 1139 proteins expressed in both P0 and P3 cells that had their expression decreased to 69.4% in P3 cells compared with P0 cells, but 1139 proteins are very likely proteins that are derived from MSC-AT. The function of MSC-ATs was maintained after three passages. However, the LC-MS/MS analysis data showed that the protein expression was degraded after three passages. MSC-ATs retained about 70% of their protein expression ability in P3 cells.

## 1. Introduction

Mesenchymal stromal stem cells (MSCs) are considered to have the ability to differentiate into mesenchymal cells, such as osteoblasts, adipocytes, muscle cells, and chondrocytes [1, 2]. These cells are also expected to have an immunosuppressive effect and are regarded as promising cellular therapeutic agents for immunological diseases resistant to treatment. MSCs have been established from various tissues (umbilical cord blood, placenta, adipose tissue, etc.), among which adipose tissue contains a particularly large amount of cells.

Clinical research and treatment using adipose-derived mesenchymal stem cells (MSC-ATs) [3] is already underway in many medical institutions around the world [4]. The clinical practical application of islet cell transplantation therapy was reported in 2000 in the Edmonton protocol [5] and many subsequent papers [6-10]. The technology of islet transplantation is thought to be useful for the processing of therapeutic cells using MSC-ATs. We recently reported that the University of Wisconsin (UW) [11] organ preservation solution has a better cell survival/proliferation ability than Hank's balanced salt solution (HBSS) [12]. There is a possibility that the adipose tissue collected through the patient's skin may be infected with skin bacteria. One method of sterilizing tissues collected from a living body involves immersing and storing such tissue for 16 h using HBSS [13], which also contains antibiotics. After such storage, MSC-ATs can be isolated from adipose tissue. In addition, adipose tissue collected from a patient can be transported to a remote location.

It was recently reported that the stress of long-term culture of cells *in vitro* also occurs in stem cells, such as induced pluripotent stem (iPS) cells, causing DNA damage and cellular carcinogenesis [14]. Some researchers recommend reducing the number of passages of MSC-ATs to maintain the quality of primary cultured cells. However, MSC-ATs of primary cultured cells are reportedly contaminated with various types of cells, such as blood cells, through the process of cell isolation. This is because the stromal vascular fraction (SVF) [15] obtained when collecting MSC-ATs using centrifugation contains many kinds of cells (e.g., adipocytes, fibroblasts, smooth muscle cells, endothelial cells, blood cells, endothelial progenitor cells, preadipocytes, vascular progenitors, hematopoietic progenitors, and hematopoietic stem cells) [16, 17]. For MSC-ATs isolated from adipocytes collected from patients, the number of cells can be increased by increasing the number of passages. Because this processing can be done outside the body, the patient can thus obtain many of her/his own cells after undergoing only one procedure of fat collection surgery. However, it is also important to maintain the quality of the cells. Therefore, researchers and clinicians have fervently discussed how many passaging operations of clinical MSC-ATs should be performed.

With liquid chromatography (or high-performance liquid chromatography (HPLC)) with tandem mass spectrometry (LC-MS/MS), the components to be analyzed are separated by a liquid chromatograph (LC) and ionized via a dedicated interface (ion source) and the generated ions are then separated by MS. LC-MS/MS is an analytical technique that dissociates and fragments mass ions and detects them with MS [18]. Recently, an online LC-MS/MS system for quantitative proteomics based on data-dependent protein IDs and shotgun-based quantitative proteomics methods was developed [19-23] by connecting the measuring equipment for a protein analysis to a computer and linking to an online protein database. In this way, a comprehensive protein expression analysis can be performed by checking the peptide sequence data of the protein contained in the sample.

A comprehensive expression analysis of the protein expressed by the cell is important for accurately understanding the mechanism underlying the effect of cell therapy accompanying the administration of the culture supernatant of cells and the cells themselves. The present study was performed to identify the functional protein components in mouse MSC-ATs of primary cultured cells (P0) and mMSC-ATs passaged three times (P3) using LC-MS/MS. The proteins specifically contained in primary cultured cells were identified as those expressed only in the SVF derived from AT. By examining the proteins expressed in both P0 and P3 cells, we can identify the proteins expressed by MSC-ATs. Determining the protein component of MSC-ATs that exerts a therapeutic effect is expected to be useful for cell therapy in the future.

## 2. Materials and Methods

### 2.1. Reagents

Fetal bovine serum (FBS) was obtained from Biowest (Nuaille, France). DMEM (high glucose) with L-glutamine, phenol red, and sodium pyruvate was obtained from FUJIFILM Wako Pure Chemical Corporation (Osaka, Japan). Plastic dishes were obtained from TPP (Trasadingen, Switzerland). All other materials used were of the highest commercial grade.

### 2.2. Animal Care

All experimental protocols were in accordance with the guidelines for the care and use of laboratory animals set by Research Laboratory Center, Faculty of Medicine, and the Institute for Animal Experiments, Faculty of Medicine, University of the Ryukyus (Okinawa, Japan). The experimental protocol was approved by the Committee on Animal Experiments of University of the Ryukyus (permit number: A2017101). C57BL/6 male mice (8 weeks of age; Japan SLC, Shizuoka, Japan) were maintained under controlled temperature (23 ± 2°C) and light conditions (lights on from 08:30 to 20:30). Animals were fed standard rodent chow pellets with *ad libitum* access to water. All efforts were made to minimize the suffering of the animals.

### 2.3. Isolation of MSC-ATs from Mouse via the Inguinal Pad Fat

AT was obtained from the inguinal pad fat of three 8-week-old mice. The method of isolating MSC-ATs from AT was in accordance with the AT-derived stem cell product standard document (RMRC-A 01: 2015) of Ryukyus Regenerative Medicine Research Center. In brief, these ATs were stored in cold HBSS and washed vigorously using HBSS three times before starting digestion. Next, the tissues were cut into small fragments with a scalpel for enzymatic digestion (2 mg collagenase type IV/ml; HBSS) in 50 ml tubes (rotation speed: 20 × 37°C × 60 min) using the shaker (BioShaker BR-42FM; TAITEC, Saitama, Japan). These tubes were then centrifuged (800*g*) for 5 minutes. The SVF [24] containing various kinds of cells, including MSC-ATs, was confirmed at the bottom of the tube after centrifugation. The MSC-ATS were collected as a cell pellet and then washed with fresh DMEM containing 10% FBS to remove the enzyme after the digestion. The digested tissue was then incubated in a T25 flask.

All of the mouse studies were approved by the Institutional Animal Care and Use Committee of University of the Ryukyus.

### 2.4. Preparation of mMSC-ATs

Mouse MSC-ATs were cultured (37°C, 5% CO_2_) in an uncoated T25 flask (TPP 90026). The passage of cells was performed every 3 to 4 days after reaching 80% confluence following sowing. The cells were then washed with PBS (calcium, magnesium-free), and mouse MSC-ATs were dissociated using a dissociation solution (Trypsin/EDTA (Lonza CC-3232)). Subculturing was carried out by plating on an uncoated T25 flask. DMEM containing 10% FBS was used for the culture medium.

### 2.5. Flow Cytometry

Cell flow cytometry was performed as described previously [25], using specific antibodies for CD34, CD 44, CD45, and CD90.2.

### 2.6. Cell Differentiation

Adipogenic and osteogenic differentiations were performed as described previously [25].

### 2.7. Protein Identification by a Nano-LC-MS/MS Analysis

We used an EzRIPA Lysis kit (ATTO Corporation, Tokyo, Japan) for cell lysis according to the manufacturer's instructions. A protein solution of 4493 *μ*g/ml (P0) and 3105 *μ*g/ml (P3) was obtained from mADSCs, and 6.0 *μ*g protein was used for sample preparation. Finally, 0.4 *μ*g of protein was used for nano-LC-MS/MS. The comprehensive expression analysis of proteins using LC-MS/MS and data analyses were performed according to the method reported previously [25].

## 3. Results and Discussion

The application of cell therapy in regenerative medicine is expected to be useful for the treatment of many kinds of diseases. For example, MSC-ATs, which can be collected from AT, have been applied to the treatment of a wide range of diseases in light of the low invasiveness compared with surgery. It is generally recognized that MSC-ATs are stable in quality from P0 cells to P5 cells [26]. As such, many manuals of commercially available MSC-ATs state that the quality is guaranteed for five passages. It was reported that multiple passaging processes reduce both the cell proliferative activity and the cell surface marker expression [27] and induce chromosome abnormalities [15]. In addition, increasing the number of passages also increases the risk of microbial infection of cells. Therefore, the general perception among clinical researchers dealing with therapeutic cells is that MSC-ATs are only useful as therapeutic cells within the first five passages [28].

However, it is easy to imagine that preparing cells collected from a living organism without subculturing may enable the production of therapeutic cells with new and special functions. Therefore, clinicians may try using MSC-ATs isolated from AT without any culturing or P0 MSC-ATs. Close attention must be paid in such cases, as immune rejection can be caused when using therapeutic cells not only for autologous transplantation but also other transplantations as well.

### 3.1. The Characteristics and Cell Quality of mMSC-ATs (P0)

**Figure 1:**
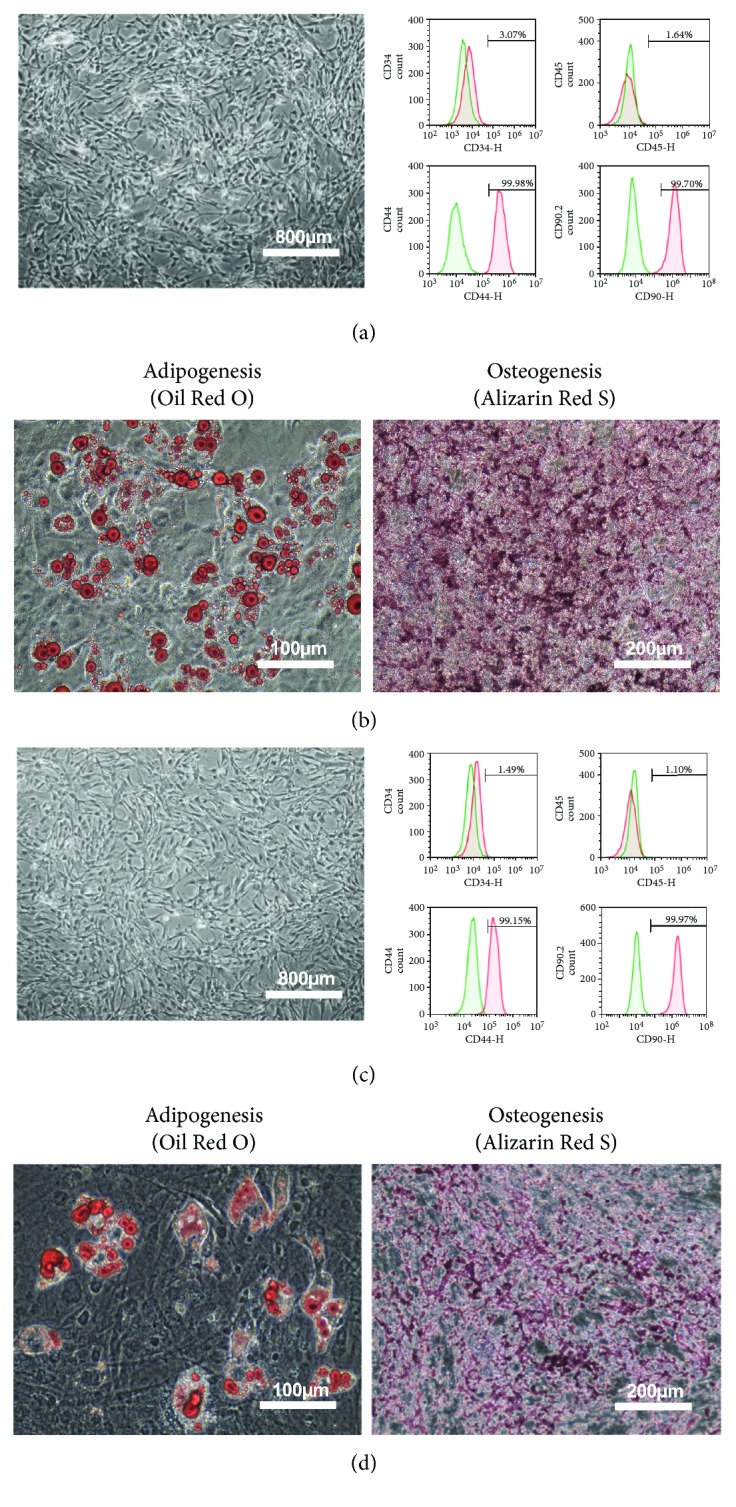
The phenotype and differentiation potential of mMSC-ATs in culture. The morphological appearance of mMSC-ATs (P0) ((a) left panel; scale bar = 800 *μ*m). The results of flow cytometry of the cell surface markers of mMSC-ATs (P0) ((a) right panels). Representative images of adipocyte ((b) left panel; scale bar = 100 *μ*m) and osteocyte differentiation ((b) right panel; scale bar = 200 *μ*m) of mouse MSC-ATs (P0) cultured in differentiation medium. The morphological appearance of mMSC-ATs (P3) ((c) left panel; scale bar = 800 *μ*m). The results of flow cytometry of the cell surface markers of mMSC-ATs (P3) ((c) right panels). Representative images of adipocyte ((d) left panel; scale bar = 100 *μ*m) and osteocyte differentiation ((d) right panel; scale bar = 200 *μ*m) of mouse MSC-ATs (P3) cultured in differentiation medium.

mMSC-ATs were cultured to 80% confluence using DMEM containing 10% FBS after isolation from AT. The whole medium was exchanged every two days. Microscopy was performed to confirm the absence of abnormalities with regard to the mMSC-AT (P0) size and shape and the culture state (Figure 1(a), left panel). Flow cytometry was performed using markers of mMSC-ATs (CD44, CD90.2), hematopoietic stem cells (CD34), and leukocytes (CD45). CD44 and CD90.2 were expressed in mMSC-ATs, while CD34 and CD45 were not detected (Figure 1(a), right panels). We induced differentiation into adipocytes (Figure 1(b), left panel) and osteoblasts (Figure 1(b), right panel) using mMSC-ATs. Mature adipocytes were stained with Oil Red O, and mature osteoblasts were stained with Alizarin Red S.

### 3.2. The Characteristics and Cell Quality of mMSC-ATs (P3)

mMSC-ATs were cultured to 80% confluence using DMEM containing 10% FBS after isolation from adipose tissue. The whole medium was exchanged every two days. The passage of cells was performed every 3 to 4 days after reaching 80% confluence. Microscopy was performed to confirm the absence of abnormalities with regard to the mMSC-AT (P3) size and shape and the culture state (Figure 1(c), left panel). Flow cytometry was performed using markers of mMSC-ATs (CD44, CD90.2), hematopoietic stem cells (CD34) and leukocytes (CD45). CD44 and CD90.2 were expressed in mMSC-ATs while CD34 and CD45 were not detected (Figure 1(c), right panels). We induced differentiation into adipocytes (Figure 1(d), left panel) and osteoblasts (Figure 1(d), right panel) using mMSC-ATs. Mature adipocytes were stained with Oil Red O, and mature osteoblasts were stained with Alizarin Red S.

A proteome analysis using LC-MS/MS provided evidence supporting the safe application of cell therapy with MSCs and supplied information on the potential application of MSCs in various treatments. A protein analysis indicates the protein components present in the cell component. In this study, mMSC-ATs were used, but when human MSC-ATs are used, this analysis will show the protein components that should be administered to patients.

### 3.3. A Comprehensive Protein Expression Analysis of mMSC-ATs (P0 and P3)

We performed mMSC-AT isolation according to a protocol similar to that used in clinical studies at the University of the Ryukyus, and isolated mMSC-ATs were subjected to LC-MS/MS after 0 or 3 passages. The presence of a large amount of albumin in the medium reduces the accuracy in protein analyses. Therefore, the protein extracts obtained from the cells after washing with phosphate-buffered saline (PBS) were used in this study.

**Figure 2:**
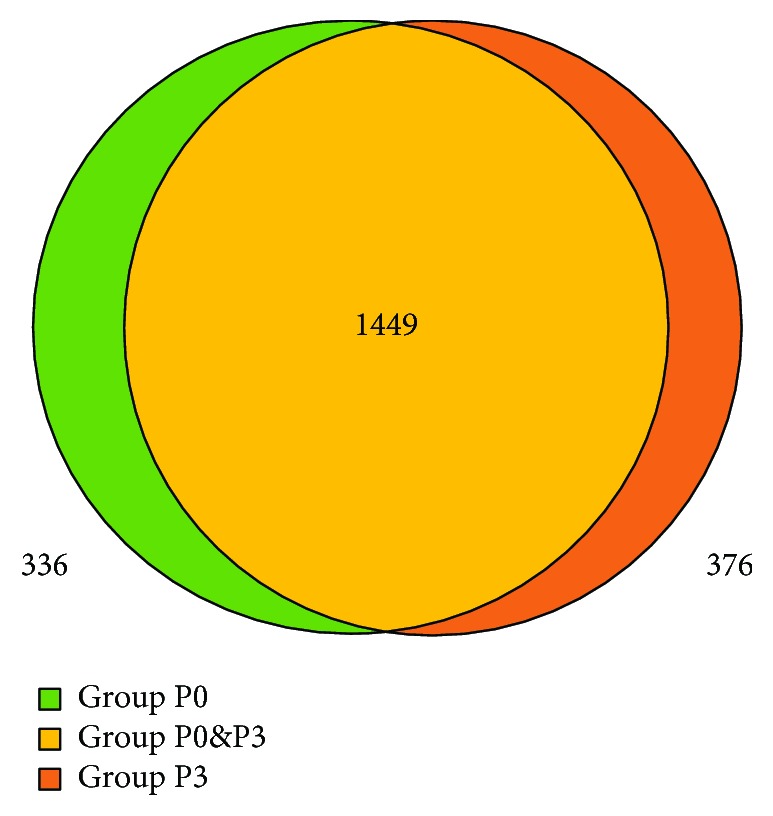
Venn diagram of proteins detected on LC-MS/MS. There were 1785 types of proteins identified from the mouse primary cultured cell (P0) sample and 1825 types of proteins identified from samples of cells passaged 3 times (P3). Among the 1785 types of proteins in mouse P0 cells, there were 336 types of proteins unique to the primary cultured cells (group P0). A total of 1449 types of proteins in mouse P0 cells were also identified in mouse P3 cells (group P0&P3). Among the 1825 types of proteins in mouse P3 cells, there were 376 types of proteins unique to the cells passaged 3 times (group P3).

There were 1785 types of proteins identified from the mMSC-AT (P0) samples (Table 1) and 1825 types of proteins identified from the mMSC-AT (P3) samples (Table 2). Among the 1785 types of proteins in mouse P0 cells, there were 336 types of proteins unique to the primary cultured cells (group P0). A total of 1449 types of proteins in mouse P0 cells were also identified in mouse P3 cells (group P0&P3). Among the 1825 types of proteins in mouse P3 cells, there were 376 types of proteins unique to the cells passaged 3 times (group P3) (Figure 2). Therefore, the 336 types of proteins whose expression was eliminated by passage were deemed likely to have been derived from the different types of cells contained in the SVF.

The amount of protein quantified in this paper is a theoretical value estimated based on the emPAI function of the Scaffold software program. The ratio of the number of measured peptides to the number of theoretical peptides is linearly related to the logarithm of the protein concentration, and the number obtained by subtracting 1 from the index of the peptide number ratio was defined as the emPAI. The larger the emPAI value, the greater the amount of protein. We recently published a paper on the correlation between different emPAI values (>0, >1, >2, >3, >5, and >10) and the results of protein expression analyses. The results showed that, for an emPAI value > 10, the presence of protein can be detected with a high probability, even if the number of samples is *n* = 1 [25]. Proteins quantified using emPAI were listed from the top in the tables showing the GO analysis results (Tables 1 and 2) in descending order of concentration. Since the data of this study were derived from a single protein expression analysis obtained from mMSC-ATs of three mice, the data reliability must be carefully considered.

### 3.4. The Biological Processes, Cellular Components, and Molecular Function of Proteins Identified from mMSC-ATs (P0)

The biological processes of proteins were analyzed using the Mascot software program with the SwissProt 2017 database.

#### 3.4.1. Biological Processes

**Figure 3:**
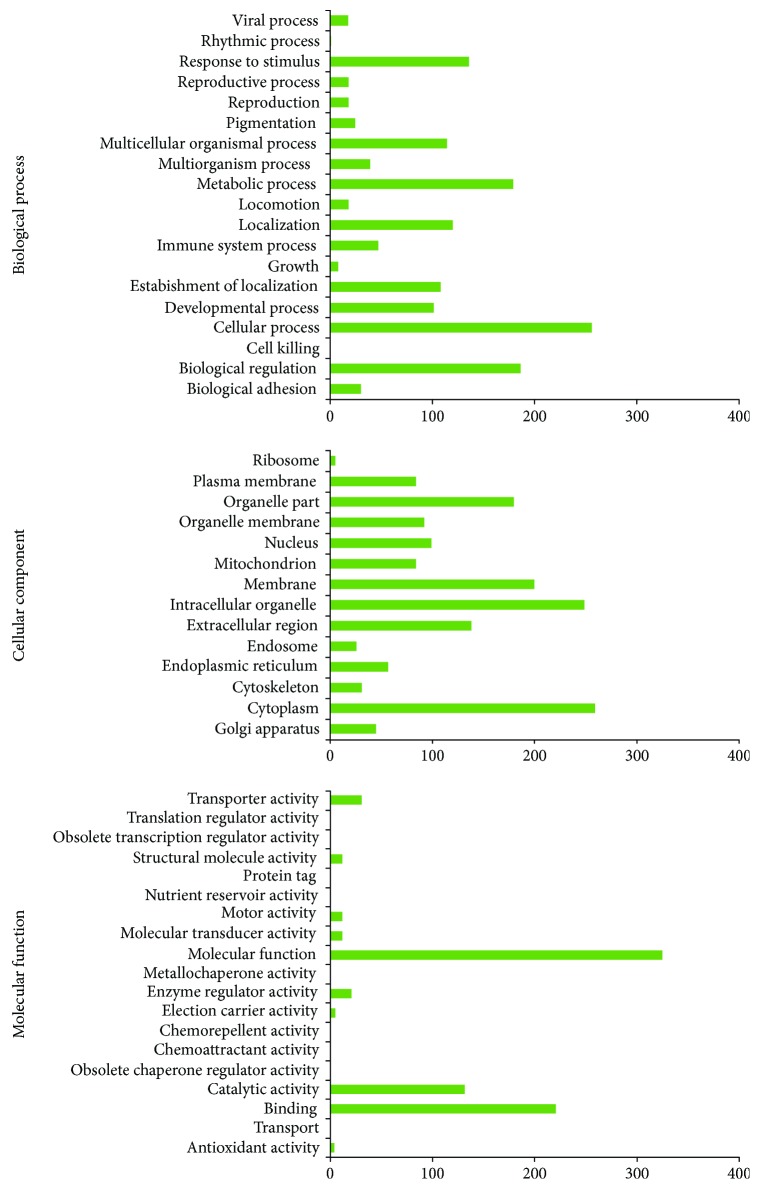
The biological processes, cellular components, and molecular function of the mouse primary cultured cell (P0) proteins (as determined by GO). The PCA of proteome dynamics based on the protein information generated by high-resolution mass spectrometry. The ordinate indicates the biological function, cellular component, and molecular function of the protein. The abscissa indicates the number of identified proteins. The names of the proteins classified in Table 3 are listed by their detailed molecular functions.

Antiviral protein was detected as a protein component of mMSC-ATs (P0) (rich in components of SVF). Isg15, Npc2, Ripk3, Fmr1, Dag1, Vps16, Gas6, Stmn1, Vapb, Tri25, Eea1, Asc, Ifm2, Bst2, Apoe, Ltor5, Oas1a, and Nect2 were detected as factors related to the viral process (Figure 3).

Stml2 (CD4-positive, alpha-beta T cell activation), CD36, CD180 (B cell proliferation), CD47 (opsonization), and CD166 (adaptive immune response) were detected as factors related to the CD cell surface markers related to the immune system. Cats, Ha1b, Erap1, Mdr1a, Psb8, and Ha11 were detected as factors related to the MHC class antigen and thereby related to the immune system. Csf1 (osteoclast differentiation), CD109 (osteoclast fusion), and Rab35 (antigen processing and presentation) were detected as factors related to the bone immune system. Isg15, Tri25, Ifm2, Bst2, Oas1a, and Asc were detected as factors related to the antiviral immune system. Tgbr3, Plek (hematopoietic progenitor cell differentiation), Ripk3 (T cell differentiation in thymus), Gas6 (macrophage cytokine production), Sfxn1 (erythrocyte differentiation), Ada (T cell activation, B cell differentiation), Itam (activated T cell proliferation), Pfd1 (B cell activation), Sbds (leukocyte chemotaxis), Aimp1 (leukocyte migration), Armc6 (hematopoietic progenitor cell differentiation), Glrx5 (hemopoiesis), Hdac7 (B cell activation, B cell differentiation), Psn1 (T cell activation), Ada10 (monocyte activation), and Tpd52 (B cell differentiation) were detected as factors related to hematopoiesis. A4, Ada15, Ptms (immune system process), Fcgrn (antigen processing and presentation), Il4ra, Ilf2, Ic1 (complement activation), Olr1, and Snp23 (histamine secretion by mast cell) were detected as factors related to the immune response. Stat1 was detected as an interferon-related immune response factor. Those proteins were detected as factors related to the immune system process (Figure 3).

Csf1 (mammary duct terminal end bud growth), Tgbr3 (cardiac muscle cell proliferation), A4 (synaptic growth at neuromuscular junction), Ada15 (tissue regeneration), CD166 (axon extension involved in axon guidance, neuron projection extension), Tbl1r (multicellular organism growth), Ninj1 (tissue regeneration), and Sbds (inner cell mass cell proliferation) were detected as factors related to growth in biological processes (Figure 3).

#### 3.4.2. Cellular Components

Stml2 (T cell receptor complex), Stx7 (immunological synapse), Ha1b (MHC class I protein complex), Ha11 (MHC class I protein complex), CD166 (T cell receptor complex), At2b1 (apical plasma membrane), Gna11 (heterotrimeric G-protein complex), Home3 (postsynaptic membrane), Ampn, Rdh11, Nsf1c, Bag3, Csf1, Vatc1, Necp2, CD109, CD36, Ece1, Tgbr3, A4, Ripk3, Gdn, Erln2, Aaat, Suca, Snp23, Prio, Ly6a, Plp2, Plek, Tpbg, Fmr1, Plin4, Ldlr, Pdc10, Dag1, Rap2a, Vatg1, Pp2ba, Rab35, Il1ap, Eea1, Fcgrn, Ada, Cdv3, Erap1, Ifm2, Bst2, CD180, Il4ra, Itam, Osmr, P2rx4, Serc1, Tradd, CD97, Rab4b, Rab8a, Sucb2, Apoe, Bap31, Cdipt, Cn37, Crk, Dnjb4, Ggt5, Mdr1a, Piez1, Praf1, Psn1, S39ae, Stx2, Tnr12, Vmp1, Fen1, CD47, Olr1, Pygl, Wwox, Ctl1, Ly6c1, Nect2, and Ttyh2 (plasma membrane) were detected as factors related to growth in the plasma membrane of the cellular component (Figure 3).

#### 3.4.3. Molecular Functions

SRXN1 (antioxidant activity), TXD17 (peroxidase activity), PGFS (thioredoxin peroxidase activity), and APOE (antioxidant activity) are proteins that are expressed on the plasma membrane. These proteins have been reported to be factors related to both growth and the antioxidant activity according to a classification of the molecular functions (Figure 3). The proteins of mMSC-ATs (P0) are listed in Table 3 with their molecular functions described in detail.

### 3.5. Relationship of the Quantitative Value (Normalized emPAI) per Housekeeping Gene of mMSC-ATs (P0 and P3)

**Figure 4:**
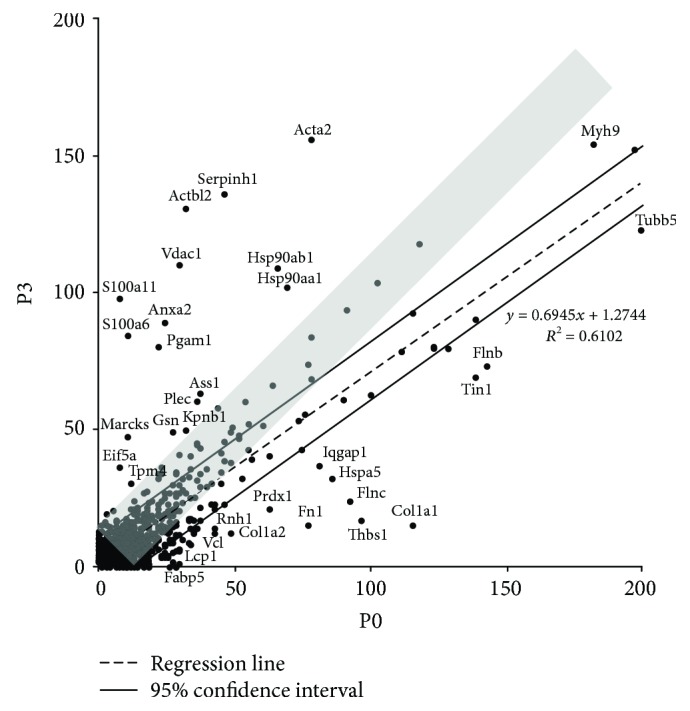
A scatter plot of the quantitative value (normalized emPAI) per housekeeping gene. A scatter plot showing correlation (*R* ^2^ = 0.6102; gray band indicates “*R* ^2^ = 1”) between the quantitative value of the mouse primary cultured cells (P0) and cells passaged 3 times (P3) (*n* = 1139). The dotted line is the regression line. The two lines indicate the 95% confidence interval. Each dot shows the abbreviated name of the protein.

The quantitative values of the proteins expressed in both mMSC-ATs (P0) and mMSC-ATs (P3) were represented with a scatter plot (*y*-axis = P3, *x*-axis = P0). The average quantitative value of P3-expressed proteins decreased to 69.4% compared to P0-expressed proteins (Atp5f1, B2m, Hprt1, Rplp1, Ppia, Rps18, Pgk1, Tfrc, Ywhaz, and Gapdh; Supplementary Figure 1). The quantitative values of Tubb5, Flnb, Tln1, Col1a1, Iqgap1, Hspa5, Flnc, Thbs1, Fn1, Prdx1, Rnh1, Col1a2, Vcl, Lcp1, and Fabp5 were higher at P0 than at P3. The quantitative value of Act2, Serponh1, Hsp90ab1, Hsp90aa1, Actbl2, Vdac1, S100a11, Anxa2, S100a6, Pgam1, argininosuccinate synthase (Ass1, which is regulated by hypoxia-inducible factor 1*α* (Hif1*α*)), Plec, Kpnb1, Gsn, Marcks, Eif5a, and Tpm4 was higher at P3 than at P0 (Figure 4).

We previously examined the protein components expressed by human MSC-ATs (hMSC-ATs) cultured in the clinical medium not containing FBS and hMSC-ATs cultured in DMEM containing FBS [25]. Based on the results of the protein expression analysis, the expression of TLN1, FLNC, and ASS1 was higher in hMSC-ATs cultured in DMEM containing FBS than in those cultured in the clinical culture medium. Therefore, the increased expression of Tln1, Flnc, and Ass1 protein at P3 compared with P0 is not caused by FBS. Regarding the change in the expression of Ass1, the activation level of Hif1*α* was considered to be higher at P0 than at P3, because the oxygen concentration is lower *in vivo* than *in vitro* [29, 30]. Therefore, the results of this study reflect not only the effect of cell division frequency but also the influence of oxygen concentration. When cultured cells are planted in a living body in a low-oxygen environment, the hypoxic response may be activated. Therefore, in order to interpret the results of this study more accurately, we must obtain data on the protein expression of MSCs cultured under hypoxic and high-oxygen conditions.

## 4. Conclusions

The functions of proteins classified by the GO analysis were quantified using the LC-MS/MS measurement system for the amount of proteins and components contained in primary cultured cells of mMSC-ATs and cells passaged three times. The ability of mMSC-ATs to differentiate into expression markers of cells, fat, and osteoblasts did not change, even after three passages. However, the protein expression decreased to 69.4%. The proteins whose expression levels decreased after three passages included Ass1 among the Hif-related proteins. Furthermore, it was revealed that 336 kinds of proteins are specifically expressed in primary cultured MSC-ATs. In conclusion, MSC-ATs used as therapeutic cells retained their cell properties after three passages but showed a decreased protein expression on LC-MS/MS.

## Figures and Tables

**Table 1:** Identification of endogenous proteins contained in mMSC-AT_P0 (primary cultured cells).

UniProt/Swiss-Prot ID	Description	Protein score^a^	Protein mass (kDa)	pI^b^	Num. of matches^c^	Num. of significant matches^d^	Num. of sequences^e^	Num. of significant sequences^f^	Num. of unique sequences^g^	Sequence coverage^h^	emPAI^i^
FLNA_MOUSE	Filamin-A	2702	281046	5.68	220	138	87	56	81	0.54	1.34
FLNB_MOUSE	Filamin-B	1877	277651	5.46	164	94	81	51	73	0.52	1.23
FLNC_MOUSE	Filamin-C	1471	290937	5.63	115	68	57	33	11	0.39	0.68
MYH9_MOUSE	Myosin-9	2689	226232	5.54	232	114	116	57	100	0.64	2.27
TLN1_MOUSE	Talin-1	2390	269653	5.84	147	98	72	53	52	0.49	1.39
IQGA1_MOUSE	Ras GTPase-activating-like protein IQGAP1	1740	188624	6.07	97	56	53	33	53	0.47	1.13
CLH1_MOUSE	Clathrin heavy chain 1	1668	191435	5.48	114	80	64	49	64	0.6	2.26
CH60_MOUSE	60 kDa heat shock protein, mitochondrial	1558	60917	5.91	75	58	29	22	29	0.66	5.35
DYHC1_MOUSE	Cytoplasmic dynein 1 heavy chain 1	1460	531710	6.03	121	67	85	48	85	0.3	0.47
ACTN1_MOUSE	Alpha-actinin-1	1455	103004	5.23	99	55	44	29	26	0.63	2.67
ACTN4_MOUSE	Alpha-actinin-4	914	104911	5.25	76	36	39	23	21	0.56	1.5
HSP7C_MOUSE	Heat shock cognate 71 kDa protein	1427	70827	5.37	147	72	34	26	27	0.72	4.53
GRP78_MOUSE	78 kDa glucose-regulated protein	1101	72377	5.07	114	54	35	23	32	0.64	2.77
GRP75_MOUSE	Stress-70 protein, mitochondrial	851	73416	5.81	65	36	22	17	21	0.46	1.79
HS71A_MOUSE	Heat shock 70 kDa protein 1A	246	70036	5.53	34	13	12	7	8	0.34	0.52
NSF_MOUSE	Vesicle-fusing ATPase	98	82561	6.52	8	4	7	4	7	0.15	0.22
TBB6_MOUSE	Tubulin beta-6 chain	517	50058	4.8	59	35	24	17	10	0.79	3.86
VIME_MOUSE	Vimentin	1321	53655	5.06	170	69	45	30	45	0.76	9.3
ENPL_MOUSE	Endoplasmin	1314	92418	4.74	108	60	44	32	20	0.58	4.09
HS90A_MOUSE	Heat shock protein HSP 90-alpha	1047	84735	4.93	93	47	38	22	25	0.56	2.27
ENOA_MOUSE	Alpha-enolase	1304	47111	6.37	95	58	23	17	23	0.74	6
FAS_MOUSE	Fatty acid synthase	1264	272257	6.13	89	59	61	44	61	0.45	1.03
G3P_MOUSE	Glyceraldehyde-3-phosphate dehydrogenase	1238	35787	8.44	94	50	19	13	15	0.76	9.22
KPYM_MOUSE	Pyruvate kinase PKM	1232	57808	7.18	98	58	37	30	12	0.74	13.41
ACTBL_MOUSE	Beta-actin-like protein 2	361	41977	5.3	52	22	13	5	4	0.51	1.44
TSP1_MOUSE	Thrombospondin-1	1160	129564	4.72	117	62	35	17	34	0.36	0.91
ATPA_MOUSE	ATP synthase subunit alpha, mitochondrial	1118	59716	9.22	64	44	24	18	16	0.58	3.34
SPB6_MOUSE	Serpin B6	1096	42571	5.53	49	36	21	14	21	0.71	4.79
ILEUA_MOUSE	Leukocyte elastase inhibitor A	99	42548	5.85	4	4	3	3	3	0.11	0.34
GDN_MOUSE	Glia-derived nexin	84	44179	9.85	6	4	5	4	5	0.16	0.46
FINC_MOUSE	Fibronectin	1089	272368	5.39	97	52	57	34	57	0.4	0.69
EF2_MOUSE	Elongation factor 2	1085	95253	6.41	91	53	47	28	46	0.66	2.42
UBA1_MOUSE	Ubiquitin-like modifier-activating enzyme 1	1077	117734	5.43	55	39	26	19	26	0.45	1.11
RINI_MOUSE	Ribonuclease inhibitor	991	49784	4.69	48	34	23	17	19	0.81	3.14
PDIA1_MOUSE	Protein disulfide-isomerase	965	57023	4.77	91	48	34	21	34	0.62	3.99
ATPB_MOUSE	ATP synthase subunit beta, mitochondrial	853	56265	5.19	65	39	28	22	26	0.81	4.5
TCPA_MOUSE	T-complex protein 1 subunit alpha	833	60411	5.82	44	25	23	12	23	0.64	2.02
1433Z_MOUSE	14-3-3 protein zeta/delta	831	27754	4.73	46	29	15	11	12	0.6	4.16
1433B_MOUSE	14-3-3 protein beta/alpha	539	28069	4.77	28	19	13	10	3	0.63	3.37
CO1A1_MOUSE	Collagen alpha-1(I) chain	825	137948	5.65	141	65	42	24	25	0.53	1.27
HSP74_MOUSE	Heat shock 70 kDa protein 4	817	94073	5.15	56	29	26	18	25	0.46	1.22
HS105_MOUSE	Heat shock protein 105 kDa	118	96346	5.39	14	6	12	5	11	0.25	0.24
LRP1_MOUSE	Prolow-density lipoprotein receptor-related protein 1	815	504411	5.14	75	39	54	26	54	0.22	0.24
WDR1_MOUSE	WD repeat-containing protein 1	796	66365	6.11	35	23	18	12	18	0.57	1.13
LDHA_MOUSE	L-Lactate dehydrogenase A chain	752	36475	7.62	81	28	19	11	19	0.79	3.92
ANXA1_MOUSE	Annexin A1	750	38710	6.97	50	27	21	11	21	0.61	2.26
MOES_MOUSE	Moesin	737	67725	6.22	73	32	34	15	23	0.61	2.03
TKT_MOUSE	Transketolase	732	67588	7.23	53	32	26	16	26	0.69	2.04
SERPH_MOUSE	Serpin H1	731	46504	8.88	56	35	23	16	23	0.68	4.01
SC31A_MOUSE	Protein transport protein Sec31A	707	133486	6.3	30	23	15	13	15	0.27	0.55
ALDOA_MOUSE	Fructose-bisphosphate aldolase A	674	39331	8.31	56	33	23	15	23	0.83	5.04
VDAC1_MOUSE	Voltage-dependent anion-selective channel protein 1	667	32331	8.55	31	23	11	9	11	0.67	2.61
VDAC2_MOUSE	Voltage-dependent anion-selective channel protein 2	454	31713	7.44	25	19	10	8	10	0.54	1.84
RPN2_MOUSE	Dolichyl-diphosphooligosaccharide–protein glycosyltransferase subunit 2	666	69020	5.54	34	24	19	16	19	0.52	1.63
ASSY_MOUSE	Argininosuccinate synthase	660	46555	8.36	54	25	24	12	22	0.75	2.5
SYAC_MOUSE	Alanine–tRNA ligase, cytoplasmic	658	106841	5.45	46	25	29	18	29	0.55	1.02
UGGG1_MOUSE	UDP-glucose:glycoprotein glucosyltransferase 1	656	176323	5.4	34	20	24	15	24	0.3	0.43
ANXA5_MOUSE	Annexin A5	647	35730	4.83	65	34	19	12	18	0.71	4.71
ANXA3_MOUSE	Annexin A3	313	36362	5.5	29	16	17	12	16	0.64	2.93
ANXA6_MOUSE	Annexin A6	196	75837	5.34	17	11	12	8	12	0.27	0.55
ANX11_MOUSE	Annexin A11	53	54045	7.53	4	3	3	2	3	0.11	0.17
CO1A2_MOUSE	Collagen alpha-2(I) chain	645	129478	9.27	61	38	29	17	29	0.44	0.85
6PGD_MOUSE	6-Phosphogluconate dehydrogenase, decarboxylating	642	53213	6.81	38	21	19	14	19	0.63	2
SPTN1_MOUSE	Spectrin alpha chain, nonerythrocytic 1	639	284422	5.2	61	37	45	25	45	0.32	0.45
AT1A1_MOUSE	Sodium/potassium-transporting ATPase subunit alpha-1	619	112910	5.3	47	26	25	15	25	0.34	0.74
GDIB_MOUSE	Rab GDP dissociation inhibitor beta	615	50505	5.93	40	22	18	10	13	0.58	1.48
GDIA_MOUSE	Rab GDP dissociation inhibitor alpha	274	50489	4.96	25	13	13	9	8	0.49	1.28
EF1G_MOUSE	Elongation factor 1-gamma	613	50029	6.31	43	29	21	14	21	0.67	2.48
TCPQ_MOUSE	T-complex protein 1 subunit theta	611	59518	5.44	35	19	20	12	20	0.53	1.32
MDHM_MOUSE	Malate dehydrogenase, mitochondrial	603	35589	8.93	39	27	16	11	16	0.58	3.55
PDIA4_MOUSE	Protein disulfide-isomerase A4	602	71938	5.16	50	29	21	13	21	0.45	1.39
CAP1_MOUSE	Adenylyl cyclase-associated protein 1	594	51532	7.16	58	28	24	14	24	0.73	2.1
IMB1_MOUSE	Importin subunit beta-1	584	97122	4.68	33	24	24	17	24	0.41	1.08
CAPG_MOUSE	Macrophage-capping protein	263	39216	6.73	16	8	6	5	1	0.3	0.7
ESTD_MOUSE	S-Formylglutathione hydrolase	576	31299	6.7	50	28	17	12	17	0.87	5.39
CAN2_MOUSE	Calpain-2 catalytic subunit	566	79822	4.86	37	28	23	20	20	0.58	2
VATB2_MOUSE	V-type proton ATPase subunit B, brain isoform	562	56515	5.57	40	27	20	14	20	0.69	2.03
VPS35_MOUSE	Vacuolar protein sorting-associated protein 35	561	91655	5.28	32	21	16	10	16	0.3	0.58
LYAG_MOUSE	Lysosomal alpha-glucosidase	561	106180	5.53	34	26	20	16	20	0.37	0.88
ACLY_MOUSE	ATP-citrate synthase	559	119651	7.13	34	23	27	18	27	0.39	0.94
FPPS_MOUSE	Farnesyl pyrophosphate synthase	554	40556	5.49	31	18	17	10	17	0.61	1.79
COPG1_MOUSE	Coatomer subunit gamma-1	553	97450	5.23	35	22	24	18	20	0.46	1.16
IDHC_MOUSE	Isocitrate dehydrogenase [NADP] cytoplasmic	552	46644	6.73	35	25	17	10	16	0.57	1.92
VIGLN_MOUSE	Vigilin	550	141655	6.43	40	24	27	15	27	0.34	0.56
P4HA1_MOUSE	Prolyl 4-hydroxylase subunit alpha-1	546	60872	5.62	33	18	17	12	17	0.5	1.44
CATB_MOUSE	Cathepsin B	542	37256	5.57	39	24	13	11	13	0.6	3.26
VINC_MOUSE	Vinculin	520	116644	5.77	53	24	33	19	33	0.41	0.98
CNDP2_MOUSE	Cytosolic nonspecific dipeptidase	516	52734	5.43	41	23	20	15	20	0.67	2.27
SEPT2_MOUSE	Septin-2	515	41499	6.1	22	16	14	9	14	0.56	1.46
DPYL2_MOUSE	Dihydropyrimidinase-related protein 2	509	62239	5.95	43	25	25	15	22	0.73	1.73
DPYL3_MOUSE	Dihydropyrimidinase-related protein 3	203	61897	6.04	16	9	8	4	5	0.21	0.31
TAGL2_MOUSE	Transgelin-2	509	22381	8.39	42	28	14	11	14	0.8	8.1
TCPH_MOUSE	T-complex protein 1 subunit eta	501	59614	7.95	34	22	18	15	18	0.53	2.06
FRIL1_MOUSE	Ferritin light chain 1	500	20790	5.66	18	15	6	4	6	0.49	1.7
NCPR_MOUSE	NADPH–cytochrome P450 reductase	499	76995	5.34	19	15	12	8	12	0.33	0.54
TCPZ_MOUSE	T-complex protein 1 subunit zeta	491	57968	6.63	30	18	19	12	19	0.55	1.37
VATA_MOUSE	V-type proton ATPase catalytic subunit A	481	68283	5.42	43	23	27	14	27	0.6	1.35
XDH_MOUSE	Xanthine dehydrogenase/oxidase	475	146468	7.62	28	23	16	14	16	0.24	0.54
PDC6I_MOUSE	Programmed cell death 6-interacting protein	475	95964	6.15	25	17	17	12	17	0.35	0.76
CAND1_MOUSE	Cullin-associated NEDD8-dissociated protein 1	473	136245	5.52	26	19	20	14	20	0.33	0.64
COF1_MOUSE	Cofilin-1	473	18548	8.22	30	18	11	9	9	0.61	6.37
COF2_MOUSE	Cofilin-2	182	18698	7.66	20	10	6	4	4	0.49	1.42
LG3BP_MOUSE	Galectin-3-binding protein	471	64450	5	17	12	10	6	9	0.3	0.47
VAT1_MOUSE	Synaptic vesicle membrane protein VAT-1 homolog	471	43069	5.95	44	25	21	14	21	0.65	3.69
TCPD_MOUSE	T-complex protein 1 subunit delta	467	58030	8.24	35	17	20	9	20	0.59	0.91
AMPN_MOUSE	Aminopeptidase N	465	109582	5.62	26	21	17	16	17	0.3	0.84
PPIA_MOUSE	Peptidyl-prolyl cis-trans isomerase A	465	17960	7.74	33	21	11	8	11	0.7	11.36
MDHC_MOUSE	Malate dehydrogenase, cytoplasmic	464	36488	6.16	21	14	10	6	10	0.52	1.22
G6PI_MOUSE	Glucose-6-phosphate isomerase	462	62727	8.14	29	18	15	10	8	0.4	1.08
PDIA3_MOUSE	Protein disulfide-isomerase A3	449	56643	5.88	115	29	32	16	32	0.64	2.77
THIO_MOUSE	Thioredoxin	445	11668	4.8	24	16	7	6	7	0.78	7.1
RLA0_MOUSE	60S acidic ribosomal protein P0	441	34195	5.91	30	21	14	10	14	0.56	2.8
ESYT1_MOUSE	Extended synaptotagmin-1	437	121478	5.63	20	17	13	11	13	0.22	0.46
2AAA_MOUSE	Serine/threonine-protein phosphatase 2A 65 kDa regulatory subunit A alpha isoform	437	65281	5	20	14	17	12	17	0.46	1.15
PTBP1_MOUSE	Polypyrimidine tract-binding protein 1	437	56443	8.47	22	11	8	5	8	0.36	0.68
AT2A2_MOUSE	Sarcoplasmic/endoplasmic reticulum calcium ATPase 2	436	114784	5.23	33	20	20	12	20	0.32	0.55
SPTB2_MOUSE	Spectrin beta chain, nonerythrocytic 1	429	274052	5.4	52	21	39	20	39	0.25	0.38
SCOT1_MOUSE	Succinyl-CoA:3-ketoacid coenzyme A transferase 1, mitochondrial	428	55953	8.73	29	20	14	12	14	0.5	1.63
CALR_MOUSE	Calreticulin	419	47965	4.33	43	26	19	11	19	0.6	1.84
CKAP4_MOUSE	Cytoskeleton-associated protein 4	418	63654	5.46	48	27	25	13	25	0.54	1.86
MYOF_MOUSE	Myoferlin	417	233177	5.83	44	17	30	12	30	0.24	0.24
GSTP1_MOUSE	Glutathione S-transferase P 1	417	23594	7.68	21	14	9	7	9	0.58	3.82
PLEC_MOUSE	Plectin	416	533861	5.74	66	21	52	16	52	0.18	0.13
TCPB_MOUSE	T-complex protein 1 subunit beta	414	57441	5.97	27	15	20	13	20	0.56	1.57
KAD1_MOUSE	Adenylate kinase isoenzyme 1	410	21526	5.67	15	13	9	8	6	0.64	3.63
PRDX6_MOUSE	Peroxiredoxin-6	405	24855	5.71	36	18	16	11	16	0.75	5.23
RL9_MOUSE	60S ribosomal protein L9	401	21868	9.96	12	10	5	4	5	0.48	1.13
UGDH_MOUSE	UDP-glucose 6-dehydrogenase	398	54797	7.49	33	18	22	14	22	0.66	1.9
CATD_MOUSE	Cathepsin D	389	44925	6.71	35	21	15	9	15	0.55	2.33
NIBL1_MOUSE	Niban-like protein 1	388	84765	5.65	23	15	18	13	18	0.36	0.9
TXND5_MOUSE	Thioredoxin domain-containing protein 5	386	46386	5.51	17	14	8	5	8	0.32	0.57
TSP2_MOUSE	Thrombospondin-2	385	129798	4.61	44	19	22	10	21	0.28	0.38
MAOX_MOUSE	NADP-dependent malic enzyme	384	63913	7.16	15	11	14	10	14	0.49	0.92
ARF4_MOUSE	ADP-ribosylation factor 4	382	20384	6.59	23	15	11	8	5	0.83	6.52
COPB2_MOUSE	Coatomer subunit beta′	381	102384	5.17	33	20	24	15	24	0.43	0.85
PRDX1_MOUSE	Peroxiredoxin-1	379	22162	8.26	87	35	20	14	19	0.8	15.25
PRDX2_MOUSE	Peroxiredoxin-2	316	21765	5.2	16	11	8	7	8	0.45	2.77
PRDX4_MOUSE	Peroxiredoxin-4	152	31033	6.67	18	7	11	5	10	0.52	0.95
PLSL_MOUSE	Plastin-2	378	70105	5.2	35	21	22	13	20	0.48	1.17
GPNMB_MOUSE	Transmembrane glycoprotein NMB	378	63635	7.55	27	16	10	5	10	0.22	0.48
GELS_MOUSE	Gelsolin	371	85888	5.83	32	21	17	13	17	0.45	0.98
THIC_MOUSE	Acetyl-CoA acetyltransferase, cytosolic	371	41271	7.16	20	12	12	8	12	0.59	1.24
ECHA_MOUSE	Trifunctional enzyme subunit alpha, mitochondrial	371	82617	9.24	24	16	16	10	16	0.44	0.66
MYO1C_MOUSE	Unconventional myosin-Ic	370	121868	9.41	34	17	29	14	29	0.4	0.62
PDIA6_MOUSE	Protein disulfide-isomerase A6	369	48070	5	29	22	14	12	14	0.48	1.83
EIF3L_MOUSE	Eukaryotic translation initiation factor 3 subunit L	368	66570	6.01	16	11	7	5	7	0.21	0.37
MVP_MOUSE	Major vault protein	365	95865	5.43	30	16	17	10	17	0.35	0.55
HYOU1_MOUSE	Hypoxia-upregulated protein 1	362	111112	5.12	18	10	15	8	15	0.27	0.35
FABP5_MOUSE	Fatty acid-binding protein, epidermal	361	15127	6.14	35	19	8	6	8	0.66	7.75
IPO5_MOUSE	Importin-5	358	123511	4.82	42	28	25	19	25	0.44	1.11
TCPE_MOUSE	T-complex protein 1 subunit epsilon	356	59586	5.72	27	17	17	10	17	0.45	1.16
OST48_MOUSE	Dolichyl-diphosphooligosaccharide–protein glycosyltransferase 48 kDa subunit	355	48997	5.52	21	13	11	5	11	0.38	0.81
SYRC_MOUSE	Arginine–tRNA ligase, cytoplasmic	354	75625	7.48	19	11	14	7	14	0.31	0.56
NB5R3_MOUSE	NADH-cytochrome b5 reductase 3	354	34106	8.55	26	18	12	10	12	0.68	2.82
NDKB_MOUSE	Nucleoside diphosphate kinase B	353	17352	6.97	30	14	10	4	6	0.74	3.15
NDKA_MOUSE	Nucleoside diphosphate kinase A	171	17197	6.84	22	11	7	5	3	0.59	4.32
CO5A1_MOUSE	Collagen alpha-1(V) chain	351	183564	4.86	13	11	8	6	8	0.1	0.15
RLA2_MOUSE	60S acidic ribosomal protein P2	348	11644	4.42	13	9	5	5	5	0.7	4.72
AK1A1_MOUSE	Alcohol dehydrogenase [NADP(+)]	344	36564	6.9	23	16	16	11	16	0.64	2.92
ALDR_MOUSE	Aldose reductase	114	35709	6.71	14	6	7	4	7	0.26	0.59
PCBP2_MOUSE	Poly(rC)-binding protein 2	343	38197	6.33	32	17	12	8	2	0.55	1.66
NEDD4_MOUSE	E3 ubiquitin-protein ligase NEDD4	343	102642	5.12	29	17	18	11	18	0.33	0.57
UAP1L_MOUSE	UDP-N-acetylhexosamine pyrophosphorylase-like protein 1	342	56578	5.27	28	15	19	11	17	0.55	1.25
UAP1_MOUSE	UDP-N-acetylhexosamine pyrophosphorylase	87	58572	6.04	4	3	2	2	1	0.06	0.15
CLIC1_MOUSE	Chloride intracellular channel protein 1	342	26996	5.09	24	13	14	8	13	0.72	2.41
GSLG1_MOUSE	Golgi apparatus protein 1	342	133646	6.45	20	11	16	7	11	0.2	0.28
DNJC3_MOUSE	DnaJ homolog subfamily C member 3	341	57428	5.61	9	5	6	3	6	0.29	0.24
FSCN1_MOUSE	Fascin	340	54474	6.44	24	10	15	8	15	0.51	0.84
SODM_MOUSE	Superoxide dismutase [Mn], mitochondrial	340	24588	8.8	12	9	5	4	5	0.46	0.96
PROF1_MOUSE	Profilin-1	339	14948	8.46	65	22	11	7	11	0.78	10.68
MBB1A_MOUSE	Myb-binding protein 1A	339	151942	9.08	21	15	15	10	15	0.18	0.32
CISY_MOUSE	Citrate synthase, mitochondrial	338	51703	8.72	23	14	16	11	16	0.56	1.63
SAP_MOUSE	Prosaposin	338	61381	5.07	45	16	19	10	19	0.49	1.11
ADT1_MOUSE	ADP/ATP translocase 1	278	32883	9.73	25	9	16	7	9	0.61	1.42
CPNS1_MOUSE	Calpain small subunit 1	337	28445	5.41	14	9	7	5	7	0.41	1.07
UGPA_MOUSE	UTP–glucose-1-phosphate uridylyltransferase	335	56944	7.18	14	9	9	6	9	0.3	0.55
NACAM_MOUSE	Nascent polypeptide-associated complex subunit alpha, muscle-specific form	330	220364	9.39	20	12	8	3	8	0.06	0.06
DLDH_MOUSE	Dihydrolipoyl dehydrogenase, mitochondrial	329	54238	7.99	16	12	10	7	10	0.39	0.71
HNRPF_MOUSE	Heterogeneous nuclear ribonucleoprotein F	327	45701	5.31	17	10	10	6	8	0.42	0.73
OAT_MOUSE	Ornithine aminotransferase, mitochondrial	326	48324	6.19	25	14	9	6	9	0.4	0.68
DHB12_MOUSE	Very-long-chain 3-oxoacyl-CoA reductase	324	34719	9.55	18	13	12	9	12	0.56	1.93
DHE3_MOUSE	Glutamate dehydrogenase 1, mitochondrial	323	61298	8.05	36	16	16	9	16	0.44	0.85
CATS_MOUSE	Cathepsin S	323	38449	6.51	18	10	12	7	12	0.53	1.13
ITB5_MOUSE	Integrin beta-5	322	87851	5.81	18	14	10	7	10	0.25	0.4
CATL1_MOUSE	Cathepsin L1	321	37523	6.37	13	10	8	6	8	0.5	1.43
PSMD1_MOUSE	26S proteasome non-ATPase regulatory subunit 1	321	105663	5.25	20	12	15	10	15	0.29	0.49
RPN1_MOUSE	Dolichyl-diphosphooligosaccharide–protein glycosyltransferase subunit 1	320	68486	6.02	32	19	19	11	19	0.45	0.96
LYOX_MOUSE	Protein-lysine 6-oxidase	319	46671	8.73	20	13	8	5	8	0.37	0.56
AOFA_MOUSE	Amine oxidase [flavin-containing] A	319	59564	7.9	19	14	12	7	12	0.39	0.63
PSD12_MOUSE	26S proteasome non-ATPase regulatory subunit 12	315	52861	6.66	22	12	15	7	15	0.43	0.74
COCA1_MOUSE	Collagen alpha-1(XII) chain	315	340004	5.47	39	19	24	14	24	0.15	0.19
ALDH2_MOUSE	Aldehyde dehydrogenase, mitochondrial	315	56502	7.53	39	20	19	14	19	0.62	2.03
PP1A_MOUSE	Serine/threonine-protein phosphatase PP1-alpha catalytic subunit	314	37516	5.94	15	12	10	7	10	0.37	1.17
TPIS_MOUSE	Triosephosphate isomerase	312	32171	5.56	36	14	14	9	14	0.64	2.63
GSTM1_MOUSE	Glutathione S-transferase Mu 1	311	25953	7.71	32	13	12	6	9	0.61	2.05
GSTM2_MOUSE	Glutathione S-transferase Mu 2	195	25700	6.9	17	8	11	6	8	0.6	1.62
MPRI_MOUSE	Cation-independent mannose-6-phosphate receptor	309	273639	5.47	30	18	23	16	23	0.18	0.28
CALX_MOUSE	Calnexin	307	67236	4.5	23	15	16	9	16	0.37	0.86
EIF3D_MOUSE	Eukaryotic translation initiation factor 3 subunit D	305	63948	5.79	11	8	7	5	7	0.25	0.39
TPM4_MOUSE	Tropomyosin alpha-4 chain	203	28450	4.65	21	9	11	7	10	0.46	1.77
CATZ_MOUSE	Cathepsin Z	303	33974	6.13	11	9	4	4	4	0.26	0.63
GNAI2_MOUSE	Guanine nucleotide-binding protein G(i) subunit alpha-2	255	40463	5.28	19	9	11	6	5	0.38	0.85
STOM_MOUSE	Erythrocyte band 7 integral membrane protein	300	31355	6.45	18	10	7	5	7	0.38	0.94
ITB1_MOUSE	Integrin beta-1	300	88173	5.68	29	17	18	10	18	0.41	0.61
PSA7_MOUSE	Proteasome subunit alpha type-7	296	27838	8.59	15	6	8	4	8	0.48	0.81
KCRB_MOUSE	Creatine kinase B-type	295	42686	5.4	17	12	10	6	10	0.52	0.79
COPB_MOUSE	Coatomer subunit beta	295	106998	5.69	16	11	14	10	14	0.23	0.48
LEG1_MOUSE	Galectin-1	294	14856	5.32	28	14	9	6	9	0.75	8.03
ANXA2_MOUSE	Annexin A2	293	38652	7.55	24	19	14	11	14	0.49	2.27
XPO2_MOUSE	Exportin-2	289	110384	5.52	10	9	6	5	6	0.12	0.26
EF1D_MOUSE	Elongation factor 1-delta	289	31274	4.91	22	11	11	7	10	0.5	1.53
EF1B_MOUSE	Elongation factor 1-beta	205	24678	4.53	16	9	6	6	5	0.42	1.74
PAI1_MOUSE	Plasminogen activator inhibitor 1	288	45141	6.17	20	10	13	7	13	0.49	0.91
APT_MOUSE	Adenine phosphoribosyltransferase	287	19712	6.31	10	8	6	5	6	0.51	1.84
AKA12_MOUSE	A-kinase anchor protein 12	286	180586	4.39	36	13	22	8	22	0.26	0.2
IMA4_MOUSE	Importin subunit alpha-4	85	57737	4.8	7	4	5	3	2	0.22	0.24
SERA_MOUSE	D-3-Phosphoglycerate dehydrogenase	283	56549	6.12	21	14	14	9	14	0.34	0.94
SND1_MOUSE	Staphylococcal nuclease domain-containing protein 1	282	102025	7.08	25	19	12	10	12	0.26	0.57
ITA5_MOUSE	Integrin alpha-5	280	114971	5.65	23	14	13	9	13	0.2	0.44
RRBP1_MOUSE	Ribosome-binding protein 1	280	172776	9.35	42	16	28	9	28	0.24	0.24
PSB4_MOUSE	Proteasome subunit beta type-4	280	29097	5.47	9	5	7	3	7	0.45	0.53
NUCL_MOUSE	Nucleolin	278	76677	4.69	14	11	10	8	10	0.2	0.55
SFXN3_MOUSE	Sideroflexin-3	277	35384	9.58	12	7	7	3	7	0.4	0.42
ADK_MOUSE	Adenosine kinase	276	40123	5.84	12	11	7	6	7	0.35	0.86
HNRPU_MOUSE	Heterogeneous nuclear ribonucleoprotein U	275	87863	5.92	25	15	17	12	17	0.27	0.77
HXK3_MOUSE	Hexokinase-3	270	100037	5.63	20	12	13	7	13	0.26	0.34
PGM1_MOUSE	Phosphoglucomutase-1	270	61380	6.14	15	11	8	6	8	0.27	0.61
PSB2_MOUSE	Proteasome subunit beta type-2	269	22892	6.52	24	14	10	5	10	0.64	1.95
ICAL_MOUSE	Calpastatin	269	84871	5.37	10	7	5	4	5	0.15	0.22
CD81_MOUSE	CD81 antigen	268	25797	5.54	5	5	2	2	2	0.18	0.62
PPCE_MOUSE	Prolyl endopeptidase	267	80700	5.44	17	8	12	5	12	0.31	0.3
PEBP1_MOUSE	Phosphatidylethanolamine-binding protein 1	267	20817	5.19	16	11	9	7	9	0.78	2.99
MRC2_MOUSE	C-type mannose receptor 2	265	166968	5.65	18	11	14	8	14	0.19	0.22
AP2A2_MOUSE	AP-2 complex subunit alpha-2	264	103951	6.51	24	11	16	7	10	0.28	0.33
AP2A1_MOUSE	AP-2 complex subunit alpha-1	140	107596	6.63	18	7	17	6	11	0.31	0.26
DDB1_MOUSE	DNA damage-binding protein 1	262	126772	5.14	19	11	9	4	9	0.16	0.18
ITAV_MOUSE	Integrin alpha-V	262	115287	5.41	28	10	15	5	15	0.18	0.2
CH10_MOUSE	10 kDa heat shock protein, mitochondrial	260	10956	7.93	18	10	5	2	5	0.52	1.09
COMT_MOUSE	Catechol O-methyltransferase	258	29467	5.52	8	6	4	2	4	0.29	0.33
DDX3X_MOUSE	ATP-dependent RNA helicase DDX3X	257	73056	6.73	21	11	14	8	14	0.36	0.58
PSB7_MOUSE	Proteasome subunit beta type-7	257	29872	8.14	8	6	5	3	5	0.32	0.52
PNPH_MOUSE	Purine nucleoside phosphorylase	257	32256	5.78	17	10	11	9	11	0.67	2.18
PCNA_MOUSE	Proliferating cell nuclear antigen	256	28766	4.66	15	10	9	5	9	0.66	1.06
SYSC_MOUSE	Serine–tRNA ligase, cytoplasmic	255	58352	5.95	17	12	13	8	13	0.41	0.77
AATM_MOUSE	Aspartate aminotransferase, mitochondrial	255	47381	9.13	32	14	18	10	18	0.54	1.41
RL3_MOUSE	60S ribosomal protein L3	254	46081	10.22	30	20	11	8	11	0.36	1.26
FUMH_MOUSE	Fumarate hydratase, mitochondrial	254	54322	9.12	10	8	8	6	8	0.36	0.59
MAP4_MOUSE	Microtubule-associated protein 4	254	117357	4.9	24	13	14	8	14	0.23	0.33
MAP1B_MOUSE	Microtubule-associated protein 1B	254	270089	4.76	14	8	9	3	9	0.06	0.05
SEC13_MOUSE	Protein SEC13 homolog	253	35543	5.15	10	6	4	3	4	0.19	0.42
LAMP1_MOUSE	Lysosome-associated membrane glycoprotein 1	253	43837	8.66	23	14	8	4	8	0.35	0.61
FBLN2_MOUSE	Fibulin-2	253	131746	4.58	27	12	17	9	17	0.23	0.33
GORS2_MOUSE	Golgi reassembly-stacking protein 2	252	47009	4.68	10	8	4	4	4	0.15	0.56
PSB6_MOUSE	Proteasome subunit beta type-6	250	25362	4.97	14	10	9	7	9	0.67	2.14
SERC_MOUSE	Phosphoserine aminotransferase	249	40447	8.15	26	13	13	8	13	0.55	1.28
ERP29_MOUSE	Endoplasmic reticulum resident protein 29	248	28805	5.9	22	13	9	6	9	0.5	1.37
EHD1_MOUSE	EH domain-containing protein 1	247	60565	6.35	22	9	15	9	15	0.47	0.86
RCN3_MOUSE	Reticulocalbin-3	246	37978	4.74	7	6	4	4	4	0.27	0.55
COPE_MOUSE	Coatomer subunit epsilon	245	34545	4.94	10	7	7	6	7	0.38	1.06
PSME1_MOUSE	Proteasome activator complex subunit 1	244	28655	5.73	13	12	5	4	5	0.34	0.78
TADBP_MOUSE	TAR DNA-binding protein 43	244	44519	6.26	9	9	7	7	7	0.34	0.92
C1QBP_MOUSE	Complement component 1 Q subcomponent-binding Protein, mitochondrial	206	30994	4.82	11	7	7	4	1	0.45	0.71
PUR9_MOUSE	Bifunctional purine biosynthesis protein PURH	242	64177	6.3	27	14	18	12	13	0.52	1.18
RCN2_MOUSE	Reticulocalbin-2	242	37248	4.28	10	8	7	5	7	0.39	0.75
PPIB_MOUSE	Peptidyl-prolyl cis-trans isomerase B	241	23699	9.56	20	9	12	4	12	0.54	1.39
PPIC_MOUSE	Peptidyl-prolyl cis-trans isomerase C	140	22780	6.96	5	5	4	4	4	0.26	1.07
RLA1_MOUSE	60S acidic ribosomal protein P1	240	11468	4.28	6	6	3	3	3	0.53	1.9
SNAA_MOUSE	Alpha-soluble NSF attachment protein	239	33168	5.3	15	11	11	8	11	0.56	1.72
KAD2_MOUSE	Adenylate kinase 2, mitochondrial	239	26452	6.96	8	6	5	4	5	0.3	0.87
GDIR1_MOUSE	Rho GDP-dissociation inhibitor 1	238	23393	5.12	16	12	10	8	10	0.51	3.1
CNN2_MOUSE	Calponin-2	237	33134	7.53	20	7	11	5	4	0.65	0.87
SNX9_MOUSE	Sorting nexin-9	236	66504	5.35	17	10	11	6	11	0.36	0.46
CO3A1_MOUSE	Collagen alpha-1(III) chain	236	138858	6.11	28	13	12	6	8	0.15	0.23
IMA5_MOUSE	Importin subunit alpha-5	195	60144	4.93	8	7	4	3	3	0.18	0.32
AMPL_MOUSE	Cytosol aminopeptidase	234	56106	7.62	22	11	13	10	13	0.4	1.1
PABP1_MOUSE	Polyadenylate-binding protein 1	233	70626	9.52	19	10	16	8	16	0.32	0.7
SPA3N_MOUSE	Serine protease inhibitor A3N	233	46688	5.59	4	4	3	3	3	0.13	0.31
GARS_MOUSE	Glycine–tRNA ligase	233	81826	6.24	15	8	12	6	12	0.33	0.36
AP1B1_MOUSE	AP-1 complex subunit beta-1	231	103869	5.04	17	11	13	8	9	0.26	0.44
NP1L1_MOUSE	Nucleosome assembly protein 1-like 1	231	45317	4.36	19	10	12	6	3	0.5	0.9
MTPN_MOUSE	Myotrophin	227	12853	5.27	12	7	6	4	6	0.68	2.55
GRN_MOUSE	Granulins	227	63413	6.42	21	15	5	4	5	0.14	0.3
CTNA1_MOUSE	Catenin alpha-1	226	100044	5.91	14	10	9	7	9	0.19	0.34
ECHM_MOUSE	Enoyl-CoA hydratase, mitochondrial	226	31454	8.76	10	9	6	5	6	0.29	1.21
ITB2_MOUSE	Integrin beta-2	226	84970	7.02	15	8	8	5	8	0.17	0.28
LKHA4_MOUSE	Leukotriene A-4 hydrolase	226	69007	5.98	13	10	7	6	7	0.21	0.44
MYADM_MOUSE	Myeloid-associated differentiation marker	225	35261	8.69	18	10	4	3	4	0.19	0.42
ARC1B_MOUSE	Actin-related protein 2/3 complex subunit 1B	224	41037	8.69	12	9	8	6	8	0.38	0.84
COPD_MOUSE	Coatomer subunit delta	222	57193	5.89	28	14	12	6	12	0.33	0.55
DUS3_MOUSE	Dual specificity protein phosphatase 3	222	20459	6.07	6	4	5	4	5	0.46	1.24
SYEP_MOUSE	Bifunctional glutamate/proline–tRNA ligase	222	169972	7.75	29	11	21	8	21	0.24	0.22
SC23A_MOUSE	Protein transport protein Sec23A	221	86106	6.64	28	14	18	8	17	0.46	0.55
NDUS1_MOUSE	NADH-ubiquinone oxidoreductase 75 kDa subunit, mitochondrial	220	79726	5.51	12	6	9	5	9	0.22	0.37
DDX1_MOUSE	ATP-dependent RNA helicase DDX1	220	82448	6.8	16	10	9	4	9	0.16	0.22
SYVC_MOUSE	Valine–tRNA ligase	220	140127	7.9	26	20	15	13	15	0.24	0.52
RS3A_MOUSE	40S ribosomal protein S3a	219	29866	9.75	31	11	17	9	17	0.55	2.49
PLOD3_MOUSE	Procollagen-lysine,2-oxoglutarate 5-dioxygenase 3	216	84869	5.81	23	14	14	8	14	0.32	0.48
F10A1_MOUSE	Hsc70-interacting protein	216	41630	5.19	10	7	4	3	4	0.15	0.35
LYZ2_MOUSE	Lysozyme C-2	214	16678	9.11	15	6	8	4	8	0.63	3.39
MIF_MOUSE	Macrophage migration inhibitory factor	214	12496	6.79	12	8	6	4	6	0.6	4.07
DEST_MOUSE	Destrin	214	18509	8.14	13	10	10	7	10	0.62	4.9
HCD2_MOUSE	3-Hydroxyacyl-CoA dehydrogenase type-2	214	27402	8.53	11	7	6	4	6	0.39	0.83
DNJA2_MOUSE	DnaJ homolog subfamily A member 2	213	45717	6.06	7	7	3	3	3	0.18	0.31
MARCS_MOUSE	Myristoylated alanine-rich C-kinase substrate	213	29644	4.34	13	9	3	3	3	0.27	0.52
LICH_MOUSE	Lysosomal acid lipase/cholesteryl ester hydrolase	212	45296	8.16	11	10	6	6	6	0.29	0.9
SAE2_MOUSE	SUMO-activating enzyme subunit 2	211	70525	5.09	9	8	5	4	5	0.2	0.27
RL6_MOUSE	60S ribosomal protein L6	210	33489	10.69	17	8	10	5	10	0.37	0.86
FKB10_MOUSE	Peptidyl-prolyl cis-trans isomerase FKBP10	208	64656	5.38	21	8	12	6	12	0.3	0.47
IPO4_MOUSE	Importin-4	208	119198	4.92	15	7	12	6	12	0.19	0.23
RL14_MOUSE	60S ribosomal protein L14	206	23549	11.03	12	6	6	2	6	0.33	0.42
PLD3_MOUSE	Phospholipase D3	205	54354	6.07	15	10	9	7	9	0.37	0.85
LRC59_MOUSE	Leucine-rich repeat-containing protein 59	205	34856	9.57	15	5	9	3	9	0.35	0.43
NIBAN_MOUSE	Protein Niban	204	102585	4.72	15	10	9	6	9	0.17	0.28
CATK_MOUSE	Cathepsin K	203	36865	8.61	5	4	4	3	4	0.25	0.4
HMCS1_MOUSE	Hydroxymethylglutaryl-CoA synthase, cytoplasmic	201	57532	5.65	17	10	12	6	12	0.39	0.66
GANAB_MOUSE	Neutral alpha-glucosidase AB	201	106844	5.67	16	11	12	9	12	0.24	0.42
PSB5_MOUSE	Proteasome subunit beta type-5	200	28514	6.52	10	7	7	4	7	0.36	0.79
LEG3_MOUSE	Galectin-3	200	27498	8.46	27	10	14	6	14	0.42	1.47
SYNC_MOUSE	Asparagine–tRNA ligase, cytoplasmic	197	64238	5.62	19	11	15	9	15	0.41	0.79
DCTN2_MOUSE	Dynactin subunit 2	197	44090	5.14	8	5	8	5	8	0.34	0.6
ZYX_MOUSE	Zyxin	197	60507	5.99	15	7	7	3	7	0.22	0.23
LTOR3_MOUSE	Ragulator complex protein LAMTOR3	195	13544	6.73	7	7	4	4	4	0.63	2.34
S10AB_MOUSE	Protein S100-A11	195	11075	5.28	8	6	4	3	4	0.77	3.32
VATL_MOUSE	V-type proton ATPase 16 kDa proteolipid subunit	195	15798	9.1	4	3	2	1	2	0.32	0.68
VMA5A_MOUSE	von Willebrand factor A domain-containing protein 5A	194	87087	6.15	10	5	9	4	9	0.23	0.21
IBP7_MOUSE	Insulin-like growth factor-binding protein 7	194	28951	8.71	16	7	8	3	8	0.46	0.54
EIF3H_MOUSE	Eukaryotic translation initiation factor 3 subunit H	192	39807	6.2	8	7	6	5	6	0.34	0.68
FHL2_MOUSE	Four and a half LIM domains protein 2	192	32051	7.31	14	6	6	3	6	0.28	0.47
PRS6B_MOUSE	26S proteasome regulatory subunit 6B	192	47379	5.09	9	5	8	4	8	0.4	0.42
ETFA_MOUSE	Electron transfer flavoprotein subunit alpha, mitochondrial	190	34988	8.62	17	8	8	5	7	0.47	0.81
CSF1_MOUSE	Macrophage colony-stimulating factor 1	190	60611	5.1	9	7	4	3	4	0.13	0.32
P4HA2_MOUSE	Prolyl 4-hydroxylase subunit alpha-2	189	60964	5.55	24	10	12	9	12	0.35	0.85
LAMB1_MOUSE	Laminin subunit beta-1	188	196961	4.82	17	5	12	3	12	0.12	0.07
TCPG_MOUSE	T-complex protein 1 subunit gamma	188	60591	6.28	25	8	17	6	17	0.42	0.51
SQSTM_MOUSE	Sequestosome-1	186	48132	5.09	10	7	6	4	6	0.28	0.41
DC1I2_MOUSE	Cytoplasmic dynein 1 intermediate chain 2	185	68352	5.16	5	4	4	3	4	0.19	0.2
CATA_MOUSE	Catalase	184	59758	7.72	15	6	11	4	11	0.35	0.32
VATC1_MOUSE	V-type proton ATPase subunit C 1	183	43860	7.02	8	5	5	3	5	0.25	0.33
ECHB_MOUSE	Trifunctional enzyme subunit beta, mitochondrial	183	51353	9.43	10	6	8	5	8	0.32	0.5
STIP1_MOUSE	Stress-induced phosphoprotein 1	183	62542	6.4	15	6	11	4	11	0.35	0.31
THY1_MOUSE	Thy-1 membrane glycoprotein	182	18069	9.16	14	8	5	2	5	0.49	0.58
PYRG1_MOUSE	CTP synthase 1	182	66640	6.14	17	10	11	7	11	0.33	0.65
GSTO1_MOUSE	Glutathione S-transferase omega-1	181	27480	6.92	36	11	19	7	19	0.83	1.87
HNRPM_MOUSE	Heterogeneous nuclear ribonucleoprotein M	180	77597	8.8	16	8	11	6	11	0.23	0.38
PCOC1_MOUSE	Procollagen C-endopeptidase enhancer 1	180	50136	8.73	14	6	9	4	9	0.38	0.39
ATPO_MOUSE	ATP synthase subunit O, mitochondrial	180	23349	10	10	7	6	5	6	0.38	1.43
IDH3A_MOUSE	Isocitrate dehydrogenase [NAD] subunit alpha, mitochondrial	179	39613	6.27	9	5	4	1	4	0.15	0.23
POSTN_MOUSE	Periostin	179	93085	7.27	15	7	10	4	10	0.25	0.2
HCDH_MOUSE	Hydroxyacyl-coenzyme A dehydrogenase, mitochondrial	179	34442	8.76	10	6	6	4	6	0.5	0.62
DHB4_MOUSE	Peroxisomal multifunctional enzyme type 2	179	79432	8.76	22	13	13	8	13	0.24	0.52
PTGIS_MOUSE	Prostacyclin synthase	178	57011	6.26	21	9	18	8	18	0.53	0.79
TPP1_MOUSE	Tripeptidyl-peptidase 1	177	61304	6.1	8	7	6	5	6	0.21	0.5
SAE1_MOUSE	SUMO-activating enzyme subunit 1	177	38596	5.24	7	5	5	3	5	0.24	0.38
6PGL_MOUSE	6-Phosphogluconolactonase	177	27237	5.55	9	6	5	2	5	0.35	0.36
SUCB1_MOUSE	Succinate–CoA ligase [ADP-forming] subunit beta, mitochondrial	176	50082	6.57	8	5	6	3	6	0.27	0.28
IDI1_MOUSE	Isopentenyl-diphosphate delta-isomerase 1	175	26272	5.79	14	5	6	4	6	0.49	0.88
PLIN3_MOUSE	Perilipin-3	175	47233	5.45	12	9	10	8	10	0.43	1.02
RL7_MOUSE	60S ribosomal protein L7	174	31400	10.89	19	9	13	5	13	0.46	0.93
RAB1B_MOUSE	Ras-related protein Rab-1B	130	22173	5.55	15	7	10	5	4	0.52	1.53
FERM2_MOUSE	Fermitin family homolog 2	174	77750	6.26	9	4	6	3	6	0.18	0.18
CAZA2_MOUSE	F-actin-capping protein subunit alpha-2	173	32947	5.57	19	10	11	7	4	0.65	1.41
CAZA1_MOUSE	F-actin-capping protein subunit alpha-1	61	32919	5.34	9	3	7	3	5	0.44	0.46
EIF3K_MOUSE	Eukaryotic translation initiation factor 3 subunit K	173	25070	4.81	10	3	8	2	8	0.61	0.39
G6PD1_MOUSE	Glucose-6-phosphate 1-dehydrogenase X	172	59225	6.06	22	11	14	7	14	0.4	0.64
ASAH1_MOUSE	Acid ceramidase	172	44641	8.68	10	7	9	6	9	0.3	0.75
ADHX_MOUSE	Alcohol dehydrogenase class-3	172	39522	6.97	14	7	9	7	9	0.51	1.09
IF4G1_MOUSE	Eukaryotic translation initiation factor 4 gamma 1	172	175967	5.3	16	10	11	7	11	0.11	0.18
IL6RB_MOUSE	Interleukin-6 receptor subunit beta	171	102387	5.35	14	8	4	2	4	0.06	0.09
NMT1_MOUSE	Glycylpeptide N-tetradecanoyltransferase 1	171	56852	8.04	8	5	6	3	6	0.24	0.25
ANXA4_MOUSE	Annexin A4	170	35893	5.43	10	7	8	5	8	0.3	0.78
PCYOX_MOUSE	Prenylcysteine oxidase	169	56459	6.44	8	5	7	4	7	0.24	0.34
BASP1_MOUSE	Brain acid soluble protein 1	168	22074	4.5	21	9	5	3	5	0.42	0.75
PSMD5_MOUSE	26S proteasome non-ATPase regulatory subunit 5	168	55937	5.13	10	5	7	3	7	0.28	0.25
IPYR_MOUSE	Inorganic pyrophosphatase	165	32646	5.37	6	4	4	2	4	0.26	0.29
ISG15_MOUSE	Ubiquitin-like protein ISG15	164	17886	7.74	6	5	3	2	3	0.5	0.58
ACO13_MOUSE	Acyl-coenzyme A thioesterase 13	164	15173	8.95	8	7	5	5	5	0.56	2.84
CP51A_MOUSE	Lanosterol 14-alpha demethylase	164	56739	8.6	12	4	8	3	5	0.3	0.25
IMPA1_MOUSE	Inositol monophosphatase 1	163	30416	5.08	13	4	5	2	5	0.21	0.31
P3H1_MOUSE	Prolyl 3-hydroxylase 1	162	83598	5.03	12	6	8	4	8	0.22	0.22
SDHA_MOUSE	Succinate dehydrogenase [ubiquinone] flavoprotein subunit, mitochondrial	161	72539	7.06	12	9	5	4	5	0.15	0.26
VPS25_MOUSE	Vacuolar protein-sorting-associated protein 25	161	20735	5.97	4	4	1	1	1	0.19	0.49
PHB_MOUSE	Prohibitin	159	29802	5.57	17	10	11	7	11	0.65	1.65
PSA6_MOUSE	Proteasome subunit alpha type-6	158	27355	6.34	14	5	8	3	8	0.52	0.58
TOM40_MOUSE	Mitochondrial import receptor subunit TOM40 homolog	156	37871	7.64	10	8	6	4	6	0.32	0.73
UBP5_MOUSE	Ubiquitin carboxyl-terminal hydrolase 5	156	95772	4.89	18	10	10	7	10	0.23	0.36
EFTU_MOUSE	Elongation factor Tu, mitochondrial	156	49477	7.23	16	5	12	3	11	0.44	0.29
DHB7_MOUSE	3-Keto-steroid reductase	154	37293	6.25	9	5	6	4	6	0.26	0.75
CAVN1_MOUSE	Caveolae-associated protein 1	153	43927	5.43	7	6	3	2	3	0.15	0.21
ODPB_MOUSE	Pyruvate dehydrogenase E1 component subunit beta, mitochondrial	152	38912	6.41	14	7	6	4	6	0.32	0.53
DNPEP_MOUSE	Aspartyl aminopeptidase	152	52174	6.82	18	12	10	7	10	0.41	0.75
PRDX5_MOUSE	Peroxiredoxin-5, mitochondrial	151	21884	9.1	20	5	10	4	10	0.54	1.13
ARPC2_MOUSE	Actin-related protein 2/3 complex subunit 2	150	34336	6.84	13	4	10	2	10	0.39	0.27
PCKGM_MOUSE	Phosphoenolpyruvate carboxykinase [GTP], mitochondrial	150	70482	6.92	4	3	3	2	3	0.08	0.13
GLCM_MOUSE	Glucosylceramidase	150	57585	7.64	14	10	10	8	10	0.37	0.78
NB5R1_MOUSE	NADH-cytochrome b5 reductase 1	150	34113	8.97	4	3	4	3	4	0.26	0.44
PUR6_MOUSE	Multifunctional protein ADE2	149	46976	6.94	11	6	8	5	8	0.37	0.56
LPPRC_MOUSE	Leucine-rich PPR motif-containing protein, mitochondrial	149	156516	6.42	18	4	14	4	14	0.16	0.11
SAC1_MOUSE	Phosphatidylinositide phosphatase SAC1	148	66901	6.85	9	4	4	2	4	0.12	0.13
GFPT2_MOUSE	Glutamine–fructose-6-phosphate aminotransferase [isomerizing] 2	147	76960	6.72	10	4	9	4	9	0.21	0.24
MTAP_MOUSE	S-Methyl-5′-thioadenosine phosphorylase	146	31042	6.71	7	4	5	3	5	0.29	0.49
IKIP_MOUSE	Inhibitor of nuclear factor kappa-B kinase-interacting protein	146	42505	5.03	13	10	4	2	4	0.29	0.22
ADPGK_MOUSE	ADP-dependent glucokinase	146	53869	5.37	9	7	4	3	4	0.15	0.26
MA2A1_MOUSE	Alpha-mannosidase 2	145	131548	8.17	12	7	10	6	10	0.15	0.21
SYLC_MOUSE	Leucine–tRNA ligase, cytoplasmic	145	134106	6.64	13	6	10	4	10	0.16	0.13
SYIC_MOUSE	Isoleucine–tRNA ligase, cytoplasmic	145	144179	6.14	18	8	12	5	12	0.19	0.16
PARVA_MOUSE	Alpha-parvin	144	42304	5.69	9	5	5	3	5	0.26	0.34
DYN2_MOUSE	Dynamin-2	144	98084	7.02	12	5	9	3	7	0.19	0.14
IMA1_MOUSE	Importin subunit alpha-1	144	57892	5.49	13	4	8	3	8	0.27	0.24
CYGB_MOUSE	Cytoglobin	144	21452	6.32	8	4	3	2	3	0.33	0.47
ODO1_MOUSE	2-Oxoglutarate dehydrogenase, mitochondrial	143	116375	6.36	34	6	9	4	9	0.14	0.15
MK01_MOUSE	Mitogen-activated protein kinase 1	143	41249	6.5	8	5	7	4	7	0.38	0.5
UD17C_MOUSE	UDP-glucuronosyltransferase 1-7C	143	59719	8.64	14	5	8	3	8	0.27	0.23
HMOX1_MOUSE	Heme oxygenase 1	142	32908	6.08	16	7	10	6	10	0.57	1.13
USO1_MOUSE	General vesicular transport factor p115	142	106917	4.85	11	7	10	6	10	0.17	0.26
RTN4_MOUSE	Reticulon-4	142	126535	4.47	23	9	10	5	10	0.16	0.18
THIKA_MOUSE	3-Ketoacyl-CoA thiolase A, peroxisomal	141	43926	8.74	14	8	10	5	10	0.47	0.61
MGST1_MOUSE	Microsomal glutathione S-transferase 1	141	17540	9.67	6	5	2	2	2	0.22	0.6
HYEP_MOUSE	Epoxide hydrolase 1	141	52543	8.43	18	5	14	4	14	0.41	0.37
GUAD_MOUSE	Guanine deaminase	141	50981	5.36	6	5	4	3	4	0.24	0.28
LASP1_MOUSE	LIM and SH3 domain protein 1	140	29975	6.61	23	7	7	2	7	0.29	0.51
4F2_MOUSE	4F2 cell-surface antigen heavy chain	139	58300	5.62	12	8	7	5	7	0.26	0.43
PPAC_MOUSE	Low molecular weight phosphotyrosine protein phosphatase	139	18180	6.3	6	3	4	3	4	0.45	0.97
CD36_MOUSE	Platelet glycoprotein 4	139	52664	8.6	6	5	6	5	6	0.19	0.49
ATPG_MOUSE	ATP synthase subunit gamma, mitochondrial	139	32865	9.06	9	6	6	3	6	0.3	0.46
AL9A1_MOUSE	4-Trimethylaminobutyraldehyde dehydrogenase	138	53480	6.63	7	6	4	3	4	0.18	0.26
ASPH_MOUSE	Aspartyl/asparaginyl beta-hydroxylase	138	82991	4.97	6	4	5	3	5	0.1	0.16
ATPD_MOUSE	ATP synthase subunit delta, mitochondrial	138	17589	5.03	14	9	5	2	5	0.63	0.6
IRGM1_MOUSE	Immunity-related GTPase family M protein 1	137	46522	8.56	7	5	4	4	4	0.2	0.43
PON3_MOUSE	Serum paraoxonase/lactonase 3	137	39326	5.44	9	5	5	4	5	0.26	0.53
UMPS_MOUSE	Uridine 5′-monophosphate synthase	137	52259	6.17	7	5	6	4	6	0.23	0.38
CMTD1_MOUSE	Catechol O-methyltransferase domain-containing protein 1	137	28943	8.61	7	4	6	3	6	0.51	0.54
SYUG_MOUSE	Gamma-synuclein	137	13152	4.68	6	5	3	3	3	0.4	1.54
CLIC4_MOUSE	Chloride intracellular channel protein 4	137	28711	5.44	10	7	8	5	7	0.41	1.06
CRIP2_MOUSE	Cysteine-rich protein 2	136	22712	8.94	8	5	3	3	3	0.34	1.07
PLOD1_MOUSE	Procollagen-lysine,2-oxoglutarate 5-dioxygenase 1	136	83542	6.08	12	5	10	4	10	0.2	0.22
CPNE1_MOUSE	Copine-1	136	58849	5.4	7	4	5	3	5	0.18	0.24
RL4_MOUSE	60S ribosomal protein L4	136	47124	11.01	40	10	20	9	20	0.45	1.21
RN213_MOUSE	E3 ubiquitin-protein ligase RNF213	135	584411	6.35	29	8	22	6	22	0.09	0.04
CD109_MOUSE	CD109 antigen	134	161557	5.37	5	5	4	4	4	0.05	0.11
LAMP2_MOUSE	Lysosome-associated membrane glycoprotein 2	134	45652	7.05	11	4	7	3	7	0.16	0.31
ARL8A_MOUSE	ADP-ribosylation factor-like protein 8A	134	21376	7.63	14	8	6	4	6	0.4	1.16
NCEH1_MOUSE	Neutral cholesterol ester hydrolase 1	133	45711	6.56	12	7	7	4	7	0.34	0.44
GNPI1_MOUSE	Glucosamine-6-phosphate isomerase 1	133	32528	6.13	8	5	7	4	7	0.36	0.67
NOMO1_MOUSE	Nodal modulator 1	133	133336	5.75	13	8	10	7	10	0.19	0.25
RRAS_MOUSE	Ras-related protein R-Ras	133	23749	6.32	6	4	4	3	2	0.23	0.68
RCN1_MOUSE	Reticulocalbin-1	133	38090	4.7	12	6	6	4	6	0.25	0.55
PFKAP_MOUSE	ATP-dependent 6-phosphofructokinase, platelet type	132	85400	6.73	12	4	9	2	9	0.24	0.1
BLVRB_MOUSE	Flavin reductase (NADPH)	132	22183	6.49	15	6	9	5	9	0.69	1.53
TMM43_MOUSE	Transmembrane protein 43	132	44755	6.85	6	5	6	5	6	0.31	0.59
P5CS_MOUSE	Delta-1-pyrroline-5-carboxylate synthase	131	87212	7.18	12	5	9	4	9	0.19	0.21
MATR3_MOUSE	Matrin-3	131	94572	5.87	6	4	5	3	5	0.14	0.14
PTGR1_MOUSE	Prostaglandin reductase 1	131	35537	8.09	5	5	5	5	5	0.26	0.79
COTL1_MOUSE	Coactosin-like protein	131	15934	5.28	10	3	6	2	6	0.55	0.67
ACAD9_MOUSE	Acyl-CoA dehydrogenase family member 9, mitochondrial	131	68679	7.16	6	6	3	3	3	0.11	0.2
IFM3_MOUSE	Interferon-induced transmembrane protein 3	130	14945	6.89	14	6	3	3	2	0.32	1.98
IFM2_MOUSE	Interferon-induced transmembrane protein 2	59	15733	6.81	9	3	3	2	2	0.17	0.68
DHX9_MOUSE	ATP-dependent RNA helicase A	130	149381	6.39	17	9	11	5	11	0.13	0.15
SDCB1_MOUSE	Syntenin-1	130	32359	6.66	9	4	4	3	4	0.29	0.47
ORN_MOUSE	Oligoribonuclease, mitochondrial	129	26722	6.67	8	4	6	3	6	0.38	0.59
ITM2B_MOUSE	Integral membrane protein 2B	128	30240	5.14	16	7	7	5	7	0.45	0.99
EIF3I_MOUSE	Eukaryotic translation initiation factor 3 subunit I	128	36438	5.38	15	6	9	5	9	0.47	0.77
FIS1_MOUSE	Mitochondrial fission 1 protein	128	16998	8.56	5	4	4	3	4	0.31	1.07
SAHH_MOUSE	Adenosylhomocysteinase	128	47657	6.08	15	8	11	7	11	0.43	0.84
TFR1_MOUSE	Transferrin receptor protein 1	127	85677	6.13	4	4	3	3	3	0.05	0.16
STML2_MOUSE	Stomatin-like protein 2, mitochondrial	127	38361	8.95	13	8	9	6	9	0.52	0.91
IF6_MOUSE	Eukaryotic translation initiation factor 6	127	26494	4.63	7	6	5	4	5	0.56	1.18
VPP1_MOUSE	V-type proton ATPase 116 kDa subunit a isoform 1	126	96404	6.29	10	4	7	3	7	0.13	0.14
PREP_MOUSE	Presequence protease, mitochondrial	126	117297	6.76	10	6	10	6	10	0.17	0.24
BIN1_MOUSE	Myc box-dependent-interacting protein 1	125	64430	4.95	16	6	10	5	10	0.32	0.38
CSN7A_MOUSE	COP9 signalosome complex subunit 7a	125	30206	7.68	8	6	4	3	4	0.26	0.51
SSRD_MOUSE	Translocon-associated protein subunit delta	125	18924	5.5	7	6	3	2	3	0.25	0.55
COR1B_MOUSE	Coronin-1B	124	53878	5.54	15	7	7	3	7	0.29	0.26
VKOR1_MOUSE	Vitamin K epoxide reductase complex subunit 1	124	17756	9.37	6	4	2	2	2	0.19	0.59
FUBP2_MOUSE	Far upstream element-binding protein 2	124	76728	6.9	11	4	7	3	7	0.12	0.18
MFGM_MOUSE	Lactadherin	122	51208	6.1	22	9	13	7	13	0.45	0.77
PSME2_MOUSE	Proteasome activator complex subunit 2	122	27040	5.54	10	6	7	4	7	0.46	0.84
C1TC_MOUSE	C-1-tetrahydrofolate synthase, cytoplasmic	122	101136	6.7	12	5	8	3	8	0.12	0.13
RISC_MOUSE	Retinoid-inducible serine carboxypeptidase	122	50932	5.48	11	5	8	4	8	0.21	0.39
ETFB_MOUSE	Electron transfer flavoprotein subunit beta	122	27606	8.24	16	4	8	2	8	0.43	0.35
PRRC1_MOUSE	Protein PRRC1	122	46268	5.62	10	5	8	5	8	0.31	0.57
MAOM_MOUSE	NAD-dependent malic enzyme, mitochondrial	121	65757	7.53	6	5	3	3	3	0.15	0.21
SH3L1_MOUSE	SH3 domain-binding glutamic acid-rich-like protein	120	12803	4.87	4	3	3	2	3	0.38	0.89
PSB1_MOUSE	Proteasome subunit beta type-1	120	26355	7.67	9	5	7	4	7	0.55	0.88
LY6E_MOUSE	Lymphocyte antigen 6E	120	13791	6.67	4	3	2	2	2	0.29	1.44
SPRC_MOUSE	SPARC	120	34428	4.77	8	5	6	4	6	0.33	0.62
ECI2_MOUSE	Enoyl-CoA delta isomerase 2, mitochondrial	119	43240	9.08	9	5	4	1	4	0.16	0.1
AIMP2_MOUSE	Aminoacyl tRNA synthase complex-interacting multifunctional protein 2	119	35355	7.7	9	6	5	4	5	0.34	0.6
TOM1_MOUSE	Target of Myb protein 1	119	54291	4.83	5	4	5	4	5	0.22	0.36
TALDO_MOUSE	Transaldolase	119	37363	6.57	16	5	10	4	10	0.31	0.56
SAMH1_MOUSE	Deoxynucleoside triphosphate triphosphohydrolase SAMHD1	118	72604	8.17	8	4	6	2	6	0.17	0.12
LPP_MOUSE	Lipoma-preferred partner homolog	118	65848	7.19	11	7	6	4	6	0.22	0.29
STX12_MOUSE	Syntaxin-12	118	31176	5.33	9	5	8	4	8	0.45	0.7
CYB5B_MOUSE	Cytochrome b5 type B	118	16308	4.79	5	4	4	3	4	0.51	1.13
PDLI1_MOUSE	PDZ and LIM domain protein 1	117	35752	6.38	9	4	7	4	7	0.38	0.59
FABP4_MOUSE	Fatty acid-binding protein, adipocyte	117	14641	8.53	6	3	5	3	5	0.47	1.31
BACH_MOUSE	Cytosolic acyl coenzyme A thioester hydrolase	117	42510	8.9	6	3	5	3	5	0.24	0.34
NSF1C_MOUSE	NSFL1 cofactor p47	117	40685	5.04	9	7	2	2	2	0.11	0.23
CMC1_MOUSE	Calcium-binding mitochondrial carrier protein Aralar1	117	74523	8.43	8	2	7	2	7	0.18	0.12
S10A1_MOUSE	Protein S100-A1	117	10498	4.37	2	2	1	1	1	0.16	0.47
ACDSB_MOUSE	Short/branched chain specific acyl-CoA dehydrogenase, mitochondrial	116	47843	8	7	4	4	2	4	0.19	0.19
CO4A1_MOUSE	Collagen alpha-1(IV) chain	116	160579	8.51	16	3	8	2	8	0.13	0.08
SPEE_MOUSE	Spermidine synthase	116	33973	5.31	10	4	10	4	10	0.65	0.63
EDC4_MOUSE	Enhancer of mRNA-decapping protein 4	115	152389	5.51	6	4	3	2	3	0.03	0.06
SGT1_MOUSE	Protein SGT1 homolog	115	38135	5.32	4	3	3	2	3	0.16	0.24
SQOR_MOUSE	Sulfide:quinone oxidoreductase, mitochondrial	115	50250	9.2	6	4	4	2	4	0.12	0.18
SYWC_MOUSE	Tryptophan–tRNA ligase, cytoplasmic	114	54323	6.44	9	5	7	4	7	0.26	0.36
PDXD1_MOUSE	Pyridoxal-dependent decarboxylase domain-containing protein 1	114	87281	5.31	12	6	7	4	7	0.18	0.21
BROX_MOUSE	BRO1 domain-containing protein BROX	114	46172	7.59	6	4	2	2	2	0.11	0.2
SCFD1_MOUSE	Sec1 family domain-containing protein 1	112	72277	5.98	5	2	5	2	5	0.19	0.12
DHPR_MOUSE	Dihydropteridine reductase	113	25554	7.67	4	2	2	1	2	0.11	0.18
NDUS3_MOUSE	NADH dehydrogenase [ubiquinone] iron-sulfur protein 3, mitochondrial	113	30131	6.67	4	3	2	1	2	0.11	0.15
TAGL_MOUSE	Transgelin	113	22561	8.85	12	6	8	5	8	0.51	2
PALLD_MOUSE	Palladin	113	152037	5.87	11	4	9	3	9	0.11	0.09
RL18_MOUSE	60S ribosomal protein L18	112	21631	11.79	7	6	4	3	4	0.25	0.77
MPCP_MOUSE	Phosphate carrier protein, mitochondrial	112	39606	9.36	39	5	9	3	9	0.28	0.37
CYFP1_MOUSE	Cytoplasmic FMR1-interacting protein 1	112	145148	6.46	6	3	6	3	6	0.07	0.09
G3BP1_MOUSE	Ras GTPase-activating protein-binding protein 1	112	51797	5.41	6	4	6	4	6	0.17	0.38
PYGB_MOUSE	Glycogen phosphorylase, brain form	112	96668	6.28	8	3	7	2	7	0.15	0.09
S10A6_MOUSE	Protein S100-A6	112	10044	5.3	12	5	6	3	6	0.66	4.05
TCTP_MOUSE	Translationally-controlled tumor protein	110	19450	4.76	9	4	5	3	5	0.31	0.89
RL5_MOUSE	60S ribosomal protein L5	110	34379	9.78	12	4	9	3	9	0.43	0.44
SYHC_MOUSE	Histidine–tRNA ligase, cytoplasmic	110	57396	5.79	10	5	6	4	6	0.22	0.34
CLPT1_MOUSE	Cleft lip and palate transmembrane protein 1 homolog	109	75243	5.88	13	9	5	4	5	0.18	0.25
ACSF2_MOUSE	Acyl-CoA synthetase family member 2, mitochondrial	109	67907	8.44	7	4	6	3	6	0.2	0.2
THIL_MOUSE	Acetyl-CoA acetyltransferase, mitochondrial	108	44787	8.71	15	9	6	4	6	0.23	0.45
USMG5_MOUSE	Upregulated during skeletal muscle growth protein 5	108	6377	9.84	2	2	1	1	1	0.26	0.87
ISC2A_MOUSE	Isochorismatase domain-containing protein 2A	108	22403	8.25	2	2	1	1	1	0.09	0.2
HNRPL_MOUSE	Heterogeneous nuclear ribonucleoprotein L	107	63923	8.33	14	6	6	2	6	0.24	0.14
PP1R7_MOUSE	Protein phosphatase 1 regulatory subunit 7	106	41266	4.85	3	3	2	2	2	0.11	0.22
SE1L1_MOUSE	Protein sel-1 homolog 1	106	88285	5.36	4	3	3	2	3	0.1	0.1
CCD47_MOUSE	Coiled-coil domain-containing protein 47	106	55808	4.74	6	5	3	2	1	0.14	0.16
HUWE1_MOUSE	E3 ubiquitin-protein ligase HUWE1	105	482332	5.1	15	2	13	1	13	0.06	0.01
GDIR2_MOUSE	Rho GDP-dissociation inhibitor 2	104	22836	4.97	5	4	4	3	4	0.45	0.72
SGPL1_MOUSE	Sphingosine-1-phosphate lyase 1	104	63636	9.2	12	5	8	4	8	0.29	0.3
PLIN4_MOUSE	Perilipin-4	104	139328	8.81	7	4	6	3	6	0.07	0.09
UBA6_MOUSE	Ubiquitin-like modifier-activating enzyme 6	104	117891	5.75	6	2	5	1	5	0.07	0.04
ARL1_MOUSE	ADP-ribosylation factor-like protein 1	104	20398	5.63	8	5	6	3	6	0.57	0.83
G6PE_MOUSE	GDH/6PGL endoplasmic bifunctional protein	103	88872	6.44	6	4	5	3	5	0.11	0.15
UBP14_MOUSE	Ubiquitin carboxyl-terminal hydrolase 14	103	55966	5.15	8	4	7	3	7	0.25	0.25
FSTL1_MOUSE	Follistatin-related protein 1	103	34532	5.58	6	2	5	1	5	0.21	0.13
PGFRB_MOUSE	Platelet-derived growth factor receptor beta	103	122728	4.99	10	7	7	4	7	0.12	0.15
CD180_MOUSE	CD180 antigen	103	74255	5.55	4	2	3	1	3	0.12	0.06
GLRX1_MOUSE	Glutaredoxin-1	103	11863	8.67	4	3	2	1	2	0.49	0.41
GCN1_MOUSE	eIF-2-alpha kinase activator GCN1	102	292834	7.14	18	5	16	3	16	0.12	0.04
PLPP3_MOUSE	Phospholipid phosphatase 3	102	35193	9.25	6	4	4	2	4	0.23	0.27
NU155_MOUSE	Nuclear pore complex protein Nup155	101	155019	5.77	9	4	7	2	7	0.09	0.06
ACADV_MOUSE	Very-long-chain-specific acyl-CoA dehydrogenase, mitochondrial	95	70831	8.91	3	2	3	2	3	0.08	0.13
PFD5_MOUSE	Prefoldin subunit 5	101	17345	5.93	8	2	5	2	5	0.43	0.61
PSA2_MOUSE	Proteasome subunit alpha type-2	101	25910	6.92	9	5	8	5	8	0.52	1.22
ZWINT_MOUSE	ZW10 interactor	101	28695	8.56	4	2	3	1	3	0.24	0.33
ERG7_MOUSE	Lanosterol synthase	101	83088	5.96	13	4	10	4	10	0.21	0.22
EMC1_MOUSE	ER membrane protein complex subunit 1	100	111535	7	12	6	11	5	11	0.19	0.21
PYC_MOUSE	Pyruvate carboxylase, mitochondrial	100	129602	6.25	4	2	4	2	4	0.07	0.07
SRPRB_MOUSE	Signal recognition particle receptor subunit beta	99	29561	9.34	7	5	4	2	4	0.26	0.32
CD34_MOUSE	Hematopoietic progenitor cell antigen CD34	99	40957	5.2	1	1	1	1	1	0.05	0.11
AIFM1_MOUSE	Apoptosis-inducing factor 1, mitochondrial	98	66724	9.23	5	3	4	3	4	0.14	0.21
3HIDH_MOUSE	3-Hydroxyisobutyrate dehydrogenase, mitochondrial	98	35417	8.37	6	3	5	2	5	0.31	0.26
ATLA3_MOUSE	Atlastin-3	98	60537	5.73	7	5	5	4	5	0.18	0.32
TXTP_MOUSE	Tricarboxylate transport protein, mitochondrial	98	33910	9.91	6	2	4	2	4	0.27	0.28
IF1A_MOUSE	Eukaryotic translation initiation factor 1A	98	16492	5.07	8	4	3	1	3	0.21	0.28
TRI25_MOUSE	E3 ubiquitin/ISG15 ligase TRIM25	98	71680	8.62	9	2	7	2	7	0.21	0.12
QCR1_MOUSE	Cytochrome b-c1 complex subunit 1, mitochondrial	97	52818	5.81	11	3	6	2	6	0.26	0.17
ACACA_MOUSE	Acetyl-CoA carboxylase 1	96	265088	5.97	11	6	10	5	10	0.11	0.08
DYLT1_MOUSE	Dynein light chain Tctex-type 1	95	12475	5	1	1	1	1	1	0.16	0.39
ATOX1_MOUSE	Copper transport protein ATOX1	95	7334	6.04	5	3	3	2	3	0.72	4.12
RENBP_MOUSE	N-Acylglucosamine 2-epimerase	94	49739	5.69	4	3	3	2	3	0.13	0.18
ROAA_MOUSE	Heterogeneous nuclear ribonucleoprotein A/B	94	30812	7.68	15	6	7	5	6	0.29	0.96
GNS_MOUSE	N-Acetylglucosamine-6-sulfatase	94	61136	8.52	13	5	8	3	8	0.22	0.23
TMX1_MOUSE	Thioredoxin-related transmembrane protein 1	93	31376	5.19	3	3	1	1	1	0.04	0.14
OCAD1_MOUSE	OCIA domain-containing protein 1	90	27593	7.66	4	3	3	2	3	0.2	0.35
STS_MOUSE	Steryl-sulfatase	93	66549	8.83	2	2	1	1	1	0.03	0.06
TIF1B_MOUSE	Transcription intermediary factor 1-beta	93	88791	5.52	11	4	7	4	7	0.12	0.21
LMAN2_MOUSE	Vesicular integral-membrane protein VIP36	92	40404	6.46	10	4	8	3	8	0.32	0.36
SH3L3_MOUSE	SH3 domain-binding glutamic acid-rich-like protein 3	92	10470	5.02	10	6	4	2	4	0.68	3.72
PTGR3_MOUSE	Prostaglandin reductase-3	92	40503	7.01	4	3	4	3	4	0.25	0.36
KIME_MOUSE	Mevalonate kinase	91	41851	6.22	5	2	5	2	5	0.3	0.22
PAPS2_MOUSE	Bifunctional 3′-phosphoadenosine 5′-phosphosulfate synthase 2	91	70306	7.31	8	3	7	3	7	0.24	0.2
PGRC1_MOUSE	Membrane-associated progesterone receptor component 1	91	21681	4.57	8	5	4	3	4	0.4	0.77
FRIH_MOUSE	Ferritin heavy chain	90	21053	5.53	16	6	9	4	9	0.73	1.19
THIM_MOUSE	3-Ketoacyl-CoA thiolase, mitochondrial	90	41803	8.33	13	8	8	4	8	0.34	0.49
LGMN_MOUSE	Legumain	90	49341	5.92	6	3	5	2	5	0.22	0.18
CD9_MOUSE	CD9 antigen	90	25241	6.88	4	3	1	1	1	0.11	0.39
HEBP1_MOUSE	Heme-binding protein 1	90	21053	5.18	2	1	2	1	2	0.29	0.22
SCPDL_MOUSE	Saccharopine dehydrogenase-like oxidoreductase	90	47099	8.86	4	4	2	2	2	0.13	0.19
TOIP1_MOUSE	Torsin-1A-interacting protein 1	90	66740	6.58	2	1	2	1	2	0.04	0.06
ACADL_MOUSE	Long-chain specific acyl-CoA dehydrogenase, mitochondrial	89	47877	8.53	15	5	8	4	8	0.35	0.42
PTGES_MOUSE	Prostaglandin E synthase	89	17274	9.47	2	2	1	1	1	0.17	0.27
PSA1_MOUSE	Proteasome subunit alpha type-1	89	29528	6	11	5	7	4	7	0.32	0.75
DX39A_MOUSE	ATP-dependent RNA helicase DDX39A	89	49036	5.46	5	2	5	2	5	0.23	0.19
ATP5H_MOUSE	ATP synthase subunit d, mitochondrial	89	18738	5.52	7	4	6	3	6	0.55	0.93
CNPY2_MOUSE	Protein canopy homolog 2	88	20754	4.95	8	5	3	2	3	0.28	0.49
AACS_MOUSE	Acetoacetyl-CoA synthetase	88	75152	6.25	15	5	11	5	11	0.28	0.32
GNAS1_MOUSE	Guanine nucleotide-binding protein G(s) subunit alpha isoforms Xlas	88	121429	4.72	15	5	8	3	7	0.11	0.11
HA11_MOUSE	H-2 class I histocompatibility antigen, D-B alpha chain	88	40810	6.28	13	2	8	2	8	0.29	0.23
GNA11_MOUSE	Guanine nucleotide-binding protein subunit alpha-11	88	41997	5.7	7	4	5	3	2	0.22	0.35
VATG1_MOUSE	V-type proton ATPase subunit G 1	88	13716	7.77	4	3	2	1	2	0.24	0.35
NDUS5_MOUSE	NADH dehydrogenase [ubiquinone] iron-sulfur protein 5	87	12639	9.1	1	1	1	1	1	0.11	0.38
SYFB_MOUSE	Phenylalanine–tRNA ligase beta subunit	87	65655	6.69	8	3	8	3	8	0.21	0.21
THOP1_MOUSE	Thimet oligopeptidase	87	77976	5.72	3	3	2	2	2	0.04	0.11
HSPB1_MOUSE	Heat shock protein beta-1	86	23000	6.12	9	2	7	2	7	0.56	0.43
IF2G_MOUSE	Eukaryotic translation initiation factor 2 subunit 3, X-linked	86	51033	8.66	15	5	10	5	10	0.3	0.5
PDIA5_MOUSE	Protein disulfide-isomerase A5	86	59229	7.25	6	2	5	1	5	0.22	0.15
BAG3_MOUSE	BAG family molecular chaperone regulator 3	86	61822	6.78	12	4	7	3	7	0.23	0.22
MRGRF_MOUSE	Mas-related G-protein coupled receptor member F	86	38497	9	2	2	1	1	1	0.06	0.11
SET_MOUSE	Protein SET	85	33358	4.22	10	3	7	3	7	0.37	0.45
GLTP_MOUSE	Glycolipid transfer protein	85	23674	6.9	2	2	2	2	2	0.2	0.42
DPP3_MOUSE	Dipeptidyl peptidase 3	85	82846	5.26	6	3	4	1	4	0.12	0.05
CARM1_MOUSE	Histone-arginine methyltransferase CARM1	85	65811	6.28	5	3	4	3	4	0.12	0.21
SERC1_MOUSE	Serine incorporator 1	85	50475	5.91	3	2	2	1	2	0.06	0.09
DCUP_MOUSE	Uroporphyrinogen decarboxylase	85	40666	6.21	4	3	3	2	3	0.21	0.23
KINH_MOUSE	Kinesin-1 heavy chain	85	109484	6.06	11	5	8	3	8	0.13	0.12
KANK2_MOUSE	KN motif and ankyrin repeat domain-containing protein 2	85	90190	5.38	6	2	3	1	3	0.06	0.05
AGM1_MOUSE	Phosphoacetylglucosamine mutase	84	59415	5.8	3	2	3	2	3	0.14	0.15
USP9X_MOUSE	Probable ubiquitin carboxyl-terminal hydrolase FAF-X	84	290526	5.57	8	1	5	1	5	0.04	0.01
ADDA_MOUSE	Alpha-adducin	84	80596	5.62	6	2	4	1	4	0.11	0.05
FA98B_MOUSE	Protein FAM98B	84	45321	8.77	3	2	3	2	3	0.15	0.2
GALK2_MOUSE	N-Acetylgalactosamine kinase	84	50470	6.47	10	4	6	3	6	0.38	0.39
UB2V1_MOUSE	Ubiquitin-conjugating enzyme E2 variant 1	84	16344	7.74	11	3	6	2	6	0.58	0.65
PPGB_MOUSE	Lysosomal protective protein	83	53809	5.56	9	3	8	3	8	0.27	0.26
EIF2A_MOUSE	Eukaryotic translation initiation factor 2A	83	64363	9.04	11	6	5	4	5	0.14	0.3
CATH_MOUSE	Pro-cathepsin H	83	37146	8.68	4	3	3	3	3	0.21	0.4
CD97_MOUSE	CD97 antigen	83	90354	7.38	2	2	1	1	1	0.03	0.05
RDH11_MOUSE	Retinol dehydrogenase 11	83	35125	9.1	10	6	5	3	5	0.24	0.43
QCR2_MOUSE	Cytochrome b-c1 complex subunit 2, mitochondrial	82	48205	9.26	14	5	7	2	7	0.37	0.19
ITPA_MOUSE	Inosine triphosphate pyrophosphatase	82	21883	5.6	5	2	2	1	2	0.15	0.21
TWF1_MOUSE	Twinfilin-1	82	40054	6.21	7	4	4	3	4	0.19	0.37
SEPT9_MOUSE	Septin-9	82	65534	9.01	16	5	9	3	9	0.23	0.21
PGFS_MOUSE	Prostamide/prostaglandin F synthase	81	21656	6.31	2	2	1	1	1	0.15	0.21
ECI1_MOUSE	Enoyl-CoA delta isomerase 1, mitochondrial	81	32230	9.12	3	3	2	2	2	0.13	0.29
RPE_MOUSE	Ribulose-phosphate 3-epimerase	81	24928	5.2	3	3	2	2	2	0.24	0.39
RL13_MOUSE	60S ribosomal protein L13	80	24290	11.54	18	6	7	4	6	0.32	0.97
RWDD1_MOUSE	RWD domain-containing protein 1	80	27768	4.18	4	2	2	1	2	0.23	0.16
CAN1_MOUSE	Calpain-1 catalytic subunit	80	82054	5.62	3	2	3	2	3	0.09	0.11
ECM29_MOUSE	Proteasome-associated protein ECM29 homolog	80	203573	6.68	10	4	7	4	7	0.08	0.09
ADA_MOUSE	Adenosine deaminase	80	39966	5.48	3	2	3	2	3	0.27	0.23
PSMD7_MOUSE	26S proteasome non-ATPase regulatory subunit 7	80	36517	6.29	5	3	5	3	5	0.34	0.41
RAGP1_MOUSE	Ran GTPase-activating protein 1	80	63491	4.59	8	5	5	4	5	0.15	0.3
ACON_MOUSE	Aconitate hydratase, mitochondrial	79	85410	8.08	10	4	9	4	9	0.2	0.22
NEB2_MOUSE	Neurabin-2	79	89466	4.86	5	2	4	2	4	0.13	0.1
IF2B_MOUSE	Eukaryotic translation initiation factor 2 subunit 2	79	38068	5.61	6	4	4	3	4	0.18	0.39
NXP20_MOUSE	Protein Noxp20	78	60975	4.49	6	3	4	2	4	0.11	0.15
ABCF1_MOUSE	ATP-binding cassette sub-family F member 1	78	94887	6.15	9	3	6	3	6	0.11	0.14
HEM3_MOUSE	Porphobilinogen deaminase	78	39320	6.41	4	2	3	1	3	0.17	0.11
CSN4_MOUSE	COP9 signalosome complex subunit 4	78	46256	5.57	6	4	5	3	5	0.2	0.31
C1TM_MOUSE	Monofunctional C1-tetrahydrofolate synthase, mitochondrial	78	105662	6.58	10	2	8	1	8	0.15	0.04
SPRE_MOUSE	Sepiapterin reductase	78	27865	5.58	2	2	2	2	2	0.12	0.35
PLCD1_MOUSE	1-Phosphatidylinositol 4,5-bisphosphate phosphodiesterase delta-1	77	85819	5.82	3	2	1	1	1	0.02	0.05
IF2B2_MOUSE	Insulin-like growth factor 2 mRNA-binding protein 2	77	65543	7.81	3	2	3	2	3	0.07	0.14
MCAT_MOUSE	Mitochondrial carnitine/acylcarnitine carrier protein	77	33005	9.24	6	3	4	2	4	0.31	0.29
GSH0_MOUSE	Glutamate–cysteine ligase regulatory subunit	76	30516	5.35	6	2	5	2	5	0.32	0.31
HA1B_MOUSE	H-2 class I histocompatibility antigen, K-B alpha chain	76	41276	5.96	6	3	3	3	3	0.14	0.35
AR6P1_MOUSE	ADP-ribosylation factor-like protein 6-interacting protein 1	76	23421	9.38	3	2	2	1	2	0.19	0.19
TM9S2_MOUSE	Transmembrane 9 superfamily member 2	76	75280	7.21	6	3	5	2	5	0.14	0.12
SFXN1_MOUSE	Sideroflexin-1	76	35626	9.35	4	1	4	1	4	0.26	0.12
RS21_MOUSE	40S ribosomal protein S21	76	9136	8.71	7	3	4	1	4	0.54	0.56
IL4RA_MOUSE	Interleukin-4 receptor subunit alpha	76	87571	4.97	3	2	2	1	2	0.04	0.05
EIF1_MOUSE	Eukaryotic translation initiation factor 1	75	12739	6.89	3	2	2	2	2	0.24	0.89
NUCB1_MOUSE	Nucleobindin-1	75	53376	4.99	17	4	11	2	11	0.34	0.17
MMS19_MOUSE	MMS19 nucleotide excision repair protein homolog	75	113017	5.81	4	2	3	2	3	0.07	0.08
GCP60_MOUSE	Golgi resident protein GCP60	75	60144	5.07	12	5	5	2	5	0.2	0.15
FDFT_MOUSE	Squalene synthase	75	48123	5.91	9	3	6	2	6	0.27	0.3
TPD52_MOUSE	Tumor protein D52	75	24298	4.69	4	3	3	2	3	0.25	0.4
RBM3_MOUSE	RNA-binding protein 3	75	16595	6.84	14	7	5	4	5	0.57	1.68
RBMS1_MOUSE	RNA-binding motif, single-stranded-interacting protein 1	75	43963	8.79	3	2	3	2	3	0.21	0.21
NDUAA_MOUSE	NADH dehydrogenase [ubiquinone] 1 alpha subcomplex subunit 10, mitochondrial	75	40578	7.63	5	3	5	3	5	0.23	0.36
SRC8_MOUSE	Src substrate cortactin	75	61212	5.24	10	2	8	2	8	0.15	0.15
HNRPC_MOUSE	Heterogeneous nuclear ribonucleoproteins C1/C2	74	34364	4.92	2	1	2	1	2	0.09	0.13
CP20A_MOUSE	Cytochrome P450 20A1	74	52116	6.48	5	3	4	2	4	0.19	0.17
LDLR_MOUSE	Low-density lipoprotein receptor	74	94885	4.82	5	2	5	2	5	0.13	0.09
MARC2_MOUSE	Mitochondrial amidoxime reducing component 2	73	38170	8.95	2	2	2	2	2	0.15	0.24
ERF3A_MOUSE	Eukaryotic peptide chain release factor GTP-binding subunit ERF3A	73	68582	5.12	13	6	9	4	9	0.22	0.28
ALD2_MOUSE	Aldose reductase-related protein 2	73	36098	5.97	4	2	4	2	4	0.2	0.26
ADPRH_MOUSE	[Protein ADP-ribosylarginine] hydrolase	73	40042	5.46	3	2	3	2	3	0.22	0.23
ATP5I_MOUSE	ATP synthase subunit e, mitochondrial	72	8230	9.34	9	4	4	2	4	0.62	1.66
GLU2B_MOUSE	Glucosidase 2 subunit beta	72	58756	4.41	12	5	8	4	8	0.24	0.33
ACSL4_MOUSE	Long-chain-fatty-acid–CoA ligase 4	72	79026	8.57	12	5	8	3	6	0.19	0.17
PUF60_MOUSE	Poly(U)-binding-splicing factor PUF60	72	60211	5.2	6	2	5	1	5	0.24	0.07
PTPA_MOUSE	Serine/threonine-protein phosphatase 2A activator	72	36687	5.95	5	2	4	1	4	0.29	0.12
PDXK_MOUSE	Pyridoxal kinase	72	34993	5.88	4	2	4	2	4	0.19	0.27
AP1G1_MOUSE	AP-1 complex subunit gamma-1	71	91292	6.36	6	4	5	3	5	0.16	0.15
ATG3_MOUSE	Ubiquitin-like-conjugating enzyme ATG3	71	35773	4.63	4	2	3	1	3	0.28	0.26
KAP0_MOUSE	cAMP-dependent protein kinase type I-alpha regulatory subunit	71	43158	5.27	9	2	8	2	8	0.28	0.21
IF4B_MOUSE	Eukaryotic translation initiation factor 4B	71	68799	5.47	8	6	2	2	2	0.07	0.13
NPL4_MOUSE	Nuclear protein localization protein 4 homolog	71	67974	6.01	3	1	3	1	3	0.11	0.06
CX6A1_MOUSE	Cytochrome c oxidase subunit 6A1, mitochondrial	71	12344	9.97	3	2	2	1	2	0.45	0.39
CDC37_MOUSE	Hsp90 co-chaperone Cdc37	71	44565	5.24	6	4	5	3	5	0.21	0.32
AN32A_MOUSE	Acidic leucine-rich nuclear phosphoprotein 32 family member A	71	28520	3.99	4	2	4	2	4	0.22	0.34
PA24A_MOUSE	Cytosolic phospholipase A2	71	85168	5.28	6	2	6	2	6	0.18	0.1
CO5A2_MOUSE	Collagen alpha-2(V) chain	70	144929	6.28	17	7	9	5	9	0.14	0.16
ANXA7_MOUSE	Annexin A7	70	49893	5.91	10	3	8	2	8	0.22	0.18
AIF1L_MOUSE	Allograft inflammatory factor 1-like	70	17013	6.63	3	2	2	1	2	0.16	0.27
MECR_MOUSE	Enoyl-[acyl-carrier-protein] reductase, mitochondrial	70	40317	9.17	3	3	1	1	1	0.09	0.11
MPI_MOUSE	Mannose-6-phosphate isomerase	70	46545	5.62	2	1	2	1	2	0.1	0.09
SUCB2_MOUSE	Succinate–CoA ligase [GDP-forming] subunit beta, mitochondrial	70	46811	6.58	4	1	4	1	4	0.14	0.09
MMAB_MOUSE	Cob(I)yrinic acid a,c-diamide adenosyltransferase, mitochondrial	69	26256	9.32	2	1	2	1	2	0.16	0.17
AGAL_MOUSE	Alpha-galactosidase A	69	47611	5.44	4	2	4	2	4	0.14	0.19
SP16H_MOUSE	FACT complex subunit SPT16	69	119749	5.5	4	1	4	1	4	0.07	0.04
NP1L4_MOUSE	Nucleosome assembly protein 1-like 4	69	42653	4.56	9	3	7	3	5	0.32	0.34
UBQL1_MOUSE	Ubiquilin-1	69	61937	4.86	10	4	6	3	6	0.24	0.22
RET1_MOUSE	Retinol-binding protein 1	69	15836	5.1	4	2	4	2	4	0.44	0.68
ABHEB_MOUSE	Protein ABHD14B	69	22437	5.82	2	1	2	1	2	0.1	0.2
HM13_MOUSE	Minor histocompatibility antigen H13	69	41721	5.7	4	2	2	1	2	0.1	0.1
GALM_MOUSE	Aldose 1-epimerase	68	37775	6.26	4	2	3	2	3	0.13	0.25
AP3B1_MOUSE	AP-3 complex subunit beta-1	68	122664	5.49	13	3	9	3	9	0.11	0.11
LEG9_MOUSE	Galectin-9	68	40010	9.41	9	3	6	3	6	0.24	0.37
SPART_MOUSE	Spartin	68	72610	5.64	3	2	3	2	3	0.09	0.12
ERAP1_MOUSE	Endoplasmic reticulum aminopeptidase 1	67	106531	5.82	7	3	5	3	5	0.09	0.12
PIEZ1_MOUSE	Piezo-type mechanosensitive ion channel component 1	67	291813	7.41	2	1	2	1	2	0.01	0.01
AL1L2_MOUSE	Mitochondrial 10-formyltetrahydrofolate dehydrogenase	67	101526	5.93	8	3	7	3	6	0.13	0.13
PDLI7_MOUSE	PDZ and LIM domain protein 7	67	50087	8.82	5	1	5	1	5	0.23	0.09
ADA15_MOUSE	Disintegrin and metalloproteinase domain-containing protein 15	67	92604	6.09	5	2	3	1	3	0.09	0.05
PGPI_MOUSE	Pyroglutamyl-peptidase 1	66	22919	5.21	4	3	4	3	4	0.43	0.72
ANTR1_MOUSE	Anthrax toxin receptor 1	66	62269	7.53	3	1	3	1	3	0.08	0.07
OSTF1_MOUSE	Osteoclast-stimulating factor 1	66	23768	5.46	4	2	4	2	4	0.3	0.42
IL1RA_MOUSE	Interleukin-1 receptor antagonist protein	66	20261	5.82	3	2	3	2	3	0.42	0.5
CC90B_MOUSE	Coiled-coil domain-containing protein 90B, mitochondrial	66	29578	8.54	3	1	3	1	3	0.25	0.15
PON2_MOUSE	Serum paraoxonase/arylesterase 2	66	39592	5.49	5	3	3	1	3	0.18	0.11
PGH1_MOUSE	Prostaglandin G/H synthase 1	66	68998	6.36	7	5	6	5	6	0.12	0.35
NRADD_MOUSE	Death domain-containing membrane protein NRADD	66	24711	4.89	2	2	1	1	1	0.14	0.18
PEA15_MOUSE	Astrocytic phosphoprotein PEA-15	66	15045	4.94	9	3	4	3	4	0.45	1.26
B2MG_MOUSE	Beta-2-microglobulin	66	13770	8.55	3	2	3	2	3	0.35	0.81
ACADM_MOUSE	Medium-chain specific acyl-CoA dehydrogenase, mitochondrial	66	46452	8.6	4	2	4	2	4	0.18	0.2
SCRB2_MOUSE	Lysosome membrane protein 2	65	54009	4.99	5	2	5	2	5	0.12	0.17
AATC_MOUSE	Aspartate aminotransferase, cytoplasmic	65	46219	6.68	14	3	10	3	10	0.34	0.31
ERO1A_MOUSE	ERO1-like protein alpha	65	54050	6.12	6	3	4	2	4	0.14	0.17
PUR8_MOUSE	Adenylosuccinate lyase	65	54831	6.9	6	3	5	2	5	0.19	0.16
LSG1_MOUSE	Large subunit GTPase 1 homolog	64	73111	6.08	5	2	4	1	4	0.1	0.06
NAGAB_MOUSE	Alpha-N-acetylgalactosaminidase	64	47204	6.02	4	2	3	1	3	0.14	0.09
IDHG1_MOUSE	Isocitrate dehydrogenase [NAD] subunit gamma 1, mitochondrial	64	42758	9.17	2	1	2	1	2	0.09	0.1
CTGF_MOUSE	Connective tissue growth factor	64	37798	8.22	14	6	8	3	8	0.33	0.39
EFTS_MOUSE	Elongation factor Ts, mitochondrial	64	35312	6.62	5	3	4	2	4	0.25	0.27
DBNL_MOUSE	Drebrin-like protein	64	48670	4.9	5	1	4	1	4	0.13	0.09
TENA_MOUSE	Tenascin	64	231659	4.77	11	3	8	2	8	0.09	0.04
SYTC_MOUSE	Threonine–tRNA ligase, cytoplasmic	64	83303	7.03	6	4	5	4	5	0.12	0.22
TMM59_MOUSE	Transmembrane protein 59	64	36290	4.8	2	1	2	1	2	0.12	0.12
ARK72_MOUSE	Aflatoxin B1 aldehyde reductase member 2	63	40586	8.36	2	2	1	1	1	0.1	0.11
PR2C3_MOUSE	Prolactin-2C3	63	25322	5.61	9	2	5	1	5	0.55	0.18
AL7A1_MOUSE	Alpha-aminoadipic semialdehyde dehydrogenase	62	58824	7.16	5	4	5	4	5	0.15	0.33
STAT1_MOUSE	Signal transducer and activator of transcription 1	49	87142	5.42	8	4	6	3	3	0.14	0.15
FMR1_MOUSE	Synaptic functional regulator FMR1	62	68947	7.27	7	2	6	2	5	0.17	0.13
EGFR_MOUSE	Epidermal growth factor receptor	59	134766	6.46	7	2	7	2	7	0.09	0.06
DCAKD_MOUSE	Dephospho-CoA kinase domain-containing protein	61	26459	9.61	4	2	3	1	3	0.2	0.17
TIM13_MOUSE	Mitochondrial import inner membrane translocase subunit Tim13	61	10451	8.42	6	2	2	1	2	0.26	0.47
ITAM_MOUSE	Integrin alpha-M	61	127400	6.87	4	2	3	1	3	0.04	0.03
ARP10_MOUSE	Actin-related protein 10	61	46178	7.54	2	2	2	2	2	0.05	0.2
TMED5_MOUSE	Transmembrane emp24 domain-containing protein 5	61	26155	4.81	5	2	4	2	4	0.31	0.37
TRXR1_MOUSE	Thioredoxin reductase 1, cytoplasmic	61	67042	7.42	9	6	7	5	7	0.22	0.37
NAGK_MOUSE	N-acetyl-D-glucosamine kinase	60	37245	5.43	5	3	3	2	3	0.23	0.25
GAPR1_MOUSE	Golgi-associated plant pathogenesis-related protein 1	60	17080	9.54	5	2	3	1	3	0.32	0.27
ODO2_MOUSE	Dihydrolipoyllysine-residue succinyltransferase component of 2-oxoglutarate dehydrogenase complex, mitochondrial	60	48963	9.11	8	2	7	2	7	0.2	0.19
STX7_MOUSE	Syntaxin-7	60	29802	5.6	9	4	5	2	5	0.15	0.32
GID8_MOUSE	Glucose-induced degradation protein 8 homolog	60	26762	4.92	4	2	3	1	3	0.3	0.17
PGBM_MOUSE	Basement membrane-specific heparan sulfate proteoglycan core protein	60	398039	5.88	10	4	8	3	8	0.05	0.04
MDR1A_MOUSE	Multidrug resistance protein 1A	60	140558	8.94	4	1	3	1	3	0.04	0.03
CASP1_MOUSE	Caspase-1	59	45611	5.73	4	2	3	1	3	0.15	0.1
SYCC_MOUSE	Cysteine–tRNA ligase, cytoplasmic	59	94800	6.32	13	3	8	3	8	0.15	0.14
PRRX1_MOUSE	Paired mesoderm homeobox protein 1	59	27253	9.48	2	2	1	1	1	0.12	0.16
LY6A_MOUSE	Lymphocyte antigen 6A-2/6E-1	59	14367	4.75	4	3	1	1	1	0.16	0.33
AMPD3_MOUSE	AMP deaminase 3	59	88596	6.87	4	1	4	1	4	0.08	0.05
NDRG2_MOUSE	Protein NDRG2	59	40763	5.23	2	1	2	1	2	0.12	0.11
S39AE_MOUSE	Zinc transporter ZIP14	59	53927	5.11	1	1	1	1	1	0.06	0.08
PLPHP_MOUSE	Pyridoxal phosphate homeostasis protein	59	30030	8.37	4	4	4	4	4	0.33	0.74
PSMD3_MOUSE	26S proteasome non-ATPase regulatory subunit 3	59	60680	8.48	7	2	6	2	6	0.18	0.15
TRADD_MOUSE	Tumor necrosis factor receptor type 1-associated DEATH domain protein	59	34556	5.11	5	1	3	1	3	0.12	0.13
ANM5_MOUSE	Protein arginine N-methyltransferase 5	59	72634	5.99	5	3	3	2	3	0.13	0.12
IPO9_MOUSE	Importin-9	58	115978	4.71	6	2	4	2	4	0.09	0.07
VPS52_MOUSE	Vacuolar protein sorting-associated protein 52 homolog	58	81993	5.65	7	2	3	1	3	0.07	0.05
LRRF1_MOUSE	Leucine-rich repeat flightless-interacting protein 1	58	79201	4.75	2	1	2	1	2	0.08	0.05
E41L3_MOUSE	Band 4.1-like protein 3	58	103274	5.2	5	2	4	2	4	0.05	0.08
EMIL1_MOUSE	EMILIN-1	58	107518	5.21	3	1	3	1	3	0.06	0.04
EEA1_MOUSE	Early endosome antigen 1	58	160817	5.59	6	2	5	2	5	0.06	0.05
MESD_MOUSE	LRP chaperone MESD	58	25191	6.06	2	2	1	1	1	0.06	0.18
IMPCT_MOUSE	Protein IMPACT	57	36253	4.97	3	2	3	2	3	0.15	0.26
GUAA_MOUSE	GMP synthase [glutamine-hydrolyzing]	57	76675	6.29	4	2	3	1	3	0.11	0.06
GCSH_MOUSE	Glycine cleavage system H protein, mitochondrial	57	18625	4.78	3	3	1	1	1	0.22	0.55
VA0D1_MOUSE	V-type proton ATPase subunit d 1	57	40275	4.89	9	4	8	4	8	0.34	0.51
PUR2_MOUSE	Trifunctional purine biosynthetic protein adenosine-3	57	107436	6.25	6	3	5	2	5	0.1	0.08
GLOD4_MOUSE	Glyoxalase domain-containing protein 4	57	33296	5.28	2	2	2	2	2	0.17	0.28
WDR61_MOUSE	WD repeat-containing protein 61	57	33752	5.1	3	2	3	2	3	0.18	0.28
CUL5_MOUSE	Cullin-5	57	90916	7.86	10	2	6	1	6	0.17	0.05
NICA_MOUSE	Nicastrin	57	78443	5.75	5	2	4	2	4	0.06	0.11
FKB14_MOUSE	Peptidyl-prolyl cis-trans isomerase FKBP14	57	24236	5.66	4	2	3	2	3	0.24	0.41
PLEK_MOUSE	Pleckstrin	57	39876	8.53	3	2	3	2	3	0.19	0.23
LA_MOUSE	Lupus La protein homolog	57	47727	9.77	5	2	5	2	5	0.13	0.19
TPBG_MOUSE	Trophoblast glycoprotein	56	46422	6.36	5	2	5	2	5	0.18	0.2
ETHE1_MOUSE	Persulfide dioxygenase ETHE1, mitochondrial	56	27721	6.78	2	1	2	1	2	0.23	0.16
UBR4_MOUSE	E3 ubiquitin-protein ligase UBR4	56	571927	5.72	20	2	16	2	16	0.07	0.01
ACSA_MOUSE	Acetyl-coenzyme A synthetase, cytoplasmic	56	78811	6.19	4	2	3	1	3	0.09	0.05
PSA_MOUSE	Puromycin-sensitive aminopeptidase	56	103260	5.61	7	4	6	3	6	0.14	0.13
S10A4_MOUSE	Protein S100-A4	56	11714	5.23	4	2	2	1	2	0.23	0.41
P3H3_MOUSE	Prolyl 3-hydroxylase 3	55	81650	6.21	3	1	3	1	3	0.08	0.05
LMNA_MOUSE	Prelamin-A/C	55	74193	6.54	10	1	5	1	5	0.11	0.06
VAC14_MOUSE	Protein VAC14 homolog	54	87992	5.75	5	1	4	1	4	0.09	0.05
PYR1_MOUSE	CAD protein	54	243084	6	13	5	11	4	11	0.11	0.07
ABRAL_MOUSE	Costars family protein ABRACL	54	9025	5.52	4	1	3	1	3	0.57	0.56
CTND1_MOUSE	Catenin delta-1	54	104860	6.41	8	2	8	2	8	0.12	0.08
FKB15_MOUSE	FK506-binding protein 15	54	132878	4.98	4	2	3	1	3	0.06	0.03
ECE1_MOUSE	Endothelin-converting enzyme 1	54	87017	5.64	5	5	5	5	5	0.17	0.27
AT5F1_MOUSE	ATP synthase F(0) complex subunit B1, mitochondrial	54	28930	9.11	8	2	6	2	6	0.27	0.33
LDAH_MOUSE	Lipid droplet-associated hydrolase	54	37349	8.5	3	1	2	1	2	0.18	0.12
TNIK_MOUSE	Traf2 and NCK-interacting protein kinase	54	150274	6.82	9	2	7	1	7	0.07	0.03
EI2BA_MOUSE	Translation initiation factor eIF-2B subunit alpha	54	33795	8.48	4	3	2	1	2	0.1	0.13
VAPA_MOUSE	Vesicle-associated membrane protein-associated protein A	53	27837	8.59	9	2	6	2	6	0.36	0.35
DYLT3_MOUSE	Dynein light chain Tctex-type 3	53	12949	4.98	1	1	1	1	1	0.29	0.37
ZZEF1_MOUSE	Zinc finger ZZ-type and EF-hand domain-containing protein 1	53	328102	5.74	6	2	4	1	4	0.03	0.01
NDRG1_MOUSE	Protein NDRG1	53	42981	5.69	5	2	4	2	4	0.21	0.21
PDLI5_MOUSE	PDZ and LIM domain protein 5	53	63259	8.61	15	3	7	3	7	0.27	0.22
LIMA1_MOUSE	LIM domain and actin-binding protein 1	53	84008	6.18	10	2	7	2	7	0.12	0.1
NPC2_MOUSE	Epididymal secretory protein E1	53	16432	7.59	13	3	8	2	8	0.7	0.65
AN32B_MOUSE	Acidic leucine-rich nuclear phosphoprotein 32 family member B	53	31060	3.89	2	1	2	1	2	0.11	0.14
ERP44_MOUSE	Endoplasmic reticulum resident protein 44	53	46823	5.09	6	2	6	2	6	0.24	0.19
CBR2_MOUSE	Carbonyl reductase [NADPH] 2	53	25942	9.1	2	2	2	2	2	0.23	0.38
TNR12_MOUSE	Tumor necrosis factor receptor superfamily member 12A	53	13632	8.18	2	1	2	1	2	0.43	0.35
GGT5_MOUSE	Glutathione hydrolase 5 proenzyme	53	61635	8.76	3	1	3	1	3	0.13	0.07
AL3A2_MOUSE	Fatty aldehyde dehydrogenase	52	53936	8.59	2	2	2	2	2	0.09	0.17
MYDGF_MOUSE	Myeloid-derived growth factor	52	17971	6.3	5	2	2	1	2	0.12	0.26
FKBP4_MOUSE	Peptidyl-prolyl cis-trans isomerase FKBP4	52	51540	5.54	4	2	3	1	3	0.13	0.08
AL1L1_MOUSE	Cytosolic 10-formyltetrahydrofolate dehydrogenase	52	98647	5.64	9	2	7	2	6	0.17	0.09
TCP4_MOUSE	Activated RNA polymerase II transcriptional coactivator p15	52	14418	9.6	5	2	3	1	3	0.28	0.33
GLO2_MOUSE	Hydroxyacylglutathione hydrolase, mitochondrial	51	34062	7.66	4	2	2	1	2	0.14	0.28
PARK7_MOUSE	Protein/nucleic acid deglycase DJ-1	51	20008	6.32	12	5	6	4	6	0.6	1.28
DHSO_MOUSE	Sorbitol dehydrogenase	51	38225	6.56	1	1	1	1	1	0.03	0.11
TENS3_MOUSE	Tensin-3	51	155491	6.19	6	1	4	1	4	0.07	0.03
RSU1_MOUSE	Ras suppressor protein 1	51	31531	8.86	5	1	5	1	5	0.35	0.14
PAK2_MOUSE	Serine/threonine-protein kinase PAK 2	49	57894	5.57	5	3	4	2	4	0.14	0.15
HSBP1_MOUSE	Heat shock factor-binding protein 1	51	8605	4.11	3	2	2	1	2	0.58	0.59
TMX3_MOUSE	Protein disulfide-isomerase TMX3	51	51815	5.02	4	2	4	2	4	0.17	0.17
CREG1_MOUSE	Protein CREG1	51	24436	5.96	4	3	3	3	3	0.23	0.66
MRP1_MOUSE	Multidrug resistance-associated protein 1	50	171075	7.03	2	1	2	1	2	0.04	0.02
NU4M_MOUSE	NADH-ubiquinone oxidoreductase chain 4	50	51847	9.42	4	3	3	2	3	0.11	0.17
TIMP2_MOUSE	Metalloproteinase inhibitor 2	50	24312	7.45	6	2	6	2	6	0.5	0.4
NUP93_MOUSE	Nuclear pore complex protein Nup93	50	93222	5.5	5	2	5	2	5	0.09	0.09
TGBR3_MOUSE	Transforming growth factor beta receptor type 3	50	93769	5.65	7	4	4	2	4	0.1	0.14
RM46_MOUSE	39S ribosomal protein L46, mitochondrial	50	32112	6.93	4	1	3	1	3	0.14	0.14
SRXN1_MOUSE	Sulfiredoxin-1	50	14140	7.82	4	1	2	1	2	0.26	0.34
SYQ_MOUSE	Glutamine–tRNA ligase	50	87621	6.93	9	2	6	1	6	0.16	0.05
SORCN_MOUSE	Sorcin	50	21613	5.32	10	2	4	1	4	0.26	0.21
NDUA2_MOUSE	NADH dehydrogenase [ubiquinone] 1 alpha subcomplex subunit 2	50	10909	10.02	3	2	1	1	1	0.14	0.45
NASP_MOUSE	Nuclear autoantigenic sperm protein	50	83903	4.35	4	2	4	2	4	0.1	0.1
A16A1_MOUSE	Aldehyde dehydrogenase family 16 member A1	49	84703	5.9	1	1	1	1	1	0.04	0.05
NQO1_MOUSE	NAD(P)H dehydrogenase [quinone] 1	49	30940	8.74	12	4	6	2	6	0.36	0.31
NRBP_MOUSE	Nuclear receptor-binding protein	49	59828	5.02	1	1	1	1	1	0.06	0.07
NIF3L_MOUSE	NIF3-like protein 1	49	41719	6.28	5	2	3	1	3	0.12	0.1
NDUS4_MOUSE	NADH dehydrogenase [ubiquinone] iron-sulfur protein 4, mitochondrial	49	19772	10	3	1	3	1	2	0.26	0.23
CERS2_MOUSE	Ceramide synthase 2	49	44995	8.85	1	1	1	1	1	0.05	0.1
GT251_MOUSE	Procollagen galactosyltransferase 1	49	71015	6.83	11	4	9	4	9	0.28	0.27
S23IP_MOUSE	SEC23-interacting protein	49	110711	5.63	4	1	3	1	3	0.07	0.04
DDRGK_MOUSE	DDRGK domain-containing protein 1	49	35956	5.32	3	1	2	1	2	0.13	0.12
LMAN1_MOUSE	Protein ERGIC-53	49	57753	5.92	11	5	7	4	7	0.25	0.34
AHSA1_MOUSE	Activator of 90 kDa heat shock protein ATPase homolog 1	49	38093	5.41	5	2	5	2	5	0.27	0.24
HMGCL_MOUSE	Hydroxymethylglutaryl-CoA lyase, mitochondrial	48	34217	8.7	3	2	3	2	3	0.2	0.27
PURA2_MOUSE	Adenylosuccinate synthetase isozyme 2	48	49990	5.98	6	2	5	2	5	0.16	0.18
SYK_MOUSE	Lysine–tRNA ligase	48	67796	5.65	6	1	4	1	4	0.11	0.06
CGAT1_MOUSE	Chondroitin sulfate N-acetylgalactosaminyltransferase 1	48	60878	9.03	1	1	1	1	1	0.03	0.07
NSDHL_MOUSE	Sterol-4-alpha-carboxylate 3-dehydrogenase, decarboxylating	48	40660	7.71	6	4	4	3	4	0.19	0.36
KTN1_MOUSE	Kinectin	48	152498	5.67	11	1	11	1	11	0.12	0.03
BID_MOUSE	BH3-interacting domain death agonist	48	21938	4.71	2	2	2	2	2	0.15	0.46
DEGS1_MOUSE	Sphingolipid delta(4)-desaturase DES1	45	38216	7.33	2	2	2	2	2	0.1	0.24
EP15R_MOUSE	Epidermal growth factor receptor substrate 15-like 1	47	99248	4.86	8	2	6	1	6	0.17	0.04
ARL2_MOUSE	ADP-ribosylation factor-like protein 2	47	20851	5.67	1	1	1	1	1	0.14	0.22
NPC1_MOUSE	Niemann-Pick C1 protein	47	142791	5.44	5	2	4	2	4	0.06	0.06
WASC2_MOUSE	WASH complex subunit 2	47	145224	4.63	4	2	4	2	4	0.05	0.06
NPL_MOUSE	N-Acetylneuraminate lyase	47	35108	7.74	3	2	1	1	1	0.09	0.27
CD63_MOUSE	CD63 antigen	47	25749	6.69	4	2	4	2	4	0.23	0.38
GLRX3_MOUSE	Glutaredoxin-3	47	37754	5.42	12	3	11	3	11	0.6	0.39
DNJB4_MOUSE	DnaJ homolog subfamily B member 4	47	37758	8.7	2	1	2	1	2	0.08	0.12
DC1L2_MOUSE	Cytoplasmic dynein 1 light intermediate chain 2	46	54185	5.89	7	3	7	3	6	0.25	0.26
ULA1_MOUSE	NEDD8-activating enzyme E1 regulatory subunit	46	60236	5.34	6	3	4	2	4	0.16	0.23
SETD3_MOUSE	Histone-lysine N-methyltransferase setd3	44	67134	5.47	2	2	2	2	2	0.08	0.13
LXN_MOUSE	Latexin	46	25476	5.48	2	1	2	1	2	0.14	0.18
MPEG1_MOUSE	Macrophage-expressed gene 1 protein	46	78340	5.64	4	2	3	1	3	0.1	0.05
YKT6_MOUSE	Synaptobrevin homolog YKT6	46	22300	5.97	4	1	4	1	4	0.35	0.2
GSHR_MOUSE	Glutathione reductase, mitochondrial	46	53629	8.19	8	2	6	1	6	0.19	0.08
CP062_MOUSE	UPF0505 protein C16orf62 homolog	46	109007	6.97	3	1	3	1	3	0.06	0.04
SCMC1_MOUSE	Calcium-binding mitochondrial carrier protein SCaMC-1	46	52868	7.02	7	2	6	2	6	0.23	0.17
CO4A2_MOUSE	Collagen alpha-2(IV) chain	46	167220	8.75	4	1	4	1	4	0.04	0.03
RDH13_MOUSE	Retinol dehydrogenase 13	46	36441	9.02	2	1	2	1	2	0.08	0.12
DPP2_MOUSE	Dipeptidyl peptidase 2	46	56218	5.17	1	1	1	1	1	0.03	0.08
ATX10_MOUSE	Ataxin-10	46	53673	5.12	5	2	4	2	4	0.12	0.17
APMAP_MOUSE	Adipocyte plasma membrane-associated protein	45	46405	5.97	2	2	1	1	1	0.07	0.09
EFHD2_MOUSE	EF-hand domain-containing protein D2	45	26775	5.01	8	1	3	1	3	0.12	0.17
PEDF_MOUSE	Pigment epithelium-derived factor	45	46205	6.48	3	1	2	1	2	0.1	0.09
SODC_MOUSE	Superoxide dismutase [Cu-Zn]	45	15933	6.02	9	3	5	2	5	0.39	0.67
S38A2_MOUSE	Sodium-coupled neutral amino acid transporter 2	45	55467	8.05	4	2	4	2	4	0.16	0.16
NUP50_MOUSE	Nuclear pore complex protein Nup50	45	49455	5.94	3	1	3	1	3	0.16	0.09
CD44_MOUSE	CD44 antigen	45	85565	4.82	6	1	5	1	5	0.11	0.05
NDUS8_MOUSE	NADH dehydrogenase [ubiquinone] iron-sulfur protein 8, mitochondrial	45	24023	5.89	1	1	1	1	1	0.04	0.19
STX2_MOUSE	Syntaxin-2	45	33157	5.98	2	1	1	1	1	0.11	0.13
TMED3_MOUSE	Transmembrane emp24 domain-containing protein 3	45	25449	5.62	3	2	2	2	2	0.16	0.38
VMP1_MOUSE	Vacuole membrane protein 1	45	45931	6.47	1	1	1	1	1	0.07	0.09
PRAF1_MOUSE	Prenylated Rab acceptor protein 1	45	20606	7.74	1	1	1	1	1	0.08	0.22
ARMC6_MOUSE	Armadillo repeat-containing protein 6	44	50651	5.65	2	1	2	1	2	0.12	0.09
GRHPR_MOUSE	Glyoxylate reductase/hydroxypyruvate reductase	44	35306	7.57	3	3	2	2	2	0.16	0.27
CRTAP_MOUSE	Cartilage-associated protein	44	46140	5.46	3	3	3	3	3	0.14	0.31
TM14C_MOUSE	Transmembrane protein 14C	44	11634	9.7	2	2	2	2	2	0.51	1.01
COX5B_MOUSE	Cytochrome c oxidase subunit 5B, mitochondrial	44	13804	8.69	10	4	3	2	3	0.34	0.81
NNRE_MOUSE	NAD(P)H-hydrate epimerase	44	30953	7.59	2	2	2	2	2	0.1	0.31
TPR_MOUSE	Nucleoprotein TPR	44	273824	4.98	10	1	10	1	10	0.06	0.02
SYFA_MOUSE	Phenylalanine–tRNA ligase alpha subunit	44	57563	8.27	3	1	2	1	2	0.05	0.08
E41L2_MOUSE	Band 4.1-like protein 2	43	109873	5.31	7	1	7	1	7	0.09	0.04
CBR3_MOUSE	Carbonyl reductase [NADPH] 3	43	30934	6.15	4	2	3	2	3	0.19	0.31
NDUB7_MOUSE	NADH dehydrogenase [ubiquinone] 1 beta subcomplex subunit 7	43	16320	8.35	5	2	2	2	2	0.26	0.65
LRN4L_MOUSE	LRRN4 C-terminal-like protein	43	25914	5.15	2	1	2	1	2	0.11	0.17
CSTFT_MOUSE	Cleavage stimulation factor subunit 2 tau variant	43	65820	6.78	4	1	3	1	3	0.13	0.07
RFA2_MOUSE	Replication protein A 32 kDa subunit	43	29700	5.76	1	1	1	1	1	0.09	0.15
NPS3B_MOUSE	Protein NipSnap homolog 3B	43	28290	9.51	2	2	1	1	1	0.1	0.16
HDAC7_MOUSE	Histone deacetylase 7	43	101224	7.11	2	1	2	1	2	0.07	0.04
PEX19_MOUSE	Peroxisomal biogenesis factor 19	43	32713	4.26	4	1	4	1	4	0.34	0.14
GALK1_MOUSE	Galactokinase	43	42268	5.17	7	2	5	2	5	0.21	0.22
DCTN1_MOUSE	Dynactin subunit 1	43	141588	5.66	7	2	6	2	6	0.08	0.06
OAS1A_MOUSE	2′-5′-Oligoadenylate synthase 1A	43	42402	8.3	2	1	2	1	2	0.08	0.1
FKBP3_MOUSE	Peptidyl-prolyl cis-trans isomerase FKBP3	43	25132	9.29	3	2	3	2	3	0.21	0.39
GIPC1_MOUSE	PDZ domain-containing protein GIPC1	43	36107	5.65	3	2	3	2	3	0.17	0.26
EMC3_MOUSE	ER membrane protein complex subunit 3	43	29960	6.33	3	1	3	1	3	0.17	0.15
MMSA_MOUSE	Methylmalonate-semialdehyde dehydrogenase [acylating], mitochondrial	43	57878	8.29	2	1	2	1	2	0.06	0.07
PLXB2_MOUSE	Plexin-B2	43	206099	5.58	12	1	9	1	9	0.09	0.02
FADS3_MOUSE	Fatty acid desaturase 3	42	51436	7	1	1	1	1	1	0.07	0.08
FUND2_MOUSE	FUN14 domain-containing protein 2	42	16554	9.71	1	1	1	1	1	0.07	0.28
GPX7_MOUSE	Glutathione peroxidase 7	42	21048	8.42	3	1	3	1	3	0.28	0.22
MTX2_MOUSE	Metaxin-2	42	29739	5.44	2	1	2	1	2	0.16	0.15
HTR5A_MOUSE	HEAT repeat-containing protein 5A	42	219754	6.31	4	1	4	1	4	0.05	0.02
ALG11_MOUSE	GDP-Man:Man(3)GlcNAc(2)-PP-Dol alpha-1,2-mannosyltransferase	42	55234	8.58	3	1	2	1	2	0.09	0.08
DHSD_MOUSE	Succinate dehydrogenase [ubiquinone] cytochrome b small subunit, mitochondrial	42	17003	9.3	4	2	2	1	2	0.23	0.27
UBP19_MOUSE	Ubiquitin carboxyl-terminal hydrolase 19	42	150454	5.99	3	1	3	1	3	0.04	0.03
OSMR_MOUSE	Oncostatin-M-specific receptor subunit beta	42	110160	6.16	3	1	1	1	1	0.04	0.04
PGP_MOUSE	Glycerol-3-phosphate phosphatase	42	34519	5.21	3	1	2	1	2	0.2	0.13
DDR2_MOUSE	Discoidin domain-containing receptor 2	38	96420	5.31	3	2	2	2	2	0.05	0.09
F162A_MOUSE	Protein FAM162A	42	17713	9.9	3	1	3	1	3	0.25	0.26
ACOT9_MOUSE	Acyl-coenzyme A thioesterase 9, mitochondrial	41	50528	8.74	8	3	5	2	5	0.16	0.18
UCHL1_MOUSE	Ubiquitin carboxyl-terminal hydrolase isozyme L1	41	24822	5.14	4	2	3	1	3	0.22	0.18
TPP2_MOUSE	Tripeptidyl-peptidase 2	41	139790	6.13	9	3	7	3	7	0.11	0.09
HEXB_MOUSE	Beta-hexosaminidase subunit beta	41	61077	8.29	5	1	3	1	3	0.14	0.07
CDIPT_MOUSE	CDP-diacylglycerol–inositol 3-phosphatidyltransferase	41	23583	8.56	2	1	2	1	2	0.21	0.19
GAS6_MOUSE	Growth arrest-specific protein 6	41	74561	5.34	5	2	4	1	4	0.09	0.06
PIN1_MOUSE	Peptidyl-prolyl cis-trans isomerase NIMA-interacting 1	41	18359	8.93	2	1	2	1	2	0.19	0.25
APOE_MOUSE	Apolipoprotein E	41	35844	5.56	1	1	1	1	1	0.06	0.12
SGMR1_MOUSE	Sigma nonopioid intracellular receptor 1	41	25234	5.56	1	1	1	1	1	0.15	0.18
RTRAF_MOUSE	RNA transcription, translation and transport factor protein	41	28135	6.4	4	1	3	1	3	0.21	0.16
TF_MOUSE	Tissue factor	41	32914	9.4	5	2	5	2	5	0.26	0.29
ERGI3_MOUSE	Endoplasmic reticulum-Golgi intermediate compartment protein 3	40	43181	6.02	3	2	2	1	2	0.17	0.21
LONM_MOUSE	Lon protease homolog, mitochondrial	40	105776	6.15	6	1	5	1	5	0.14	0.04
TXD17_MOUSE	Thioredoxin domain-containing protein 17	40	14006	4.72	3	1	2	1	2	0.19	0.34
DJC10_MOUSE	DnaJ homolog subfamily C member 10	40	90525	6.53	7	2	4	2	4	0.09	0.1
PML_MOUSE	Protein PML	40	98180	5.42	4	1	4	1	4	0.09	0.04
NAGA_MOUSE	N-Acetylglucosamine-6-phosphate deacetylase	40	43473	5.78	1	1	1	1	1	0.07	0.1
HGH1_MOUSE	Protein HGH1 homolog	40	42889	4.66	1	1	1	1	1	0.06	0.1
GLRX5_MOUSE	Glutaredoxin-related protein 5, mitochondrial	40	16282	6.1	2	1	2	1	2	0.14	0.29
IPO11_MOUSE	Importin-11	40	112344	5.14	3	1	3	1	3	0.07	0.04
T176A_MOUSE	Transmembrane protein 176A	40	26579	7.62	3	1	2	1	2	0.16	0.17
S10AD_MOUSE	Protein S100-A13	40	11151	5.89	2	1	2	1	2	0.23	0.44
PEX14_MOUSE	Peroxisomal membrane protein PEX14	40	41183	5.03	2	1	2	1	2	0.15	0.11
DHRS1_MOUSE	Dehydrogenase/reductase SDR family member 1	40	33983	8.66	6	2	5	2	5	0.28	0.28
MVD1_MOUSE	Diphosphomevalonate decarboxylase	40	44044	5.89	3	2	3	2	3	0.19	0.21
VCAM1_MOUSE	Vascular cell adhesion protein 1	40	81265	5.21	5	2	3	1	3	0.09	0.05
PRDX3_MOUSE	Thioredoxin-dependent peroxide reductase, mitochondrial	40	28109	7.15	7	2	6	2	6	0.39	0.34
PP14B_MOUSE	Protein phosphatase 1 regulatory subunit 14B	40	15947	4.74	2	1	2	1	2	0.33	0.29
BPNT1_MOUSE	3′(2′),5′-Bisphosphate nucleotidase 1	39	33175	5.54	3	1	3	1	3	0.15	0.13
FAF2_MOUSE	FAS-associated factor 2	39	52438	5.35	6	1	4	1	4	0.23	0.08
TI8AB_MOUSE	Putative mitochondrial import inner membrane translocase subunit Tim8 A-B	39	11276	6.11	4	1	3	1	3	0.39	0.43
GBP2_MOUSE	Guanylate-binding protein 2	39	66697	5.56	3	1	3	1	3	0.14	0.06
ABCD3_MOUSE	ATP-binding cassette sub-family D member 3	39	75426	9.32	7	2	7	2	7	0.18	0.12
DPP9_MOUSE	Dipeptidyl peptidase 9	39	97939	6.18	3	2	2	1	2	0.05	0.04
MSMO1_MOUSE	Methylsterol monooxygenase 1	39	34750	7.29	2	1	1	1	1	0.1	0.13
TRNT1_MOUSE	CCA tRNA nucleotidyltransferase 1, mitochondrial	39	49864	8.65	3	2	2	1	2	0.07	0.09
RIPK3_MOUSE	Receptor-interacting serine/threonine-protein kinase 3	39	53289	7.22	8	3	6	3	6	0.17	0.26
ERGI1_MOUSE	Endoplasmic reticulum-Golgi intermediate compartment protein 1	39	32541	6.59	4	2	3	1	3	0.14	0.14
P2RX4_MOUSE	P2X purinoceptor 4	38	43410	8.29	2	2	1	1	1	0.07	0.1
FCGRN_MOUSE	IgG receptor FcRn large subunit p51	38	40067	5.2	2	2	2	2	2	0.1	0.23
NCF2_MOUSE	Neutrophil cytosol factor 2	38	59448	6.18	2	1	2	1	2	0.09	0.07
TOIP2_MOUSE	Torsin-1A-interacting protein 2	38	54463	4.75	2	1	2	1	2	0.12	0.08
DHB11_MOUSE	Estradiol 17-beta-dehydrogenase 11	38	32860	8.85	2	2	1	1	1	0.06	0.13
SGTA_MOUSE	Small glutamine-rich tetratricopeptide repeat-containing protein alpha	38	34301	4.99	4	2	3	2	3	0.12	0.27
HEM2_MOUSE	Delta-aminolevulinic acid dehydratase	38	36000	6.32	1	1	1	1	1	0.06	0.12
CX7A2_MOUSE	Cytochrome c oxidase subunit 7A2, mitochondrial	38	9285	10.28	3	1	3	1	3	0.53	0.54
PSB8_MOUSE	Proteasome subunit beta type-8	38	30241	6.22	4	1	4	1	4	0.19	0.15
DECR_MOUSE	2,4-Dienoyl-CoA reductase, mitochondrial	36	36191	9.1	4	2	4	2	4	0.2	0.26
XPP1_MOUSE	Xaa-Pro aminopeptidase 1	37	69547	5.33	3	2	3	2	3	0.09	0.13
SYYC_MOUSE	Tyrosine–tRNA ligase, cytoplasmic	37	59068	6.57	6	1	6	1	6	0.16	0.07
FLOT1_MOUSE	Flotillin-1	37	47484	6.71	12	2	4	1	4	0.13	0.09
BAP31_MOUSE	B cell receptor-associated protein 31	37	27939	8.73	5	1	3	1	3	0.16	0.16
NECP2_MOUSE	Adaptin ear-binding coat-associated protein 2	37	28580	7.71	9	4	4	2	4	0.28	0.34
GINM1_MOUSE	Glycoprotein integral membrane protein 1	37	36056	5.45	1	1	1	1	1	0.05	0.12
ATG7_MOUSE	Ubiquitin-like modifier-activating enzyme ATG7	37	77470	5.97	5	1	5	1	5	0.12	0.06
MA2B2_MOUSE	Epididymis-specific alpha-mannosidase	37	115537	6.9	1	1	1	1	1	0.02	0.04
TB182_MOUSE	182 kDa tankyrase-1-binding protein	37	181714	4.8	5	1	4	1	4	0.04	0.02
PICAL_MOUSE	Phosphatidylinositol-binding clathrin assembly protein	37	71498	7.71	4	1	4	1	4	0.1	0.06
PELP1_MOUSE	Proline-, glutamic acid-, and leucine-rich protein 1	37	117995	4.31	2	1	2	1	2	0.05	0.04
ARHG7_MOUSE	Rho guanine nucleotide exchange factor 7	37	96995	6.35	5	1	3	1	3	0.09	0.04
RT11_MOUSE	28S ribosomal protein S11, mitochondrial	37	20196	10.77	2	1	2	1	2	0.18	0.23
ODP2_MOUSE	Dihydrolipoyllysine-residue acetyltransferase component of pyruvate dehydrogenase complex, mitochondrial	37	67899	8.81	7	1	5	1	5	0.13	0.06
NT5C_MOUSE	5′(3′)-Deoxyribonucleotidase, cytosolic type	37	23062	5.31	2	1	2	1	2	0.22	0.2
INF2_MOUSE	Inverted formin-2	37	138474	5.09	5	1	4	1	4	0.07	0.03
WIPI1_MOUSE	WD repeat domain phosphoinositide-interacting protein 1	37	48727	6.03	2	1	2	1	2	0.09	0.09
GPDM_MOUSE	Glycerol-3-phosphate dehydrogenase, mitochondrial	37	80902	6.17	7	2	5	2	5	0.11	0.11
ATP8_MOUSE	ATP synthase protein 8	36	7761	9.88	2	1	1	1	1	0.15	0.67
BST2_MOUSE	Bone marrow stromal antigen 2	36	19140	6.82	2	2	1	1	1	0.05	0.24
DERL2_MOUSE	Derlin-2	36	27621	6.73	1	1	1	1	1	0.13	0.16
IF4G2_MOUSE	Eukaryotic translation initiation factor 4 gamma 2	36	102041	6.7	6	1	6	1	6	0.1	0.04
FKBP7_MOUSE	Peptidyl-prolyl cis-trans isomerase FKBP7	36	24897	5.69	3	2	3	2	3	0.17	0.39
PLP2_MOUSE	Proteolipid protein 2	36	16597	6.69	4	3	1	1	1	0.08	0.28
RL29_MOUSE	60S ribosomal protein L29	36	17576	11.84	5	1	5	1	5	0.34	0.26
WASH1_MOUSE	WASH complex subunit 1	36	51627	5.33	2	1	2	1	2	0.12	0.08
SIAS_MOUSE	Sialic acid synthase	36	39998	6.61	6	1	5	1	5	0.23	0.11
RT28_MOUSE	28S ribosomal protein S28, mitochondrial	36	20508	9.1	1	1	1	1	1	0.13	0.22
FACE1_MOUSE	CAAX prenyl protease 1 homolog	36	54699	6.49	2	1	2	1	2	0.09	0.08
MEP50_MOUSE	Methylosome protein 50	36	36919	5.09	1	1	1	1	1	0.09	0.12
SCFD2_MOUSE	Sec1 family domain-containing protein 2; OS = Mus musculus, GN = Scfd2, PE = 1, SV = 1	36	74703	6.35	4	1	4	1	4	0.11	0.06
PTN9_MOUSE	Tyrosine-protein phosphatase nonreceptor type 9; OS = Mus musculus, GN = Ptpn9, PE = 1, SV = 2	36	67927	8.34	3	1	2	1	2	0.07	0.06
MXRA8_MOUSE	Matrix remodeling-associated protein 8; OS = Mus musculus, GN = Mxra8, PE = 1, SV = 1	36	49719	6.66	2	1	2	1	2	0.05	0.09
COASY_MOUSE	Bifunctional coenzyme A synthase; OS = Mus musculus, GN = Coasy, PE = 1, SV = 2	36	61985	6.61	4	2	3	2	3	0.13	0.14
PFD1_MOUSE	Prefoldin subunit 1; OS = Mus musculus, GN = Pfdn1, PE = 1, SV = 1	35	14246	7.93	5	2	2	1	2	0.17	0.33
COX7C_MOUSE	Cytochrome c oxidase subunit 7C, mitochondrial; OS = Mus musculus, GN = Cox7c, PE = 1, SV = 1	35	7328	11	2	1	1	1	1	0.33	0.72
ZCCHV_MOUSE	Zinc finger CCCH-type antiviral protein 1; OS = Mus musculus, GN = Zc3hav1, PE = 1, SV = 1	35	106619	8.59	4	1	4	1	4	0.11	0.04
ASC_MOUSE	Apoptosis-associated speck-like protein containing a CARD; OS = Mus musculus, GN = Pycard, PE = 1, SV = 1	35	21445	5.26	2	1	2	1	2	0.2	0.21
KCY_MOUSE	UMP-CMP kinase; OS = Mus musculus, GN = Cmpk1, PE = 1, SV = 1	35	22151	5.68	4	2	4	2	4	0.3	0.45
HINT1_MOUSE	Histidine triad nucleotide-binding protein 1; OS = Mus musculus, GN = Hint1, PE = 1, SV = 3	35	13768	6.36	6	1	1	1	1	0.11	0.35
ARSA_MOUSE	Arylsulfatase A; OS = Mus musculus, GN = Arsa, PE = 1, SV = 2	35	53714	5.5	2	1	2	1	2	0.15	0.08
MIC13_MOUSE	MICOS complex subunit MIC13; OS = Mus musculus, GN = Mic13, PE = 1, SV = 1	35	13365	8.68	2	1	2	1	2	0.27	0.36
NUCB2_MOUSE	Nucleobindin-2 OS = Mus musculus, GN = Nucb2, PE = 1, SV = 2	35	50273	5.05	8	2	6	1	6	0.2	0.09
CHMP5_MOUSE	Charged multivesicular body protein 5; OS = Mus musculus, GN = Chmp5, PE = 1, SV = 1	35	24560	4.65	5	1	2	1	2	0.2	0.18
HOME3_MOUSE	Homer protein homolog 3 OS = Mus musculus GN = Homer3 PE = 1 SV = 2	35	39670	5.33	1	1	1	1	1	0.04	0.11
NHLC3_MOUSE	NHL repeat-containing protein 3; OS = Mus musculus, GN = Nhlrc3, PE = 1, SV = 1	34	38171	5.81	1	1	1	1	1	0.03	0.11
CTBP2_MOUSE	C-terminal-binding protein 2; OS = Mus musculus, GN = Ctbp2, PE = 1, SV = 2	34	48926	6.47	1	1	1	1	1	0.07	0.09
NU160_MOUSE	Nuclear pore complex protein Nup160; OS = Mus musculus, GN = Nup160, PE = 1, SV = 2	34	158130	5.32	5	1	4	1	4	0.04	0.03
RAB34_MOUSE	Ras-related protein Rab-34; OS = Mus musculus, GN = Rab34, PE = 1, SV = 2	34	29082	8.55	6	2	4	2	4	0.22	0.33
COMD8_MOUSE	COMM domain-containing protein 8; OS = Mus musculus, GN = Commd8, PE = 1, SV = 1	34	20839	5.38	2	1	2	1	2	0.3	0.22
HECD1_MOUSE	E3 ubiquitin-protein ligase HECTD1; OS = Mus musculus, GN = Hectd1, PE = 1, SV = 2	34	289905	5.26	12	1	11	1	11	0.08	0.01
LYRIC_MOUSE	Protein LYRIC; OS = Mus musculus, GN = Mtdh, PE = 1, SV = 1	34	63808	9.34	5	3	4	3	4	0.2	0.22
S35F6_MOUSE	Solute carrier family 35 member F6; OS = Mus musculus, GN = Slc35f6, PE = 1, SV = 1	34	40952	6.65	2	1	2	1	2	0.17	0.11
SBDS_MOUSE	Ribosome maturation protein SBDS; OS = Mus musculus, GN = Sbds, PE = 1, SV = 4	34	28762	8.92	2	1	1	1	1	0.09	0.15
RHG01_MOUSE	Rho GTPase-activating protein 1; OS = Mus musculus, GN = Arhgap1, PE = 1, SV = 1	34	50379	5.97	5	3	3	3	3	0.12	0.28
FACR1_MOUSE	Fatty acyl-CoA reductase 1; OS = Mus musculus, GN = Far1, PE = 1, SV = 1	34	59397	9.26	4	1	2	1	2	0.09	0.07
MPRD_MOUSE	Cation-dependent mannose-6-phosphate receptor; OS = Mus musculus, GN = M6pr, PE = 1, SV = 1	33	31152	5.24	3	2	2	1	2	0.13	0.14
PNKP_MOUSE	Bifunctional polynucleotide phosphatase/kinase; OS = Mus musculus, GN = Pnkp, PE = 1, SV = 2	33	57188	8.03	9	1	5	1	5	0.15	0.08
DCPS_MOUSE	m7GpppX diphosphatase; OS = Mus musculus, GN = Dcps, PE = 1, SV = 1	33	38964	6.02	2	1	2	1	2	0.1	0.11
DHX29_MOUSE	ATP-dependent RNA helicase DHX29; OS = Mus musculus, GN = Dhx29, PE = 1, SV = 1	33	153879	8.13	11	2	9	1	9	0.1	0.03
CN37_MOUSE	2′,3′-Cyclic-nucleotide 3′-phosphodiesterase; OS = Mus musculus, GN = Cnp, PE = 1, SV = 3	33	47094	9.08	1	1	1	1	1	0.07	0.09
TBCB_MOUSE	Tubulin-folding cofactor B; OS = Mus musculus, GN = Tbcb, PE = 1, SV = 2	33	27368	5.14	3	1	3	1	3	0.23	0.16
SFPQ_MOUSE	Splicing factor, proline- and glutamine-rich; OS = Mus musculus, GN = Sfpq, PE = 1, SV = 1	33	75394	9.45	3	1	2	1	2	0.07	0.06
GLYG_MOUSE	Glycogenin-1; OS = Mus musculus, GN = Gyg1, PE = 1, SV = 3	33	37378	5.06	2	1	2	1	2	0.12	0.12
TSPO_MOUSE	Translocator protein; OS = Mus musculus, GN = Tspo, PE = 1, SV = 1	33	18829	9.52	2	1	2	1	2	0.3	0.24
CND1_MOUSE	Condensin complex subunit 1; OS = Mus musculus, GN = Ncapd2, PE = 1, SV = 2	33	155567	5.98	4	1	3	1	3	0.04	0.03
JIP4_MOUSE	C-Jun-amino-terminal kinase-interacting protein 4; OS = Mus musculus, GN = Spag9, PE = 1, SV = 2	33	146129	5.05	6	1	6	1	6	0.08	0.03
NDUB9_MOUSE	NADH dehydrogenase [ubiquinone] 1 beta subcomplex subunit 9; OS = Mus musculus, GN = Ndufb9, PE = 1, SV = 3	33	21970	7.67	2	2	1	1	1	0.16	0.21
RBGP1_MOUSE	Rab GTPase-activating protein 1; OS = Mus musculus, GN = Rabgap1, PE = 1, SV = 1	32	120722	5.14	5	1	3	1	3	0.03	0.04
F13A_MOUSE	Coagulation factor XIII A chain; OS = Mus musculus, GN = F13a1, PE = 1, SV = 3	32	83155	5.63	4	3	2	2	2	0.06	0.11
DOCK7_MOUSE	Dedicator of cytokinesis protein 7; OS = Mus musculus, GN = Dock7, PE = 1, SV = 3	32	241286	6.25	6	1	6	1	6	0.04	0.02
IC1_MOUSE	Plasma protease C1 inhibitor; OS = Mus musculus, GN = Serping1, PE = 1, SV = 3	32	55549	5.87	2	1	2	1	2	0.13	0.08
SRP54_MOUSE	Signal recognition particle 54 kDa protein; OS = Mus musculus, GN = Srp54, PE = 1, SV = 2	32	55684	8.87	15	3	10	3	10	0.38	0.25
YIF1B_MOUSE	Protein YIF1B; OS = Mus musculus, GN = Yif1b, PE = 1, SV = 2	32	33961	9.19	5	1	2	1	2	0.14	0.13
TPC13_MOUSE	Trafficking protein particle complex subunit 13; OS = Mus musculus, GN = Trappc13, PE = 2, SV = 1	32	46446	5.33	3	1	2	1	2	0.12	0.09
MCL1_MOUSE	Induced myeloid leukemia cell differentiation protein Mcl-1 homolog; OS = Mus musculus, GN = Mcl1, PE = 1, SV = 3	32	35195	5.88	1	1	1	1	1	0.06	0.13
FPRP_MOUSE	Prostaglandin F2 receptor negative regulator; OS = Mus musculus, GN = Ptgfrn, PE = 1, SV = 2	32	98660	6.16	4	1	2	1	2	0.04	0.04
SPT5H_MOUSE	Transcription elongation factor SPT5; OS = Mus musculus, GN = Supt5h, PE = 1, SV = 1	32	120589	4.93	4	1	4	1	4	0.06	0.04
GPX8_MOUSE	Probable glutathione peroxidase 8; OS = Mus musculus, GN = Gpx8, PE = 1, SV = 1	32	24133	9.42	2	1	2	1	2	0.11	0.19
COR1C_MOUSE	Coronin-1C; OS = Mus musculus, GN = Coro1c, PE = 1, SV = 2	32	53087	6.65	6	1	4	1	4	0.18	0.08
T126A_MOUSE	Transmembrane protein 126A; OS = Mus musculus, GN = Tmem126a, PE = 1, SV = 1	32	21526	9.45	1	1	1	1	1	0.09	0.21
TBD2B_MOUSE	TBC1 domain family member 2B; OS = Mus musculus, GN = Tbc1d2b, PE = 1, SV = 2	32	109881	5.74	3	1	3	1	3	0.05	0.04
NDUA9_MOUSE	NADH dehydrogenase [ubiquinone] 1 alpha subcomplex subunit 9, mitochondrial; OS = Mus musculus, GN = Ndufa9, PE = 1, SV = 2	31	42498	9.75	2	1	2	1	2	0.07	0.1
GLMP_MOUSE	Glycosylated lysosomal membrane protein; OS = Mus musculus, GN = Glmp, PE = 1, SV = 1	31	43776	5.73	2	1	2	1	2	0.1	0.1
PEF1_MOUSE	Peflin; OS = Mus musculus, GN = Pef1, PE = 1, SV = 1	31	29209	5.89	3	1	2	1	2	0.15	0.15
TGM2_MOUSE	Protein-glutamine gamma-glutamyltransferase 2; OS = Mus musculus, GN = Tgm2, PE = 1, SV = 4	31	77012	4.98	3	1	3	1	3	0.05	0.06
STXB3_MOUSE	Syntaxin-binding protein 3; OS = Mus musculus, GN = Stxbp3, PE = 1, SV = 1	31	67899	8.28	1	1	1	1	1	0.03	0.06
APEH_MOUSE	Acylamino-acid-releasing enzyme; OS = Mus musculus, GN = Apeh, PE = 1, SV = 3	31	81529	5.36	5	2	5	2	5	0.14	0.11
UFSP2_MOUSE	Ufm1-specific protease 2; OS = Mus musculus, GN = Ufsp2, PE = 1, SV = 1	31	52482	6.28	1	1	1	1	1	0.05	0.08
CD166_MOUSE	CD166 antigen; OS = Mus musculus, GN = Alcam, PE = 1, SV = 3	31	65051	5.85	4	1	3	1	3	0.1	0.07
HIG1A_MOUSE	HIG1 domain family member 1A, mitochondrial; OS = Mus musculus, GN = Higd1a, PE = 1, SV = 1	31	10418	9.79	1	1	1	1	1	0.23	0.47
CYTB_MOUSE	Cystatin-B; OS = Mus musculus, GN = Cstb, PE = 1, SV = 1	31	11039	6.82	7	2	3	2	3	0.21	1.09
TIMP1_MOUSE	Metalloproteinase inhibitor 1; OS = Mus musculus, GN = Timp1, PE = 1, SV = 2	31	22613	9.14	3	1	3	1	3	0.27	0.2
SNP29_MOUSE	Synaptosomal-associated protein 29; OS = Mus musculus, GN = Snap29, PE = 1, SV = 1	31	29554	5.23	1	1	1	1	1	0.04	0.15
CDV3_MOUSE	Protein CDV3; OS = Mus musculus, GN = Cdv3, PE = 1, SV = 2	30	29711	5.84	2	1	2	1	2	0.22	0.15
FUBP1_MOUSE	Far upstream element-binding protein 1; OS = Mus musculus, GN = Fubp1, PE = 1, SV = 1	30	68497	7.74	5	1	4	1	4	0.1	0.06
FEN1_MOUSE	Flap endonuclease 1; OS = Mus musculus, GN = Fen1, PE = 1, SV = 1	30	42288	8.54	1	1	1	1	1	0.07	0.1
AAAT_MOUSE	Neutral amino acid transporter B(0); OS = Mus musculus, GN = Slc1a5, PE = 1, SV = 2	30	58445	8.14	5	1	4	1	4	0.08	0.07
EMB_MOUSE	Embigin; OS = Mus musculus, GN = Emb, PE = 1, SV = 2	30	37041	5.7	1	1	1	1	1	0.03	0.12
S2611_MOUSE	Sodium-independent sulfate anion transporter; OS = Mus musculus, GN = Slc26a11, PE = 2, SV = 2	30	64068	7.56	1	1	1	1	1	0.05	0.07
BIEA_MOUSE	Biliverdin reductase A; OS = Mus musculus, GN = Blvra, PE = 1, SV = 1	30	33504	6.53	4	1	3	1	3	0.19	0.13
TPD54_MOUSE	Tumor protein D54 OS = Mus musculus GN = Tpd52l2 PE = 1 SV = 1	30	24028	5.8	2	1	2	1	2	0.18	0.19
SHIP2_MOUSE	Phosphatidylinositol 3,4,5-trisphosphate 5-phosphatase 2; OS = Mus musculus, GN = Inppl1, PE = 1, SV = 1	30	138887	6.11	4	1	4	1	3	0.05	0.03
LTOR5_MOUSE	Ragulator complex protein LAMTOR5; OS = Mus musculus, GN = Lamtor5, PE = 1, SV = 1	30	9636	4.69	2	1	2	1	2	0.44	0.52
ZNT7_MOUSE	Zinc transporter 7; OS = Mus musculus, GN = Slc30a7, PE = 1, SV = 1	28	41763	6.33	5	2	4	2	4	0.19	0.22
COX6C_MOUSE	Cytochrome c oxidase subunit 6C; OS = Mus musculus, GN = Cox6c, PE = 1, SV = 3	30	8464	10.13	2	1	2	1	2	0.33	0.61
DRG2_MOUSE	Developmentally-regulated GTP-binding protein 2; OS = Mus musculus, GN = Drg2, PE = 1, SV = 1	29	40692	9.03	2	2	2	2	2	0.16	0.23
DYST_MOUSE	Dystonin; OS = Mus musculus, GN = Dst, PE = 1, SV = 2	29	833701	5.2	22	2	22	2	22	0.05	0.01
PSMG1_MOUSE	Proteasome assembly chaperone 1; OS = Mus musculus, GN = Psmg1, PE = 1, SV = 1	29	33083	6.05	4	2	2	2	2	0.15	0.29
FBN1_MOUSE	Fibrillin-1; OS = Mus musculus, GN = Fbn1, PE = 1, SV = 2	29	312083	4.8	27	1	24	1	24	0.19	0.01
DAG1_MOUSE	Dystroglycan; OS = Mus musculus, GN = Dag1, PE = 1, SV = 4	29	96844	8.59	4	1	3	1	3	0.04	0.04
SNX5_MOUSE	Sorting nexin-5; OS = Mus musculus, GN = Snx5, PE = 1, SV = 1	29	46768	6.19	5	1	5	1	5	0.17	0.09
TIM8B_MOUSE	Mitochondrial import inner membrane translocase subunit Tim8 B; OS = Mus musculus, GN = Timm8b, PE = 1, SV = 1	29	9281	5.02	1	1	1	1	1	0.13	0.54
PLCB3_MOUSE	1-Phosphatidylinositol 4,5-bisphosphate phosphodiesterase beta-3; OS = Mus musculus, GN = Plcb3, PE = 1, SV = 2	29	139400	5.7	6	1	6	1	6	0.09	0.03
RTCB_MOUSE	tRNA-splicing ligase RtcB homolog; OS = Mus musculus, GN = Rtcb, PE = 1, SV = 1	28	55214	6.77	5	2	4	2	4	0.19	0.16
TM9S4_MOUSE	Transmembrane 9 superfamily member 4; OS = Mus musculus, GN = Tm9sf4, PE = 1, SV = 1	28	74644	6.86	1	1	1	1	1	0.03	0.06
MBOA7_MOUSE	Lysophospholipid acyltransferase 7; OS = Mus musculus, GN = Mboat7, PE = 1, SV = 1	28	53400	8.96	2	1	1	1	1	0.05	0.08
ARPC3_MOUSE	Actin-related protein 2/3 complex subunit 3; OS = Mus musculus, GN = Arpc3, PE = 1, SV = 3	28	20511	8.78	3	1	3	1	3	0.24	0.22
RL27A_MOUSE	60S ribosomal protein L27a; OS = Mus musculus, GN = Rpl27a, PE = 1, SV = 5	28	16595	11.12	11	2	4	1	4	0.29	0.28
JAK1_MOUSE	Tyrosine-protein kinase JAK1; OS = Mus musculus, GN = Jak1, PE = 1, SV = 1	28	133282	7.63	10	2	6	2	6	0.08	0.06
TBL1R_MOUSE	F-box-like/WD repeat-containing protein TBL1XR1; OS = Mus musculus, GN = Tbl1xr1, PE = 1, SV = 1	28	55626	5.33	4	1	2	1	2	0.1	0.08
PIPNA_MOUSE	Phosphatidylinositol transfer protein alpha isoform; OS = Mus musculus, GN = Pitpna, PE = 1, SV = 2	28	31873	5.97	2	1	2	1	2	0.13	0.14
GLGB_MOUSE	1,4-Alpha-glucan-branching enzyme; OS = Mus musculus, GN = Gbe1, PE = 1, SV = 1	28	80313	5.97	2	2	1	1	1	0.02	0.05
CY24B_MOUSE	Cytochrome b-245 heavy chain; OS = Mus musculus, GN = Cybb, PE = 1, SV = 1	27	65262	7.83	1	1	1	1	1	0.05	0.07
SNX7_MOUSE	Sorting nexin-7; OS = Mus musculus, GN = Snx7, PE = 1, SV = 1	27	44971	4.99	3	2	2	1	2	0.14	0.1
CSN8_MOUSE	COP9 signalosome complex subunit 8; OS = Mus musculus, GN = Cops8, PE = 1, SV = 1	27	23241	5.09	3	1	3	1	3	0.38	0.19
MTNA_MOUSE	Methylthioribose-1-phosphate isomerase; OS = Mus musculus, GN = Mri1, PE = 1, SV = 1	27	39386	5.6	3	2	3	2	3	0.25	0.23
SIR2_MOUSE	NAD-dependent protein deacetylase sirtuin-2; OS = Mus musculus, GN = Sirt2, PE = 1, SV = 2	27	43228	5.23	1	1	1	1	1	0.06	0.1
QCR7_MOUSE	Cytochrome b-c1 complex subunit 7 OS = Mus musculus, GN = Uqcrb, PE = 1, SV = 3	27	13519	9.1	2	1	2	1	2	0.21	0.35
ERBIN_MOUSE	Erbin; OS = Mus musculus, GN = Erbin, PE = 1, SV = 3	27	157150	5.46	4	1	4	1	4	0.05	0.03
TTL12_MOUSE	Tubulin–tyrosine ligase-like protein 12; OS = Mus musculus, GN = Ttll12, PE = 1, SV = 1	27	73996	5.39	3	1	2	1	2	0.07	0.06
FBLN3_MOUSE	EGF-containing fibulin-like extracellular matrix protein 1	27	54916	5.01	4	1	3	1	3	0.1	0.08
PYRD_MOUSE	Dihydroorotate dehydrogenase (quinone), mitochondrial	27	42674	9.56	1	1	1	1	1	0.06	0.1
COMD2_MOUSE	COMM domain-containing protein 2	26	22834	6.14	3	1	3	1	3	0.28	0.2
CBR1_MOUSE	Carbonyl reductase [NADPH] 1	26	30622	8.53	2	1	2	1	2	0.07	0.14
CTL1_MOUSE	Choline transporter-like protein 1	26	73018	9.04	4	1	4	1	4	0.06	0.06
CD47_MOUSE	Leukocyte surface antigen CD47	26	33076	8.93	2	1	2	1	2	0.14	0.13
DJC24_MOUSE	DnaJ homolog subfamily C member 24	26	22066	5.63	2	1	2	1	2	0.17	0.21
ACOD2_MOUSE	Acyl-CoA desaturase 2	26	40890	9.14	2	1	2	1	2	0.13	0.11
PROS_MOUSE	Vitamin K-dependent protein S	26	74886	5.61	8	1	5	1	5	0.11	0.06
TTYH2_MOUSE	Protein tweety homolog 2	26	58969	5.67	2	1	2	1	2	0.05	0.07
IF2P_MOUSE	Eukaryotic translation initiation factor 5B	26	137532	5.47	10	2	9	2	9	0.14	0.06
MCA3_MOUSE	Eukaryotic translation elongation factor 1 epsilon-1	26	19846	8.6	1	1	1	1	1	0.06	0.23
TM214_MOUSE	Transmembrane protein 214	26	76381	9.41	5	1	5	1	5	0.16	0.06
S10AA_MOUSE	Protein S100-A10	26	11179	6.27	4	1	1	1	1	0.27	0.44
ARAP1_MOUSE	Arf-GAP with Rho-GAP domain, ANK repeat and pH domain-containing protein 1	25	162174	5.81	2	1	2	1	2	0.03	0.03
LMF2_MOUSE	Lipase maturation factor 2	25	79947	9.99	1	1	1	1	1	0.02	0.05
OLR1_MOUSE	Oxidized low-density lipoprotein receptor 1	25	41617	7.55	2	1	2	1	2	0.09	0.11
UCRI_MOUSE	Cytochrome b-c1 complex subunit Rieske, mitochondrial	25	29349	8.91	2	2	1	1	1	0.09	0.15
MPPA_MOUSE	Mitochondrial-processing peptidase subunit alpha	25	58242	6.36	2	1	2	1	2	0.06	0.07
ARFG1_MOUSE	ADP-ribosylation factor GTPase-activating protein 1	25	45260	5.39	2	1	2	1	2	0.08	0.1
UBP24_MOUSE	Ubiquitin carboxyl-terminal hydrolase 24	25	293814	5.82	4	1	4	1	4	0.03	0.01
AIMP1_MOUSE	Aminoacyl tRNA synthase complex-interacting multifunctional protein 1	25	33976	8.57	3	1	2	1	2	0.15	0.13
STX17_MOUSE	Syntaxin-17	25	33201	6.28	2	1	2	1	2	0.15	0.13
TM165_MOUSE	Transmembrane protein 165	25	34768	6.97	7	1	3	1	3	0.25	0.13
ACOT2_MOUSE	Acyl-coenzyme A thioesterase 2, mitochondrial	25	49626	6.88	1	1	1	1	1	0.06	0.09
SPD2B_MOUSE	SH3 and PX domain-containing protein 2B	25	101454	8.78	3	1	3	1	3	0.07	0.04
VP13C_MOUSE	Vacuolar protein sorting-associated protein 13C	25	419824	6.37	10	1	8	1	8	0.04	0.01
LAP2B_MOUSE	Lamina-associated polypeptide 2, isoforms beta/delta/epsilon/gamma	25	50342	9.45	7	1	7	1	7	0.24	0.09
T106A_MOUSE	Transmembrane protein 106A	25	29091	7.07	5	1	2	1	2	0.16	0.15
NRP2_MOUSE	Neuropilin-2	24	104565	5.07	2	1	2	1	2	0.04	0.04
VISTA_MOUSE	V-type immunoglobulin domain-containing suppressor of T cell activation	24	33538	7.21	2	1	1	1	1	0.04	0.13
LYPA1_MOUSE	Acyl-protein thioesterase 1	24	24671	6.14	2	1	1	1	1	0.17	0.18
RIOK3_MOUSE	Serine/threonine-protein kinase RIO3	24	58667	5.47	4	1	3	1	3	0.09	0.07
RFA1_MOUSE	Replication protein A 70 kDa DNA-binding subunit	24	68994	8.13	5	1	4	1	4	0.11	0.06
SRRM1_MOUSE	Serine/arginine repetitive matrix protein 1	24	106798	11.87	8	1	6	1	6	0.12	0.04
GVIN1_MOUSE	Interferon-induced very large GTPase 1	24	280637	6.12	5	1	5	1	5	0.03	0.02
INO1_MOUSE	Inositol-3-phosphate synthase 1	24	60893	5.99	3	1	3	1	3	0.1	0.07
ADAS_MOUSE	Alkyldihydroxyacetonephosphate synthase, peroxisomal	24	71638	7.25	2	1	2	1	2	0.06	0.06
CASP3_MOUSE	Caspase-3	24	31454	6.45	3	1	3	1	3	0.18	0.14
RIDA_MOUSE	2-Iminobutanoate/2-iminopropanoate deaminase	24	14247	8.74	4	2	2	1	2	0.19	0.33
ARFG2_MOUSE	ADP-ribosylation factor GTPase-activating protein 2	24	56563	8.36	3	1	3	1	3	0.14	0.08
DHC24_MOUSE	Delta(24)-sterol reductase	24	60073	8.42	8	1	7	1	7	0.2	0.07
RT16_MOUSE	28S ribosomal protein S16, mitochondrial	24	15182	9.74	1	1	1	1	1	0.15	0.31
PTH_MOUSE	Probable peptidyl-tRNA hydrolase	24	22147	9.92	1	1	1	1	1	0.1	0.2
SWP70_MOUSE	Switch-associated protein 70	23	68953	5.78	2	1	2	1	2	0.07	0.06
VWA8_MOUSE	von Willebrand factor A domain-containing protein 8	23	213287	6.14	8	1	7	1	7	0.08	0.02
UBXN4_MOUSE	UBX domain-containing protein 4	23	56437	6.23	5	1	4	1	4	0.23	0.08
AEBP1_MOUSE	Adipocyte enhancer-binding protein 1	23	128284	5.02	6	1	5	1	5	0.09	0.03
GCR_MOUSE	Glucocorticoid receptor	23	85998	5.99	8	1	7	1	7	0.1	0.05
IAH1_MOUSE	Isoamyl acetate-hydrolyzing esterase 1 homolog	23	27956	5.34	3	1	2	1	2	0.12	0.16
VPS16_MOUSE	Vacuolar protein sorting-associated protein 16 homolog	22	94868	6.56	6	2	5	1	5	0.1	0.05
LY6C1_MOUSE	Lymphocyte antigen 6C1	22	14183	5.76	2	1	2	1	2	0.29	0.33
BCAT1_MOUSE	Branched-chain-amino-acid aminotransferase, cytosolic	22	42764	5.25	3	1	3	1	3	0.12	0.1
E2AK2_MOUSE	Interferon-induced, double-stranded RNA-activated protein kinase	22	58243	8.76	1	1	1	1	1	0.05	0.07
FLII_MOUSE	Protein flightless-1 homolog	21	144712	5.75	9	1	9	1	9	0.1	0.03
CHCH2_MOUSE	Coiled-coil-helix-coiled-coil-helix domain-containing protein 2	21	15651	9.78	2	1	2	1	2	0.24	0.3
TNPO2_MOUSE	Transportin-2	21	100391	4.85	7	1	6	1	4	0.14	0.04
UBFD1_MOUSE	Ubiquitin domain-containing protein UBFD1	21	40118	8.98	7	1	4	1	4	0.2	0.11
WWOX_MOUSE	WW domain-containing oxidoreductase	21	46483	6.54	2	1	2	1	2	0.11	0.09
DYR_MOUSE	Dihydrofolate reductase	21	21592	8.56	3	1	2	1	2	0.23	0.21
ARHG1_MOUSE	Rho guanine nucleotide exchange factor 1	21	102741	5.43	3	1	3	1	3	0.06	0.04
ATX2L_MOUSE	Ataxin-2-like protein	20	110580	8.94	2	1	2	1	2	0.05	0.04
PSD10_MOUSE	26S proteasome non-ATPase regulatory subunit 10	20	25068	5.68	1	1	1	1	1	0.06	0.18
TMF1_MOUSE	TATA element modulatory factor	20	121729	4.83	5	2	3	1	3	0.06	0.03
ANFY1_MOUSE	Rabankyrin-5	20	128571	5.58	5	2	4	1	4	0.07	0.03
MAGD1_MOUSE	Melanoma-associated antigen D1	20	85617	7.01	5	1	4	1	4	0.1	0.05
YIF1A_MOUSE	Protein YIF1A	19	32114	9.11	1	1	1	1	1	0.05	0.14
TR10B_MOUSE	Tumor necrosis factor receptor superfamily member 10B	19	42138	6.75	4	1	3	1	3	0.13	0.1
QCR8_MOUSE	Cytochrome b-c1 complex subunit 8	19	9762	10.26	1	1	1	1	1	0.28	0.51
ATP5J_MOUSE	ATP synthase-coupling factor 6, mitochondrial	18	12489	9.36	6	1	4	1	4	0.4	0.38
YAP1_MOUSE	Transcriptional coactivator YAP1	18	52351	4.96	1	1	1	1	1	0.05	0.08
PARP9_MOUSE	Poly [ADP-ribose] polymerase 9	17	96597	6.68	3	1	3	1	3	0.09	0.04
MTCH2_MOUSE	Mitochondrial carrier homolog 2	17	33477	8.59	1	1	1	1	1	0.07	0.13
NXN_MOUSE	Nucleoredoxin	17	48314	4.84	1	1	1	1	1	0.06	0.09

^a^Protein score is calculated from the score of the peptide attributed to the protein. ^b^pI is (predicted) isoelectric point. ^c^Number of matches is spectrum number matched to protein ^#1^. ^d^Number of significant matches is spectrum number that matches protein and exceeds the identification criteria. ^e^Number of sequences is number of peptides matched to protein ^#2^. ^f^Number of significant sequences is number of peptides exceeding the identification criteria matched to proteins. ^g^Number of unique sequences is a unique ^#3^ number of peptides matched to proteins. ^h^Sequence coverage is the ratio of the total number of matched peptide residues to the total length of the protein. ^i^Exponentially modified protein abundance index (http://www.matrixscience.com/help/quant_empai_help.html). ^#1^When multiple spectra are matched to the same peptide, the way of counting into one is called "peptide number". ^#2^When multiple spectra are matched to the same peptide, the method of counting is called "spectrum number". ^#3^"Unique" is a peptide that is not matched to other proteins and has been assigned only to the relevant protein.

**Table 2:** Identification of endogenous proteins contained in mMSC-AT_P3 (cells passaged 3 times).

UniProt/Swiss-Prot ID	Description	Protein score^a^	Protein mass (kDa)	pI^b^	Num. of matches^c^	Num. of significant matches^d^	Num. of sequences^e^	Num. of significant sequences^f^	Num. of unique sequences^g^	Sequence coverage^h^	emPAI^i^
MYH9_MOUSE	Myosin-9	3652	226232	5.54	238	130	81	52	74	0.52	1.93
MYH10_MOUSE	Myosin-10	793	228855	5.43	66	31	25	13	18	0.19	0.32
SERPH_MOUSE	Serpin H1	3042	46504	8.88	217	141	22	21	22	0.59	7.57
G3P_MOUSE	Glyceraldehyde-3-phosphate dehydrogenase	3019	35787	8.44	240	131	15	12	9	0.67	8.1
S10AB_MOUSE	Protein S100-A11	2948	11075	5.28	127	109	5	4	3	0.66	37.69
CLH1_MOUSE	Clathrin heavy chain 1	2893	191435	5.48	163	110	62	45	62	0.57	1.92
ACTN1_MOUSE	Alpha-actinin-1	2445	103004	5.23	136	82	42	31	27	0.65	2.98
ACTN4_MOUSE	Alpha-actinin-4	1377	104911	5.25	111	60	36	24	21	0.56	1.82
KPYM_MOUSE	Pyruvate kinase PKM	2269	57808	7.18	136	93	33	26	14	0.69	7.7
HS90A_MOUSE	Heat shock protein HSP 90-alpha	1754	84735	4.93	181	93	28	22	21	0.52	2.79
ENPL_MOUSE	Endoplasmin	1610	92418	4.74	101	64	34	24	16	0.48	2.39
TRAP1_MOUSE	Heat shock protein 75 kDa, mitochondrial	123	80159	6.25	6	6	4	4	4	0.11	0.23
ENOA_MOUSE	Alpha-enolase	2147	47111	6.37	163	78	20	14	7	0.69	4.37
FLNA_MOUSE	Filamin-A	2029	281046	5.68	241	136	64	42	61	0.43	0.93
FLNB_MOUSE	Filamin-B	1355	277651	5.46	119	68	56	40	52	0.43	0.86
FLNC_MOUSE	Filamin-C	351	290937	5.63	66	26	36	16	31	0.24	0.3
GRP75_MOUSE	Stress-70 protein, mitochondrial	961	73416	5.81	123	47	16	11	16	0.36	0.98
UBA1_MOUSE	Ubiquitin-like modifier-activating enzyme 1	1729	117734	5.43	87	64	29	22	29	0.58	1.52
TLN1_MOUSE	Talin-1	1723	269653	5.84	122	71	43	30	32	0.35	0.64
FAS_MOUSE	Fatty acid synthase	1679	272257	6.13	109	68	54	36	54	0.42	0.77
EF2_MOUSE	Elongation factor 2	1674	95253	6.41	114	76	31	25	31	0.61	2.27
VIME_MOUSE	Vimentin	1667	53655	5.06	152	77	37	26	37	0.67	8.53
ASSY_MOUSE	Argininosuccinate synthase	1574	46555	8.36	96	68	22	15	18	0.66	6.84
ANXA2_MOUSE	Annexin A2	1565	38652	7.55	126	98	14	11	14	0.54	2.27
VDAC1_MOUSE	Voltage-dependent anion-selective channel protein 1	1559	32331	8.55	161	108	13	8	13	0.65	2.18
VDAC2_MOUSE	Voltage-dependent anion-selective channel protein 2	312	31713	7.44	76	23	8	6	8	0.42	1.19
DYHC1_MOUSE	Cytoplasmic dynein 1 heavy chain 1	1401	531710	6.03	112	64	65	36	65	0.26	0.34
CH60_MOUSE	60 kDa heat shock protein, mitochondrial	1376	60917	5.91	76	42	22	18	22	0.54	2.67
PDIA1_MOUSE	Protein disulfide-isomerase	1325	57023	4.77	92	45	19	15	19	0.41	1.99
PLEC_MOUSE	Plectin	1320	533861	5.74	113	55	74	36	74	0.26	0.33
TKT_MOUSE	Transketolase	1319	67588	7.23	83	61	22	20	22	0.62	2.66
TBB6_MOUSE	Tubulin beta-6 chain	846	50058	4.8	83	31	19	13	7	0.61	2.48
ANXA5_MOUSE	Annexin A5	1219	35730	4.83	83	50	17	14	15	0.67	6.21
CATB_MOUSE	Cathepsin B	1206	37256	5.57	37	31	7	5	7	0.45	1.44
GELS_MOUSE	Gelsolin	1190	85888	5.83	68	48	19	15	13	0.56	1.29
GELS_HORSE	Gelsolin	546	80777	5.58	33	24	9	6	3	0.28	0.51
IMB1_MOUSE	Importin subunit beta-1	1155	97122	4.68	71	53	29	22	29	0.61	2.2
FPPS_MOUSE	Farnesyl pyrophosphate synthase	1095	40556	5.49	44	31	14	11	14	0.68	2.43
PSMD2_MOUSE	26S proteasome non-ATPase regulatory subunit 2	1057	100139	5.06	51	39	19	15	19	0.44	0.95
TCPA_MOUSE	T-complex protein 1 subunit alpha	1055	60411	5.82	44	31	16	11	16	0.54	1.29
ESTD_MOUSE	S-Formylglutathione hydrolase	1044	31299	6.7	53	36	15	11	15	0.79	4.6
ATPA_MOUSE	ATP synthase subunit alpha, mitochondrial	1041	59716	9.22	59	42	16	14	4	0.49	2.06
IQGA1_MOUSE	Ras GTPase-activating-like protein IQGAP1	1034	188624	6.07	67	37	35	19	35	0.36	0.56
WDR1_MOUSE	WD repeat-containing protein 1	1014	66365	6.11	64	42	19	15	19	0.65	1.57
TCPE_MOUSE	T-complex protein 1 subunit epsilon	1002	59586	5.72	51	33	15	9	15	0.55	1.16
SPB6_MOUSE	Serpin B6	988	42571	5.53	46	35	13	11	13	0.56	2.92
ACLY_MOUSE	ATP-citrate synthase	961	119651	7.13	64	44	26	20	26	0.42	1.08
LRP1_MOUSE	Prolow-density lipoprotein receptor-related protein 1	939	504411	5.14	76	46	37	25	37	0.16	0.27
CAN2_MOUSE	Calpain-2 catalytic subunit	937	79822	4.86	55	37	18	15	17	0.53	1.31
ALDOA_MOUSE	Fructose-bisphosphate aldolase A	923	39331	8.31	75	49	15	13	15	0.62	3.89
SPTB2_MOUSE	Spectrin beta chain, nonerythrocytic 1	900	274052	5.4	74	42	33	21	33	0.24	0.38
LDHA_MOUSE	L-Lactate dehydrogenase A chain	888	36475	7.62	64	43	17	12	13	0.68	3.92
LDHB_MOUSE	L-Lactate dehydrogenase B chain	144	36549	5.7	9	6	3	3	3	0.19	0.41
1433B_MOUSE	14-3-3 protein beta/alpha	483	28069	4.77	30	19	8	5	4	0.5	1.81
TCPZ_MOUSE	T-complex protein 1 subunit zeta	854	57968	6.63	51	37	13	10	13	0.41	1.05
COF1_MOUSE	Cofilin-1	795	18548	8.22	57	34	12	9	11	0.68	6.37
COF2_MOUSE	Cofilin-2	363	18698	7.66	17	10	5	5	4	0.46	2.01
SYAC_MOUSE	Alanine–tRNA ligase, cytoplasmic	785	106841	5.45	46	33	19	13	19	0.35	0.66
PLST_MOUSE	Plastin-3	784	70697	5.42	56	35	21	15	21	0.54	1.42
ANXA1_MOUSE	Annexin A1	774	38710	6.97	49	30	11	9	11	0.46	1.92
THIO_MOUSE	Thioredoxin	767	11668	4.8	33	19	7	3	7	0.78	1.85
PRDX6_MOUSE	Peroxiredoxin-6	751	24855	5.71	40	28	11	8	11	0.61	2.78
SPTN1_MOUSE	Spectrin alpha chain, nonerythrocytic 1	749	284422	5.2	77	34	42	24	42	0.27	0.42
LEG1_MOUSE	Galectin-1	735	14856	5.32	56	36	11	9	11	0.82	26.15
TCPH_MOUSE	T-complex protein 1 subunit eta	734	59614	7.95	38	26	16	12	16	0.5	1.48
HNRPF_MOUSE	Heterogeneous nuclear ribonucleoprotein F	710	45701	5.31	33	25	9	7	3	0.46	1.27
EF1G_MOUSE	Elongation factor 1-gamma	689	50029	6.31	44	28	15	13	15	0.57	1.95
PDIA3_MOUSE	Protein disulfide-isomerase A3	682	56643	5.88	64	37	23	16	14	0.54	2.5
TENA_MOUSE	Tenascin	679	231659	4.77	33	23	17	11	17	0.16	0.22
ATPB_MOUSE	ATP synthase subunit beta, mitochondrial	676	56265	5.19	69	33	23	15	7	0.74	2.27
RCN2_MOUSE	Reticulocalbin-2	663	37248	4.28	23	20	9	8	9	0.46	1.44
TCPB_MOUSE	T-complex protein 1 subunit beta	654	57441	5.97	32	19	17	12	17	0.49	1.39
LYAG_MOUSE	Lysosomal alpha-glucosidase	630	106180	5.53	45	34	16	13	16	0.34	0.81
CKAP4_MOUSE	Cytoskeleton-associated protein 4	626	63654	5.46	41	23	14	7	14	0.38	0.58
DPYL2_MOUSE	Dihydropyrimidinase-related protein 2	626	62239	5.95	47	26	24	13	21	0.63	1.39
DPYL3_MOUSE	Dihydropyrimidinase-related protein 3	602	61897	6.04	26	19	15	8	12	0.57	0.96
ESYT1_MOUSE	Extended synaptotagmin-1	621	121478	5.63	25	21	9	7	9	0.19	0.27
NEDD4_MOUSE	E3 ubiquitin-protein ligase NEDD4	611	102642	5.12	40	26	17	13	17	0.31	0.77
SYVC_MOUSE	Valine–tRNA ligase	604	140127	7.9	37	26	14	9	14	0.24	0.35
GSTO1_MOUSE	Glutathione S-transferase omega-1	600	27480	6.92	50	30	13	7	13	0.65	2.87
SEPT2_MOUSE	Septin-2	599	41499	6.1	24	19	9	8	8	0.38	1.46
6PGD_MOUSE	6-Phosphogluconate dehydrogenase, decarboxylating	599	53213	6.81	50	32	18	12	18	0.63	2
RPN1_MOUSE	Dolichyl-diphosphooligosaccharide–protein glycosyltransferase subunit 1	597	68486	6.02	35	22	15	11	15	0.4	0.96
NUCL_MOUSE	Nucleolin	588	76677	4.69	37	22	15	9	15	0.27	0.63
GDIB_MOUSE	Rab GDP dissociation inhibitor beta	583	50505	5.93	40	26	13	10	11	0.45	1.48
TPM4_MOUSE	Tropomyosin alpha-4 chain	581	28450	4.65	51	29	10	9	10	0.44	3.3
PPCE_MOUSE	Prolyl endopeptidase	572	80700	5.44	30	21	12	10	12	0.29	0.68
NCPR_MOUSE	NADPH–cytochrome P450 reductase	570	76995	5.34	31	19	13	8	13	0.34	0.54
CISY_MOUSE	Citrate synthase, mitochondrial	563	51703	8.72	28	24	12	11	12	0.44	2.09
DDX3X_MOUSE	ATP-dependent RNA helicase DDX3X	555	73056	6.73	28	21	12	8	12	0.32	0.58
P5CS_MOUSE	Delta-1-pyrroline-5-carboxylate synthase	553	87212	7.18	26	18	10	5	10	0.2	0.27
MBB1A_MOUSE	Myb-binding protein 1A	547	151942	9.08	35	21	17	10	17	0.23	0.32
EF1B_MOUSE	Elongation factor 1-beta	546	24678	4.53	29	24	9	9	9	0.57	3.53
UGGG1_MOUSE	UDP-glucose:glycoprotein glucosyltransferase 1	545	176323	5.4	49	29	23	14	23	0.29	0.46
RPN2_MOUSE	Dolichyl-diphosphooligosaccharide–protein glycosyltransferase subunit 2	542	69020	5.54	27	19	12	11	12	0.37	0.94
HS105_MOUSE	Heat shock protein 105 kDa	541	96346	5.39	27	17	16	11	15	0.29	0.61
HSP74_MOUSE	Heat shock 70 kDa protein 4	440	94073	5.15	26	20	17	16	2	0.3	1.04
PROF1_MOUSE	Profilin-1	536	14948	8.46	79	30	8	7	8	0.61	19.18
GANAB_MOUSE	Neutral alpha-glucosidase AB	523	106844	5.67	49	27	20	15	20	0.46	0.8
THIC_MOUSE	Acetyl-CoA acetyltransferase, cytosolic	523	41271	7.16	30	20	8	6	8	0.5	0.83
ANXA6_MOUSE	Annexin A6	518	75837	5.34	39	20	19	10	19	0.4	0.73
P4HA1_MOUSE	Prolyl 4-hydroxylase subunit alpha-1	510	60872	5.62	32	26	13	10	13	0.44	1.13
NDKB_MOUSE	Nucleoside diphosphate kinase B	508	17352	6.97	31	19	7	4	4	0.63	2.27
KAD1_MOUSE	Adenylate kinase isoenzyme 1	499	21526	5.67	26	19	8	5	8	0.57	1.61
SAP_MOUSE	Prosaposin	497	61381	5.07	48	30	11	5	11	0.31	0.72
PGH1_MOUSE	Prostaglandin G/H synthase 1	491	68998	6.36	29	21	10	8	10	0.21	0.72
HMCS1_MOUSE	Hydroxymethylglutaryl-CoA synthase, cytoplasmic	487	57532	5.65	31	22	15	13	15	0.49	1.76
CAP1_MOUSE	Adenylyl cyclase-associated protein 1	478	51532	7.16	39	22	11	8	11	0.36	0.91
MK01_MOUSE	Mitogen-activated protein kinase 1	474	41249	6.5	21	16	7	5	6	0.44	0.65
ARF4_MOUSE	ADP-ribosylation factor 4	470	20384	6.59	35	20	12	8	6	0.8	6.52
2AAA_MOUSE	Serine/threonine-protein phosphatase 2A 65 kDa regulatory subunit A alpha isoform	470	65281	5	22	15	14	11	14	0.42	1.02
CPNS1_MOUSE	Calpain small subunit 1	469	28445	5.41	24	18	6	5	6	0.39	1.4
FSCN1_MOUSE	Fascin	463	54474	6.44	37	22	14	10	14	0.51	1.32
PDIA4_MOUSE	Protein disulfide-isomerase A4	461	71938	5.16	32	20	15	10	15	0.39	0.79
TSP1_MOUSE	Thrombospondin-1	460	129564	4.72	33	15	16	8	15	0.19	0.29
G6PI_MOUSE	Glucose-6-phosphate isomerase	459	62727	8.14	35	17	15	9	15	0.47	1.08
NP1L1_MOUSE	Nucleosome assembly protein 1-like 1	458	45317	4.36	30	19	11	9	10	0.47	1.51
ADT1_MOUSE	ADP/ATP translocase 1	413	32883	9.73	25	19	11	9	5	0.49	3.01
IPO5_MOUSE	Importin-5	449	123511	4.82	38	24	22	13	22	0.37	0.61
RINI_MOUSE	Ribonuclease inhibitor	440	49784	4.69	21	16	12	9	12	0.55	1.12
AT1A1_MOUSE	Sodium/potassium-transporting ATPase subunit alpha-1	436	112910	5.3	19	15	12	10	12	0.2	0.45
GFPT1_MOUSE	Glutamine–fructose-6-phosphate aminotransferase [isomerizing] 1	433	78489	6.39	19	14	11	7	11	0.27	0.45
PSA_MOUSE	Puromycin-sensitive aminopeptidase	432	103260	5.61	19	12	7	3	7	0.17	0.13
PDIA6_MOUSE	Protein disulfide-isomerase A6	424	48070	5	36	26	14	11	14	0.48	1.59
DEST_MOUSE	Destrin	421	18509	8.14	26	14	9	7	9	0.54	4.9
MYOF_MOUSE	Myoferlin	421	233177	5.83	29	19	19	13	19	0.15	0.31
ALDH2_MOUSE	Aldehyde dehydrogenase, mitochondrial	419	56502	7.53	28	16	10	7	10	0.4	0.8
HNRPU_MOUSE	Heterogeneous nuclear ribonucleoprotein U	417	87863	5.92	32	20	13	9	13	0.27	0.53
ANXA4_MOUSE	Annexin A4	415	35893	5.43	14	10	8	6	8	0.36	1
PDC6I_MOUSE	Programmed cell death 6-interacting protein	414	95964	6.15	25	17	14	8	14	0.28	0.48
MARCS_MOUSE	Myristoylated alanine-rich C-kinase substrate	413	29644	4.34	65	43	2	1	2	0.15	0.15
CALU_MOUSE	Calumenin	308	37041	4.49	29	16	9	8	3	0.44	1.74
VIGLN_MOUSE	Vigilin	410	141655	6.43	32	18	19	10	19	0.24	0.34
FBLN2_MOUSE	Fibulin-2	409	131746	4.58	20	15	12	9	12	0.18	0.33
OAT_MOUSE	Ornithine aminotransferase, mitochondrial	408	48324	6.19	18	12	9	6	9	0.37	0.68
TPP1_MOUSE	Tripeptidyl-peptidase 1	404	61304	6.1	9	8	2	2	2	0.1	0.15
RL9_MOUSE	60S ribosomal protein L9	398	21868	9.96	19	15	7	5	7	0.49	2.1
FLII_MOUSE	Protein flightless-1 homolog	397	144712	5.75	27	15	15	8	15	0.28	0.3
UGDH_MOUSE	UDP-glucose 6-dehydrogenase	397	54797	7.49	35	21	19	13	19	0.6	1.69
ECHA_MOUSE	Trifunctional enzyme subunit alpha, mitochondrial	396	82617	9.24	19	13	9	6	9	0.25	0.35
PUR2_MOUSE	Trifunctional purine biosynthetic protein adenosine-3	395	107436	6.25	24	14	10	5	10	0.22	0.31
PPIA_MOUSE	Peptidyl-prolyl cis-trans isomerase A	393	17960	7.74	31	21	9	8	9	0.65	8.83
6PGL_MOUSE	6-Phosphogluconolactonase	389	27237	5.55	11	8	5	4	5	0.39	0.84
CAND1_MOUSE	Cullin-associated NEDD8-dissociated protein 1	385	136245	5.52	46	27	16	10	16	0.3	0.4
GARS_MOUSE	Glycine–tRNA ligase	385	81826	6.24	29	17	15	10	15	0.42	0.67
SND1_MOUSE	Staphylococcal nuclease domain-containing protein 1	381	102025	7.08	39	20	20	13	20	0.4	0.78
ESYT2_MOUSE	Extended synaptotagmin-2	380	94081	7.63	22	14	9	6	9	0.17	0.31
IDHC_MOUSE	Isocitrate dehydrogenase [NADP] cytoplasmic	374	46644	6.73	30	16	17	9	16	0.54	1.23
COPB_MOUSE	Coatomer subunit beta	371	106998	5.69	35	19	16	12	16	0.31	0.6
CNPY2_MOUSE	Protein canopy homolog 2	371	20754	4.95	5	5	2	2	2	0.19	0.49
MYADM_MOUSE	Myeloid-associated differentiation marker	371	35261	8.69	32	7	3	2	3	0.14	0.27
SYIC_MOUSE	Isoleucine–tRNA ligase, cytoplasmic	368	144179	6.14	33	17	13	8	13	0.19	0.26
DHE3_MOUSE	Glutamate dehydrogenase 1, mitochondrial	366	61298	8.05	26	14	12	8	12	0.28	0.85
CLIC1_MOUSE	Chloride intracellular channel protein 1	363	26996	5.09	29	19	10	8	10	0.58	2.98
RCN3_MOUSE	Reticulocalbin-3	362	37978	4.74	13	7	4	3	4	0.31	0.39
FINC_MOUSE	Fibronectin	361	272368	5.39	31	15	14	8	14	0.11	0.13
ADK_MOUSE	Adenosine kinase	360	40123	5.84	18	15	7	6	5	0.4	0.86
DHB4_MOUSE	Peroxisomal multifunctional enzyme type 2	359	79432	8.76	12	8	5	2	4	0.11	0.11
CALX_MOUSE	Calnexin	357	67236	4.5	32	19	14	10	14	0.38	0.86
RLA0_MOUSE	60S acidic ribosomal protein P0	355	34195	5.91	26	19	11	7	11	0.51	1.64
PYRG2_MOUSE	CTP synthase 2	190	65473	6.05	12	7	7	3	6	0.27	0.21
CATZ_MOUSE	Cathepsin Z	353	33974	6.13	16	11	3	1	3	0.23	0.28
GNAI2_MOUSE	Guanine nucleotide-binding protein G(i) subunit alpha-2	352	40463	5.28	17	11	9	6	9	0.36	0.85
CH10_MOUSE	10 kDa heat shock protein, mitochondrial	352	10956	7.93	30	18	5	2	5	0.39	1.09
VATB2_MOUSE	V-type proton ATPase subunit B, brain isoform	350	56515	5.57	20	12	10	7	10	0.41	0.68
MDHM_MOUSE	Malate dehydrogenase, mitochondrial	346	35589	8.93	37	17	10	8	10	0.43	1.54
MYL9_MOUSE	Myosin regulatory light polypeptide 9	192	19841	4.8	15	7	7	5	4	0.62	1.82
ADHX_MOUSE	Alcohol dehydrogenase class-3	341	39522	6.97	11	9	6	5	6	0.4	0.69
APT_MOUSE	Adenine phosphoribosyltransferase	338	19712	6.31	20	10	7	5	7	0.56	1.84
IPO4_MOUSE	Importin-4	338	119198	4.92	24	17	12	8	12	0.21	0.32
AATM_MOUSE	Aspartate aminotransferase, mitochondrial	336	47381	9.13	26	14	11	6	11	0.41	0.69
SC31A_MOUSE	Protein transport protein Sec31A	334	133486	6.3	24	18	13	10	13	0.21	0.37
MAOX_MOUSE	NADP-dependent malic enzyme	329	63913	7.16	35	18	13	10	13	0.47	0.92
NDKA_MOUSE	Nucleoside diphosphate kinase A	324	17197	6.84	23	16	5	2	2	0.47	1.05
TAGL2_MOUSE	Transgelin-2	323	22381	8.39	18	11	7	6	7	0.49	2.63
PPAC_MOUSE	Low molecular weight phosphotyrosine protein phosphatase	323	18180	6.3	9	7	5	3	5	0.44	0.97
P4HA2_MOUSE	Prolyl 4-hydroxylase subunit alpha-2	322	60964	5.55	26	19	9	8	9	0.27	0.73
IF4G1_MOUSE	Eukaryotic translation initiation factor 4 gamma 1	322	175967	5.3	24	16	11	9	11	0.11	0.27
MRC2_MOUSE	C-type mannose receptor 2	321	166968	5.65	32	16	17	9	17	0.21	0.29
DCTN1_MOUSE	Dynactin subunit 1	319	141588	5.66	14	10	6	3	6	0.1	0.13
DPYL1_MOUSE	Dihydropyrimidinase-related protein 1	317	62129	6.63	9	5	6	2	3	0.17	0.14
MVD1_MOUSE	Diphosphomevalonate decarboxylase	315	44044	5.89	11	8	6	5	6	0.34	0.6
PUR9_MOUSE	Bifunctional purine biosynthesis protein PURH	315	64177	6.3	19	11	11	7	8	0.31	0.58
SNX9_MOUSE	Sorting nexin-9	313	66504	5.35	16	12	8	6	8	0.26	0.46
AR6P1_MOUSE	ADP-ribosylation factor-like protein 6-interacting protein 1	312	23421	9.38	6	6	1	1	1	0.14	0.42
XPO2_MOUSE	Exportin-2	312	110384	5.52	20	10	12	6	12	0.26	0.26
ANXA3_MOUSE	Annexin A3	312	36362	5.5	17	10	6	4	6	0.34	0.58
OST48_MOUSE	Dolichyl-diphosphooligosaccharide–protein glycosyltransferase 48 kDa subunit	310	48997	5.52	21	15	9	8	9	0.41	1.34
TCPD_MOUSE	T-complex protein 1 subunit delta	310	58030	8.24	15	9	9	6	9	0.29	0.54
PLOD3_MOUSE	Procollagen-lysine,2-oxoglutarate 5-dioxygenase 3	309	84869	5.81	29	18	17	10	17	0.41	0.64
RL5_MOUSE	60S ribosomal protein L5	308	34379	9.78	14	8	5	4	5	0.33	0.83
CATD_MOUSE	Cathepsin D	308	44925	6.71	39	18	10	8	10	0.58	2.04
SC22B_MOUSE	Vesicle-trafficking protein SEC22b	307	24725	8.67	18	12	7	4	7	0.6	1.3
PLOD1_MOUSE	Procollagen-lysine,2-oxoglutarate 5-dioxygenase 1	306	83542	6.08	17	10	10	6	10	0.24	0.42
TCTP_MOUSE	Translationally-controlled tumor protein	306	19450	4.76	28	14	6	4	6	0.48	1.89
TCPG_MOUSE	T-complex protein 1 subunit gamma	304	60591	6.28	17	12	11	8	11	0.37	0.86
SC23A_MOUSE	Protein transport protein Sec23A	303	86106	6.64	10	7	6	3	6	0.16	0.16
SAHH_MOUSE	Adenosylhomocysteinase	302	47657	6.08	28	15	11	8	11	0.42	1.01
TCPQ_MOUSE	T-complex protein 1 subunit theta	298	59518	5.44	17	8	14	6	14	0.39	0.52
PSMD1_MOUSE	26S proteasome non-ATPase regulatory subunit 1	298	105663	5.25	37	15	15	8	15	0.32	0.37
COCA1_MOUSE	Collagen alpha-1(XII) chain	296	340004	5.47	24	7	14	6	14	0.09	0.08
GCN1_MOUSE	eIF-2-alpha kinase activator GCN1	295	292834	7.14	22	7	15	4	15	0.1	0.06
USO1_MOUSE	General vesicular transport factor p115	295	106917	4.85	20	15	10	5	10	0.22	0.22
UAP1L_MOUSE	UDP-N-acetylhexosamine pyrophosphorylase-like protein 1	295	56578	5.27	20	15	7	6	6	0.21	0.56
UAP1_MOUSE	UDP-N-acetylhexosamine pyrophosphorylase	93	58572	6.04	10	4	5	2	4	0.12	0.15
PTBP1_MOUSE	Polypyrimidine tract-binding protein 1	289	56443	8.47	19	11	9	8	9	0.39	0.81
EIF3A_MOUSE	Eukaryotic translation initiation factor 3 subunit A	288	161838	6.38	18	11	10	5	10	0.12	0.14
GSTP1_MOUSE	Glutathione S-transferase P 1	286	23594	7.68	31	12	8	5	8	0.74	2.4
SERA_MOUSE	D-3-Phosphoglycerate dehydrogenase	279	56549	6.12	15	10	9	7	9	0.32	0.8
GDIR1_MOUSE	Rho GDP-dissociation inhibitor 1	279	23393	5.12	29	18	9	6	9	0.42	1.88
VINC_MOUSE	Vinculin	278	116644	5.77	27	14	15	10	15	0.26	0.43
PNPH_MOUSE	Purine nucleoside phosphorylase	277	32256	5.78	12	8	5	4	5	0.37	0.67
PSMD7_MOUSE	26S proteasome non-ATPase regulatory subunit 7	275	36517	6.29	17	12	8	6	8	0.54	0.98
LRC59_MOUSE	Leucine-rich repeat-containing protein 59	274	34856	9.57	14	9	6	2	6	0.32	0.27
UBP14_MOUSE	Ubiquitin carboxyl-terminal hydrolase 14	273	55966	5.15	14	11	6	4	6	0.25	0.35
CNN3_MOUSE	Calponin-3	272	36406	5.46	20	12	8	6	8	0.45	1.22
SDHA_MOUSE	Succinate dehydrogenase [ubiquinone] flavoprotein subunit, mitochondrial	270	72539	7.06	16	11	7	4	7	0.27	0.26
VAT1_MOUSE	Synaptic vesicle membrane protein VAT-1 homolog	269	43069	5.95	29	12	10	7	10	0.38	0.97
RLA2_MOUSE	60S acidic ribosomal protein P2	267	11644	4.42	17	10	5	3	5	0.7	1.85
ARLY_MOUSE	Argininosuccinate lyase	264	51707	6.48	10	8	5	3	5	0.22	0.38
DDX1_MOUSE	ATP-dependent RNA helicase DDX1	263	82448	6.8	18	9	12	6	12	0.29	0.36
PYR1_MOUSE	CAD protein	262	243084	6	22	8	14	6	14	0.13	0.11
AATC_MOUSE	Aspartate aminotransferase, cytoplasmic	260	46219	6.68	16	7	7	4	7	0.3	0.43
STIP1_MOUSE	Stress-induced-phosphoprotein 1	259	62542	6.4	18	8	9	6	9	0.31	0.49
ZYX_MOUSE	Zyxin	257	60507	5.99	15	11	5	3	5	0.17	0.32
RL3_MOUSE	60S ribosomal protein L3	257	46081	10.22	18	10	7	5	7	0.25	0.57
CO1A1_MOUSE	Collagen alpha-1(I) chain	253	137948	5.65	19	14	9	6	8	0.12	0.24
PCOC1_MOUSE	Procollagen C-endopeptidase enhancer 1	253	50136	8.73	11	8	7	4	7	0.33	0.51
RL6_MOUSE	60S ribosomal protein L6	250	33489	10.69	13	9	8	5	8	0.25	0.86
PP1A_MOUSE	Serine/threonine-protein phosphatase PP1-alpha catalytic subunit	249	37516	5.94	19	13	8	7	4	0.38	1.17
FUMH_MOUSE	Fumarate hydratase, mitochondrial	246	54322	9.12	20	11	8	5	8	0.35	0.59
PCBP2_MOUSE	Poly(rC)-binding protein 2	154	38197	6.33	25	11	7	4	5	0.33	0.55
LKHA4_MOUSE	Leukotriene A-4 hydrolase	246	69007	5.98	15	10	9	4	9	0.22	0.27
TOM40_MOUSE	Mitochondrial import receptor subunit TOM40 homolog	244	37871	7.64	18	10	9	6	9	0.48	0.93
SH3L3_MOUSE	SH3 domain-binding glutamic acid-rich-like protein 3	243	10470	5.02	15	10	3	1	3	0.65	1.17
SYNC_MOUSE	Asparagine–tRNA ligase, cytoplasmic	242	64238	5.62	22	12	15	6	15	0.38	0.48
PUR6_MOUSE	Multifunctional protein ADE2	242	46976	6.94	23	10	10	5	10	0.44	0.7
AL7A1_MOUSE	Alpha-aminoadipic semialdehyde dehydrogenase	242	58824	7.16	13	9	6	5	6	0.27	0.43
CP51A_MOUSE	Lanosterol 14-alpha demethylase	242	56739	8.6	16	8	9	3	9	0.34	0.25
SYSC_MOUSE	Serine–tRNA ligase, cytoplasmic	241	58352	5.95	19	9	10	4	10	0.35	0.33
NDUS1_MOUSE	NADH-ubiquinone oxidoreductase 75 kDa subunit, mitochondrial	239	79726	5.51	15	10	9	4	9	0.22	0.3
EIF3H_MOUSE	Eukaryotic translation initiation factor 3 subunit H	238	39807	6.2	22	14	11	7	11	0.5	1.3
PSB6_MOUSE	Proteasome subunit beta type-6	237	25362	4.97	7	7	4	4	4	0.47	0.92
PSA6_MOUSE	Proteasome subunit alpha type-6	235	27355	6.34	9	8	3	3	3	0.29	0.58
AMPL_MOUSE	Cytosol aminopeptidase	234	56106	7.62	17	9	11	6	11	0.34	0.68
TXND5_MOUSE	Thioredoxin domain-containing protein 5	232	46386	5.51	13	7	7	4	7	0.29	0.43
TAGL_MOUSE	Transgelin	231	22561	8.85	19	13	8	6	8	0.49	2
AIMP2_MOUSE	Aminoacyl tRNA synthase complex-interacting multifunctional protein 2	229	35355	7.7	15	9	6	3	6	0.42	0.42
RET1_MOUSE	Retinol-binding protein 1	229	15836	5.1	9	6	4	3	4	0.44	1.17
IPYR_MOUSE	Inorganic pyrophosphatase	229	32646	5.37	14	7	7	3	7	0.31	0.46
GSTM2_MOUSE	Glutathione S-transferase Mu 2	228	25700	6.9	14	9	7	6	6	0.39	1.62
GSTM1_MOUSE	Glutathione S-transferase Mu 1	197	25953	7.71	14	8	5	4	4	0.33	0.89
SYRC_MOUSE	Arginine–tRNA ligase, cytoplasmic	228	75625	7.48	23	13	12	7	12	0.24	0.47
WDR61_MOUSE	WD repeat-containing protein 61	228	33752	5.1	6	4	3	2	3	0.18	0.28
IMA4_MOUSE	Importin subunit alpha-4	178	57737	4.8	10	7	6	4	4	0.32	0.34
RL7_MOUSE	60S ribosomal protein L7	227	31400	10.89	13	7	6	3	5	0.24	0.49
PSME2_MOUSE	Proteasome activator complex subunit 2	227	27040	5.54	14	7	5	4	5	0.34	0.84
SCRB2_MOUSE	Lysosome membrane protein 2	225	54009	4.99	13	8	6	4	6	0.2	0.36
AT2A2_MOUSE	Sarcoplasmic/endoplasmic reticulum calcium ATPase 2	224	114784	5.23	26	12	13	7	13	0.24	0.29
SODM_MOUSE	Superoxide dismutase [Mn], mitochondrial	222	24588	8.8	14	8	5	3	5	0.5	0.96
SYLC_MOUSE	Leucine–tRNA ligase, cytoplasmic	222	134106	6.64	22	7	13	4	13	0.19	0.13
PTGIS_MOUSE	Prostacyclin synthase	222	57011	6.26	28	17	13	9	6	0.52	0.93
PYGB_MOUSE	Glycogen phosphorylase, brain form	219	96668	6.28	16	10	9	6	9	0.16	0.3
MPCP_MOUSE	Phosphate carrier protein, mitochondrial	218	39606	9.36	15	11	8	5	8	0.32	0.88
LAMP1_MOUSE	Lysosome-associated membrane glycoprotein 1	217	43837	8.66	8	7	4	3	4	0.16	0.46
COPA_MOUSE	Coatomer subunit alpha	217	138344	7.69	23	11	16	9	16	0.23	0.31
KINH_MOUSE	Kinesin-1 heavy chain	216	109484	6.06	13	6	10	4	10	0.19	0.17
LG3BP_MOUSE	Galectin-3-binding protein	216	64450	5	12	5	8	5	8	0.29	0.38
PSB4_MOUSE	Proteasome subunit beta type-4	215	29097	5.47	5	3	3	2	3	0.23	0.33
IDI1_MOUSE	Isopentenyl-diphosphate delta-isomerase 1	215	26272	5.79	19	12	8	7	8	0.54	2.01
CNN2_MOUSE	Calponin-2	215	33134	7.53	17	9	9	4	9	0.59	1.12
NAA15_MOUSE	N-Alpha-acetyltransferase 15, NatA auxiliary subunit	212	100897	7.68	19	9	9	5	9	0.18	0.23
IF2P_MOUSE	Eukaryotic translation initiation factor 5B	212	137532	5.47	16	9	7	4	7	0.14	0.16
LGMN_MOUSE	Legumain	211	49341	5.92	10	9	3	3	3	0.17	0.4
DJC10_MOUSE	DnaJ homolog subfamily C member 10	210	90525	6.53	15	10	6	3	6	0.13	0.2
PFKAL_MOUSE	ATP-dependent 6-phosphofructokinase, liver type	210	85305	6.74	11	8	6	6	6	0.12	0.34
UGPA_MOUSE	UTP–glucose-1-phosphate uridylyltransferase	210	56944	7.18	8	6	6	5	6	0.26	0.44
RTN4_MOUSE	Reticulon-4	210	126535	4.47	11	9	6	4	6	0.12	0.14
CD34_MOUSE	Hematopoietic progenitor cell antigen CD34	208	40957	5.2	3	3	1	1	1	0.05	0.11
TPP2_MOUSE	Tripeptidyl-peptidase 2	208	139790	6.13	15	8	9	4	9	0.16	0.13
PCNA_MOUSE	Proliferating cell nuclear antigen	208	28766	4.66	15	8	8	6	8	0.63	1.37
TSPO_MOUSE	Translocator protein	205	18829	9.52	12	8	4	2	4	0.46	0.92
SNAA_MOUSE	Alpha-soluble NSF attachment protein	202	33168	5.3	13	7	7	4	7	0.43	0.65
PDXD1_MOUSE	Pyridoxal-dependent decarboxylase domain-containing protein 1	202	87281	5.31	6	4	4	3	4	0.1	0.15
PRDX1_MOUSE	Peroxiredoxin-1	202	22162	8.26	43	18	16	7	14	0.69	2.67
DHX9_MOUSE	ATP-dependent RNA helicase A	201	149381	6.39	19	12	11	6	11	0.16	0.18
MIC60_MOUSE	MICOS complex subunit Mic60	200	83848	6.18	14	8	4	3	4	0.1	0.16
AACS_MOUSE	Acetoacetyl-CoA synthetase	199	75152	6.25	24	9	11	7	11	0.36	0.56
SERC_MOUSE	Phosphoserine aminotransferase	199	40447	8.15	23	12	11	5	11	0.52	0.85
HYOU1_MOUSE	Hypoxia-upregulated protein 1	198	111112	5.12	14	7	9	4	9	0.18	0.21
INF2_MOUSE	Inverted formin-2	198	138474	5.09	18	8	10	4	10	0.14	0.13
PSMD6_MOUSE	26S proteasome non-ATPase regulatory subunit 6	197	45507	5.38	4	4	2	2	2	0.1	0.2
AL9A1_MOUSE	4-Trimethylaminobutyraldehyde dehydrogenase	196	53480	6.63	9	6	3	2	3	0.17	0.26
RN213_MOUSE	E3 ubiquitin-protein ligase RNF213	196	584411	6.35	23	8	21	7	21	0.07	0.05
CX7A2_MOUSE	Cytochrome c oxidase subunit 7A2, mitochondrial	196	9285	10.28	7	6	3	2	3	0.59	1.37
PSB5_MOUSE	Proteasome subunit beta type-5	196	28514	6.52	10	8	5	4	5	0.32	0.79
TMX3_MOUSE	Protein disulfide-isomerase TMX3	196	51815	5.02	4	4	2	2	2	0.11	0.17
DLDH_MOUSE	Dihydrolipoyl dehydrogenase, mitochondrial	195	54238	7.99	16	12	5	4	5	0.21	0.47
AEBP1_MOUSE	Adipocyte enhancer-binding protein 1	194	128284	5.02	4	3	2	1	2	0.02	0.03
MP2K1_MOUSE	Dual specificity mitogen-activated protein kinase 1	194	43446	6.24	9	5	6	2	5	0.32	0.21
MP2K2_MOUSE	Dual specificity mitogen-activated protein kinase 2	191	44374	6.58	5	5	2	2	1	0.12	0.21
STRAP_MOUSE	Serine-threonine kinase receptor-associated protein	193	38418	4.99	12	10	6	5	6	0.35	0.72
XPO1_MOUSE	Exportin-1	192	123013	5.72	18	10	12	8	12	0.2	0.31
EIF3I_MOUSE	Eukaryotic translation initiation factor 3 subunit I	191	36438	5.38	13	8	6	3	6	0.36	0.41
CPNE1_MOUSE	Copine-1	190	58849	5.4	11	7	7	4	7	0.3	0.33
EIF3K_MOUSE	Eukaryotic translation initiation factor 3 subunit K	189	25070	4.81	7	4	4	3	4	0.33	0.64
MIF_MOUSE	Macrophage migration inhibitory factor	189	12496	6.79	13	7	5	3	5	0.63	2.67
CYFP1_MOUSE	Cytoplasmic FMR1-interacting protein 1	189	145148	6.46	14	6	10	3	10	0.16	0.09
MYO1C_MOUSE	Unconventional myosin-Ic	188	121868	9.41	30	7	18	6	18	0.29	0.23
JAK1_MOUSE	Tyrosine-protein kinase JAK1	188	133282	7.63	11	5	6	1	6	0.1	0.03
ARPC2_MOUSE	Actin-related protein 2/3 complex subunit 2	188	34336	6.84	14	10	7	4	7	0.41	0.62
CSAD_MOUSE	Cysteine sulfinic acid decarboxylase	187	55109	6.17	5	3	3	1	3	0.11	0.08
PA2G4_MOUSE	Proliferation-associated protein 2G4	185	43671	6.41	4	4	3	3	3	0.15	0.33
DJB11_MOUSE	DnaJ homolog subfamily B member 11	185	40530	5.92	13	7	5	3	5	0.2	0.36
NSDHL_MOUSE	Sterol-4-alpha-carboxylate 3-dehydrogenase, decarboxylating	184	40660	7.71	20	8	7	6	7	0.39	0.85
P3H1_MOUSE	Prolyl 3-hydroxylase 1	184	83598	5.03	21	11	7	3	7	0.2	0.16
S10AA_MOUSE	Protein S100-A10	184	11179	6.27	5	5	2	2	2	0.44	1.06
DHB12_MOUSE	Very-long-chain 3-oxoacyl-CoA reductase	182	34719	9.55	15	10	7	5	7	0.45	0.82
SPRC_MOUSE	SPARC	182	34428	4.77	11	6	5	3	5	0.25	0.44
DHB7_MOUSE	3-Keto-steroid reductase	179	37293	6.25	5	5	4	4	4	0.21	0.56
MPRD_MOUSE	Cation-dependent mannose-6-phosphate receptor	179	31152	5.24	5	4	3	2	3	0.18	0.3
FKB10_MOUSE	Peptidyl-prolyl cis-trans isomerase FKBP10	179	64656	5.38	17	7	9	4	9	0.32	0.29
FRIL1_MOUSE	Ferritin light chain 1	178	20790	5.66	13	8	4	3	4	0.43	0.81
PGP_MOUSE	Glycerol-3-phosphate phosphatase	178	34519	5.21	3	3	1	1	1	0.06	0.13
IMPA1_MOUSE	Inositol monophosphatase 1	178	30416	5.08	7	6	3	2	3	0.12	0.31
SYCC_MOUSE	Cysteine–tRNA ligase, cytoplasmic	178	94800	6.32	12	5	7	5	7	0.18	0.25
FKBP4_MOUSE	Peptidyl-prolyl cis-trans isomerase FKBP4	177	51540	5.54	9	8	3	3	3	0.12	0.27
PPIB_MOUSE	Peptidyl-prolyl cis-trans isomerase B	177	23699	9.56	10	5	6	2	6	0.33	0.42
NASP_MOUSE	Nuclear autoantigenic sperm protein	177	83903	4.35	10	10	4	4	4	0.14	0.22
MPRI_MOUSE	Cation-independent mannose-6-phosphate receptor	177	273639	5.47	14	6	12	4	12	0.1	0.06
NQO1_MOUSE	NAD(P)H dehydrogenase [quinone] 1	175	30940	8.74	19	7	5	4	5	0.49	0.71
GCSH_MOUSE	Glycine cleavage system H protein, mitochondrial	173	18625	4.78	5	5	1	1	1	0.22	0.55
ITB1_MOUSE	Integrin beta-1	173	88173	5.68	22	10	12	6	12	0.29	0.33
CO1A2_MOUSE	Collagen alpha-2(I) chain	173	129478	9.27	20	12	10	4	10	0.12	0.14
HNRPM_MOUSE	Heterogeneous nuclear ribonucleoprotein M	172	77597	8.8	18	6	11	4	11	0.24	0.24
GHITM_MOUSE	Growth hormone-inducible transmembrane protein	172	37250	9.82	5	3	2	1	2	0.09	0.25
TTL12_MOUSE	Tubulin–tyrosine ligase-like protein 12	172	73996	5.39	15	7	8	5	8	0.27	0.33
RAB1B_MOUSE	Ras-related protein Rab-1B	118	22173	5.55	14	5	7	5	2	0.53	1.53
ACO13_MOUSE	Acyl-coenzyme A thioesterase 13	171	15173	8.95	8	7	3	3	3	0.41	1.24
MMP14_MOUSE	Matrix metalloproteinase-14	171	65877	8.07	6	5	4	3	4	0.13	0.21
PSA7_MOUSE	Proteasome subunit alpha type-7	171	27838	8.59	11	8	6	4	6	0.38	0.81
LEG3_MOUSE	Galectin-3	170	27498	8.46	24	11	7	5	7	0.35	1.12
PMVK_MOUSE	Phosphomevalonate kinase	169	21902	5.7	2	2	1	1	1	0.1	0.21
TPIS_MOUSE	Triosephosphate isomerase	169	32171	5.56	17	7	8	4	8	0.39	0.67
SEPT9_MOUSE	Septin-9	168	65534	9.01	11	4	7	3	7	0.22	0.21
UBR4_MOUSE	E3 ubiquitin-protein ligase UBR4	167	571927	5.72	24	10	16	6	16	0.07	0.05
GPDM_MOUSE	Glycerol-3-phosphate dehydrogenase, mitochondrial	167	80902	6.17	27	10	16	8	16	0.35	0.51
GLCM_MOUSE	Glucosylceramidase	166	57585	7.64	28	11	9	6	9	0.39	0.66
LA_MOUSE	Lupus La protein homolog	166	47727	9.77	9	7	7	5	7	0.23	0.55
VCAM1_MOUSE	Vascular cell adhesion protein 1	166	81265	5.21	7	6	3	3	3	0.07	0.17
AP2A1_MOUSE	AP-2 complex subunit alpha-1	165	107596	6.63	18	6	10	5	7	0.19	0.21
HMOX2_MOUSE	Heme oxygenase 2	164	35716	5.61	5	2	3	1	3	0.23	0.12
ITAV_MOUSE	Integrin alpha-V	164	115287	5.41	15	9	11	7	11	0.16	0.29
ECI1_MOUSE	Enoyl-CoA delta isomerase 1, mitochondrial	163	32230	9.12	4	4	2	2	2	0.15	0.29
SYEP_MOUSE	Bifunctional glutamate/proline–tRNA ligase	162	169972	7.75	33	10	18	7	18	0.22	0.19
KAD2_MOUSE	Adenylate kinase 2, mitochondrial	162	26452	6.96	5	5	2	2	2	0.16	0.37
NOMO1_MOUSE	Nodal modulator 1	161	133336	5.75	19	9	11	4	11	0.19	0.13
C1TM_MOUSE	Monofunctional C1-tetrahydrofolate synthase, mitochondrial	161	105662	6.58	9	7	4	3	4	0.09	0.13
AKAP2_MOUSE	A-kinase anchor protein 2	160	98519	5.13	4	3	3	2	3	0.05	0.09
SFPQ_MOUSE	Splicing factor, proline- and glutamine-rich	160	75394	9.45	5	3	2	1	2	0.05	0.06
BACH_MOUSE	Cytosolic acyl coenzyme A thioester hydrolase	159	42510	8.9	15	7	9	5	9	0.37	0.63
GLRX1_MOUSE	Glutaredoxin-1	159	11863	8.67	7	6	2	1	2	0.47	0.98
DHC24_MOUSE	Delta(24)-sterol reductase	159	60073	8.42	16	8	6	4	6	0.23	0.32
NMT1_MOUSE	Glycylpeptide N-tetradecanoyltransferase 1	159	56852	8.04	6	5	3	2	3	0.15	0.25
PRDX3_MOUSE	Thioredoxin-dependent peroxide reductase, mitochondrial	158	28109	7.15	7	6	4	3	4	0.35	0.56
CAVN1_MOUSE	Caveolae-associated protein 1	157	43927	5.43	8	5	4	2	4	0.18	0.21
PALLD_MOUSE	Palladin	156	152037	5.87	11	8	7	5	7	0.1	0.15
NPC1_MOUSE	Niemann-Pick C1 protein	156	142791	5.44	4	3	2	1	2	0.04	0.03
SRPRB_MOUSE	Signal recognition particle receptor subunit beta	156	29561	9.34	4	3	3	2	3	0.16	0.32
PCYOX_MOUSE	Prenylcysteine oxidase	156	56459	6.44	10	4	7	3	7	0.27	0.34
TRBM_MOUSE	Thrombomodulin	155	61827	4.5	9	8	2	2	2	0.13	0.22
PSB2_MOUSE	Proteasome subunit beta type-2	154	22892	6.52	25	11	6	6	6	0.54	1.95
AB1IP_MOUSE	Amyloid beta A4 precursor protein-binding family B member 1-interacting protein	154	74272	5.23	13	5	5	2	5	0.14	0.12
AGM1_MOUSE	Phosphoacetylglucosamine mutase	154	59415	5.8	8	6	3	2	3	0.13	0.23
APEH_MOUSE	Acylamino-acid-releasing enzyme	153	81529	5.36	7	6	5	4	5	0.16	0.23
CATL1_MOUSE	Cathepsin L1	153	37523	6.37	12	10	6	4	6	0.44	0.56
ATOX1_MOUSE	Copper transport protein ATOX1	151	7334	6.04	8	7	2	1	2	0.53	0.72
ALD2_MOUSE	Aldose reductase-related protein 2	151	36098	5.97	11	8	7	5	7	0.36	0.78
PPIC_MOUSE	Peptidyl-prolyl cis-trans isomerase C	151	22780	6.96	7	6	4	3	4	0.46	1.07
NRDC_MOUSE	Nardilysin	150	132808	4.77	14	6	8	3	8	0.15	0.1
MD2L1_MOUSE	Mitotic spindle assembly checkpoint protein MAD2A	150	23583	5.17	6	3	2	1	2	0.17	0.19
BROX_MOUSE	BRO1 domain-containing protein BROX	149	46172	7.59	6	4	4	2	4	0.22	0.2
SYTC_MOUSE	Threonine–tRNA ligase, cytoplasmic	148	83303	7.03	21	9	13	5	13	0.25	0.29
DNJA1_MOUSE	DnaJ homolog subfamily A member 1	148	44839	6.65	14	9	6	4	6	0.31	0.59
NNRE_MOUSE	NAD(P)H-hydrate epimerase	147	30953	7.59	11	9	4	3	4	0.3	0.49
CPNE3_MOUSE	Copine-3	147	59547	5.52	6	5	3	3	3	0.1	0.23
PTGR1_MOUSE	Prostaglandin reductase 1	147	35537	8.09	9	5	4	2	4	0.26	0.42
C1QBP_MOUSE	Complement component 1 Q subcomponent-binding protein, mitochondrial	146	30994	4.82	18	8	6	5	6	0.45	0.95
ERG7_MOUSE	Lanosterol synthase	145	83088	5.96	13	5	9	3	9	0.23	0.22
PAPS1_MOUSE	Bifunctional 3′-phosphoadenosine 5′-phosphosulfate synthase 1	144	70749	6.31	11	4	8	4	8	0.21	0.27
MTPN_MOUSE	Myotrophin	143	12853	5.27	11	7	6	3	6	0.62	1.59
VATA_MOUSE	V-type proton ATPase catalytic subunit A	143	68283	5.42	16	6	7	3	7	0.25	0.2
ATPG_MOUSE	ATP synthase subunit gamma, mitochondrial	143	32865	9.06	7	5	4	2	4	0.26	0.29
DDB1_MOUSE	DNA damage-binding protein 1	143	126772	5.14	20	10	11	5	11	0.21	0.18
RL4_MOUSE	60S ribosomal protein L4	143	47124	11.01	16	6	12	5	12	0.37	0.55
NDUB4_MOUSE	NADH dehydrogenase [ubiquinone] 1 beta subcomplex subunit 4	143	15072	9.89	4	4	1	1	1	0.16	0.31
CRTAP_MOUSE	Cartilage-associated protein	143	46140	5.46	12	7	5	4	5	0.2	0.43
TFR1_MOUSE	Transferrin receptor protein 1	143	85677	6.13	11	7	3	3	3	0.08	0.16
EI3JA_MOUSE	Eukaryotic translation initiation factor 3 subunit J-A	142	29326	4.69	5	3	2	2	2	0.1	0.33
ATLA3_MOUSE	Atlastin-3	142	60537	5.73	15	7	10	5	10	0.36	0.41
PUR4_MOUSE	Phosphoribosylformylglycinamidine synthase	142	144538	5.43	10	7	8	5	8	0.14	0.16
IPO9_MOUSE	Importin-9	142	115978	4.71	16	9	8	5	8	0.2	0.2
PSMD3_MOUSE	26S proteasome non-ATPase regulatory subunit 3	141	60680	8.48	10	8	6	5	6	0.14	0.41
AK1A1_MOUSE	Alcohol dehydrogenase [NADP(+)]	141	36564	6.9	5	3	4	2	4	0.27	0.26
DCTN3_MOUSE	Dynactin subunit 3	140	20965	5.84	4	3	3	2	3	0.31	0.8
SWP70_MOUSE	Switch-associated protein 70	138	68953	5.78	13	7	6	5	6	0.17	0.35
ATPO_MOUSE	ATP synthase subunit O, mitochondrial	137	23349	10	7	4	2	2	2	0.17	0.43
UN45A_MOUSE	Protein unc-45 homolog A	137	103382	6.01	9	3	7	2	7	0.13	0.08
SCOT1_MOUSE	Succinyl-CoA:3-ketoacid coenzyme A transferase 1, mitochondrial	137	55953	8.73	20	9	10	7	10	0.36	0.68
BCAT1_MOUSE	Branched-chain-amino-acid aminotransferase, cytosolic	137	42764	5.25	8	7	3	3	3	0.21	0.48
ASNS_MOUSE	Asparagine synthetase [glutamine-hydrolyzing]	137	64241	6.12	16	8	7	4	7	0.19	0.3
PPGB_MOUSE	Lysosomal protective protein	136	53809	5.56	7	4	3	2	3	0.1	0.17
PGBM_MOUSE	Basement membrane-specific heparan sulfate proteoglycan core protein	136	398039	5.88	25	6	13	2	13	0.07	0.02
CSN7A_MOUSE	COP9 signalosome complex subunit 7a	136	30206	7.68	14	7	6	3	6	0.51	0.51
HCD2_MOUSE	3-Hydroxyacyl-CoA dehydrogenase type-2	136	27402	8.53	6	3	4	2	4	0.2	0.35
BZW2_MOUSE	Basic leucine zipper and W2 domain-containing protein 2	135	48033	6.26	6	4	3	1	3	0.22	0.19
TOM70_MOUSE	Mitochondrial import receptor subunit TOM70	135	67547	7.46	10	7	6	4	4	0.24	0.28
XPP1_MOUSE	Xaa-Pro aminopeptidase 1	135	69547	5.33	14	7	7	4	4	0.18	0.27
NXP20_MOUSE	Protein Noxp20	135	60975	4.49	6	6	1	1	1	0.07	0.15
PAI1_MOUSE	Plasminogen activator inhibitor 1	134	45141	6.17	9	3	7	2	7	0.32	0.2
ROA0_MOUSE	Heterogeneous nuclear ribonucleoprotein A0	134	30512	9.35	9	3	4	2	4	0.24	0.31
GORS2_MOUSE	Golgi reassembly-stacking protein 2	134	47009	4.68	4	3	3	2	3	0.14	0.19
SMC2_MOUSE	Structural maintenance of chromosomes protein 2	131	134156	8.54	8	5	5	2	5	0.06	0.06
RAB21_MOUSE	Ras-related protein Rab-21	132	24091	8.11	7	5	5	4	5	0.21	0.99
SAC1_MOUSE	Phosphatidylinositide phosphatase SAC1	132	66901	6.85	8	3	5	3	5	0.16	0.21
HSPB1_MOUSE	Heat shock protein beta-1	131	23000	6.12	14	9	7	4	7	0.66	1.05
GLU2B_MOUSE	Glucosidase 2 subunit beta	131	58756	4.41	9	6	3	2	3	0.12	0.24
IMA1_MOUSE	Importin subunit alpha-1	130	57892	5.49	10	5	6	4	6	0.27	0.33
SEC13_MOUSE	Protein SEC13 homolog	130	35543	5.15	19	5	4	3	4	0.19	0.42
4F2_MOUSE	4F2 cell-surface antigen heavy chain	130	58300	5.62	5	5	2	2	2	0.1	0.15
CSN4_MOUSE	COP9 signalosome complex subunit 4	130	46256	5.57	9	4	6	2	6	0.25	0.2
ROAA_MOUSE	Heterogeneous nuclear ribonucleoprotein A/B	129	30812	7.68	11	9	6	6	6	0.2	1.24
ERF3A_MOUSE	Eukaryotic peptide chain release factor GTP-binding subunit ERF3A	129	68582	5.12	19	10	9	6	9	0.18	0.44
CUL4A_MOUSE	Cullin-4A	128	87697	8.53	4	2	3	2	1	0.07	0.1
CUL4B_MOUSE	Cullin-4B	102	110630	8.56	7	3	6	3	4	0.12	0.12
AHSA1_MOUSE	Activator of 90 kDa heat shock protein ATPase homolog 1	128	38093	5.41	6	3	4	2	4	0.23	0.24
PDLI1_MOUSE	PDZ and LIM domain protein 1	127	35752	6.38	9	5	5	4	5	0.25	0.59
ECHM_MOUSE	Enoyl-CoA hydratase, mitochondrial	127	31454	8.76	8	5	5	3	5	0.33	0.49
ITA5_MOUSE	Integrin alpha-5	126	114971	5.65	13	6	6	2	6	0.09	0.08
NDUBA_MOUSE	NADH dehydrogenase [ubiquinone] 1 beta subcomplex subunit 10	126	21010	8.19	6	4	4	3	4	0.31	0.8
RENBP_MOUSE	N-Acylglucosamine 2-epimerase	126	49739	5.69	8	5	5	3	5	0.21	0.29
NU155_MOUSE	Nuclear pore complex protein Nup155	125	155019	5.77	9	6	5	3	5	0.06	0.08
DDAH1_MOUSE	N(G),N(G)-Dimethylarginine dimethylaminohydrolase 1	125	31361	5.64	6	4	5	3	5	0.4	0.49
G6PE_MOUSE	GDH/6PGL endoplasmic bifunctional protein	124	88872	6.44	16	3	9	3	9	0.21	0.15
CO5A1_MOUSE	Collagen alpha-1(V) chain	124	183564	4.86	7	3	6	2	6	0.09	0.05
PLAP_MOUSE	Phospholipase A-2-activating protein	124	87166	5.77	6	2	5	1	5	0.15	0.05
PDLI5_MOUSE	PDZ and LIM domain protein 5	123	63259	8.61	15	6	7	3	7	0.21	0.22
NAGAB_MOUSE	Alpha-N-acetylgalactosaminidase	122	47204	6.02	7	5	6	4	6	0.23	0.42
PDIA5_MOUSE	Protein disulfide-isomerase A5	121	59229	7.25	5	5	4	4	4	0.19	0.33
PSMD5_MOUSE	26S proteasome non-ATPase regulatory subunit 5	121	55937	5.13	12	6	7	4	7	0.26	0.35
FRIH_MOUSE	Ferritin heavy chain	120	21053	5.53	9	6	5	3	5	0.48	0.8
OSTF1_MOUSE	Osteoclast-stimulating factor 1	119	23768	5.46	6	2	4	1	4	0.19	0.19
PFD5_MOUSE	Prefoldin subunit 5	119	17345	5.93	7	4	4	3	4	0.47	1.04
PCKGM_MOUSE	Phosphoenolpyruvate carboxykinase [GTP], mitochondrial	119	70482	6.92	16	7	10	7	10	0.26	0.51
SAE2_MOUSE	SUMO-activating enzyme subunit 2	119	70525	5.09	8	6	5	4	5	0.21	0.27
NSF_MOUSE	Vesicle-fusing ATPase	119	82561	6.52	7	3	7	3	7	0.17	0.16
THY1_MOUSE	Thy-1 membrane glycoprotein	118	18069	9.16	9	2	3	1	3	0.25	0.26
RRBP1_MOUSE	Ribosome-binding protein 1	117	172776	9.35	12	3	9	3	9	0.1	0.08
CYB5_MOUSE	Cytochrome b5	117	15232	4.96	9	4	3	1	3	0.43	1.94
ECI2_MOUSE	Enoyl-CoA delta isomerase 2, mitochondrial	117	43240	9.08	4	3	3	2	3	0.13	0.21
TCAF2_MOUSE	TRPM8 channel-associated factor 2	117	101532	6.07	5	5	3	3	3	0.06	0.13
RCN1_MOUSE	Reticulocalbin-1	117	38090	4.7	9	6	5	3	5	0.27	0.39
ELP1_MOUSE	Elongator complex protein 1	117	149489	5.67	9	2	6	1	6	0.09	0.03
NHLC2_MOUSE	NHL repeat-containing protein 2	117	78381	5.33	10	6	3	2	3	0.12	0.17
NQO2_MOUSE	Ribosyldihydronicotinamide dehydrogenase [quinone]	117	26231	6.54	6	4	4	3	4	0.32	0.61
C1TC_MOUSE	C-1-tetrahydrofolate synthase, cytoplasmic	116	101136	6.7	12	7	6	4	6	0.16	0.18
TWF1_MOUSE	Twinfilin-1	115	40054	6.21	7	3	7	3	6	0.36	0.37
HPCL1_MOUSE	Hippocalcin-like protein 1	115	22324	5.32	5	2	5	2	5	0.43	0.45
PEBP1_MOUSE	Phosphatidylethanolamine-binding protein 1	114	20817	5.19	16	6	5	3	5	0.57	0.81
FSTL1_MOUSE	Follistatin-related protein 1	114	34532	5.58	1	1	1	1	1	0.06	0.13
TIGAR_MOUSE	Fructose-2,6-bisphosphatase TIGAR	114	29172	8.45	3	2	2	1	2	0.16	0.15
HMOX1_MOUSE	Heme oxygenase 1	113	32908	6.08	14	6	4	3	4	0.3	0.46
FKB1A_MOUSE	Peptidyl-prolyl cis-trans isomerase FKBP1A	112	11915	7.88	10	5	4	3	4	0.66	1.79
GT251_MOUSE	Procollagen galactosyltransferase 1	112	71015	6.83	10	5	8	4	8	0.18	0.27
CD81_MOUSE	CD81 antigen	111	25797	5.54	5	4	2	2	2	0.18	0.38
IDH3A_MOUSE	Isocitrate dehydrogenase [NAD] subunit alpha, mitochondrial	111	39613	6.27	4	3	3	2	3	0.16	0.23
VMA5A_MOUSE	von Willebrand factor A domain-containing protein 5A	111	87087	6.15	6	5	4	3	4	0.09	0.15
LONM_MOUSE	Lon protease homolog, mitochondrial	111	105776	6.15	9	4	6	2	6	0.16	0.08
ECHB_MOUSE	Trifunctional enzyme subunit beta, mitochondrial	111	51353	9.43	10	2	6	2	6	0.23	0.18
SRPRA_MOUSE	Signal recognition particle receptor subunit alpha	110	69579	9.07	6	3	5	2	5	0.12	0.13
EIF3D_MOUSE	Eukaryotic translation initiation factor 3 subunit D	109	63948	5.79	8	4	5	2	5	0.2	0.14
CX6A1_MOUSE	Cytochrome c oxidase subunit 6A1, mitochondrial	109	12344	9.97	5	4	2	1	2	0.45	0.39
RISC_MOUSE	Retinoid-inducible serine carboxypeptidase	108	50932	5.48	16	8	10	6	10	0.3	0.77
PRDX2_MOUSE	Peroxiredoxin-2	108	21765	5.2	12	6	6	4	6	0.46	1.13
RL18_MOUSE	60S ribosomal protein L18	108	21631	11.79	2	2	2	2	2	0.14	0.46
PTGR3_MOUSE	Prostaglandin reductase-3	108	40503	7.01	6	5	3	3	3	0.23	0.51
PPID_MOUSE	Peptidyl-prolyl cis-trans isomerase D	108	40717	7.08	4	2	3	1	3	0.15	0.11
SGTA_MOUSE	Small glutamine-rich tetratricopeptide repeat-containing protein alpha	106	34301	4.99	7	6	2	2	2	0.05	0.27
HYEP_MOUSE	Epoxide hydrolase 1	106	52543	8.43	28	6	15	3	15	0.43	0.37
RAGP1_MOUSE	Ran GTPase-activating protein 1	105	63491	4.59	10	5	8	5	8	0.21	0.39
NCBP1_MOUSE	Nuclear cap-binding protein subunit 1	105	91868	6.17	9	3	8	2	8	0.15	0.1
FKBP9_MOUSE	Peptidyl-prolyl cis-trans isomerase FKBP9	104	62956	5.03	10	5	6	4	6	0.17	0.3
NB5R3_MOUSE	NADH-cytochrome b5 reductase 3	104	34106	8.55	13	5	7	5	7	0.55	0.84
CX6B1_MOUSE	Cytochrome c oxidase subunit 6B1	104	10065	8.96	4	3	2	2	2	0.29	1.23
COMT_MOUSE	Catechol O-methyltransferase	104	29467	5.52	10	7	4	3	4	0.24	0.53
BIN1_MOUSE	Myc box-dependent-interacting protein 1	103	64430	4.95	10	3	7	2	7	0.22	0.14
AN32B_MOUSE	Acidic leucine-rich nuclear phosphoprotein 32 family member B	102	31060	3.89	6	3	3	2	3	0.16	0.31
TIF1B_MOUSE	Transcription intermediary factor 1-beta	101	88791	5.52	6	4	3	3	3	0.06	0.15
SH3L2_MOUSE	SH3 domain-binding glutamic acid-rich-like protein 2	101	12247	5.48	3	3	2	2	2	0.26	0.95
ERO1A_MOUSE	ERO1-like protein alpha	101	54050	6.12	8	5	5	4	5	0.15	0.36
LMNA_MOUSE	Prelamin-A/C	101	74193	6.54	16	4	11	3	11	0.26	0.18
ODPB_MOUSE	Pyruvate dehydrogenase E1 component subunit beta, mitochondrial	101	38912	6.41	6	4	5	4	5	0.29	0.53
MPPA_MOUSE	Mitochondrial-processing peptidase subunit alpha	100	58242	6.36	2	1	1	1	1	0.03	0.07
EIF2A_MOUSE	Eukaryotic translation initiation factor 2A	100	64363	9.04	8	4	6	3	6	0.2	0.21
GSHR_MOUSE	Glutathione reductase, mitochondrial	100	53629	8.19	5	3	3	1	3	0.15	0.08
ADPRH_MOUSE	[Protein ADP-ribosylarginine] hydrolase	99	40042	5.46	8	5	3	3	3	0.22	0.37
DDX58_MOUSE	Probable ATP-dependent RNA helicase DDX58	99	105908	6.23	9	4	4	2	4	0.09	0.08
AP1M1_MOUSE	AP-1 complex subunit mu-1	99	48512	6.82	4	3	2	2	2	0.09	0.19
MVP_MOUSE	Major vault protein	98	95865	5.43	11	4	7	3	7	0.18	0.14
COPE_MOUSE	Coatomer subunit epsilon	98	34545	4.94	24	6	6	2	6	0.4	0.27
DPP2_MOUSE	Dipeptidyl peptidase 2	98	56218	5.17	3	2	2	1	2	0.07	0.08
PURA2_MOUSE	Adenylosuccinate synthetase isozyme 2	98	49990	5.98	12	3	7	3	7	0.25	0.28
PGS2_MOUSE	Decorin	98	39784	8.87	5	2	3	1	3	0.15	0.11
COPB2_MOUSE	Coatomer subunit beta′	98	102384	5.17	19	8	12	6	12	0.27	0.28
HCDH_MOUSE	Hydroxyacyl-coenzyme A dehydrogenase, mitochondrial	97	34442	8.76	5	3	4	2	4	0.23	0.27
CTBP1_MOUSE	C-terminal-binding protein 1	97	47715	6.28	6	3	3	1	3	0.12	0.09
BAG6_MOUSE	Large proline-rich protein BAG6	97	120962	5.46	6	2	4	2	4	0.08	0.07
MAP1B_MOUSE	Microtubule-associated protein 1B	97	270089	4.76	16	5	13	3	13	0.11	0.05
ATX10_MOUSE	Ataxin-10	97	53673	5.12	11	5	5	3	5	0.19	0.26
PRRC1_MOUSE	Protein PRRC1	97	46268	5.62	9	4	6	3	6	0.28	0.31
RRAS_MOUSE	Ras-related protein R-Ras	97	23749	6.32	4	3	3	2	3	0.22	0.42
CPT1A_MOUSE	Carnitine O-palmitoyltransferase 1, liver isoform	97	88195	8.83	4	2	2	2	2	0.09	0.1
SPRE_MOUSE	Sepiapterin reductase	97	27865	5.58	3	3	2	2	2	0.22	0.35
NEK7_MOUSE	Serine/threonine-protein kinase Nek7	96	34514	8.49	3	2	2	1	2	0.16	0.13
FACE1_MOUSE	CAAX prenyl protease 1 homolog	95	54699	6.49	6	2	4	1	4	0.17	0.08
STAM2_MOUSE	Signal transducing adapter molecule 2	95	57419	4.94	7	6	3	2	3	0.15	0.24
TMED5_MOUSE	Transmembrane emp24 domain-containing protein 5	94	26155	4.81	3	3	1	1	1	0.12	0.17
SYDC_MOUSE	Aspartate–tRNA ligase, cytoplasmic	94	57111	6.07	13	3	9	3	9	0.28	0.24
ACADM_MOUSE	Medium-chain specific acyl-CoA dehydrogenase, mitochondrial	94	46452	8.6	3	3	2	2	2	0.11	0.2
PDCL3_MOUSE	Phosducin-like protein 3	94	27564	4.66	7	5	4	3	4	0.36	0.57
SYWC_MOUSE	Tryptophan–tRNA ligase, cytoplasmic	94	54323	6.44	10	4	5	1	5	0.2	0.08
NIBL1_MOUSE	Niban-like protein 1	94	84765	5.65	14	8	7	5	7	0.15	0.34
CYB5B_MOUSE	Cytochrome b5 type B	94	16308	4.79	3	2	1	1	1	0.23	0.29
TNPO1_MOUSE	Transportin-1	93	102291	4.84	17	9	12	6	9	0.28	0.28
SCFD1_MOUSE	Sec1 family domain-containing protein 1	93	72277	5.98	8	3	5	3	5	0.19	0.19
PRDX5_MOUSE	Peroxiredoxin-5, mitochondrial	93	21884	9.1	13	3	7	3	7	0.48	0.76
PUR8_MOUSE	Adenylosuccinate lyase	92	54831	6.9	7	5	6	4	6	0.23	0.36
IAH1_MOUSE	Isoamyl acetate-hydrolyzing esterase 1 homolog	92	27956	5.34	3	2	1	1	1	0.07	0.16
VA0D1_MOUSE	V-type proton ATPase subunit d 1	91	40275	4.89	5	3	5	3	5	0.33	0.36
CMTD1_MOUSE	Catechol O-methyltransferase domain-containing protein 1	91	28943	8.61	2	2	1	1	1	0.1	0.15
AIFM1_MOUSE	Apoptosis-inducing factor 1, mitochondrial	90	66724	9.23	4	2	3	2	3	0.1	0.13
MSMO1_MOUSE	Methylsterol monooxygenase 1	90	34750	7.29	8	3	5	2	5	0.31	0.43
PON2_MOUSE	Serum paraoxonase/arylesterase 2	90	39592	5.49	2	1	2	1	2	0.1	0.11
LTOR3_MOUSE	Ragulator complex protein LAMTOR3	90	13544	6.73	4	3	3	2	3	0.4	0.83
ACACA_MOUSE	Acetyl-CoA carboxylase 1	90	265088	5.97	12	4	10	3	10	0.09	0.07
PGPI_MOUSE	Pyroglutamyl-peptidase 1	90	22919	5.21	4	4	2	2	2	0.21	0.43
CTND1_MOUSE	Catenin delta-1	89	104860	6.41	9	6	5	2	5	0.12	0.08
NDUA9_MOUSE	NADH dehydrogenase [ubiquinone] 1 alpha subcomplex subunit 9, mitochondrial	89	42498	9.75	6	4	4	2	4	0.16	0.22
PMM2_MOUSE	Phosphomannomutase 2	89	27639	6.01	2	2	1	1	1	0.11	0.16
FAAA_MOUSE	Fumarylacetoacetase	89	46146	6.7	4	4	3	3	3	0.16	0.31
TCP4_MOUSE	Activated RNA polymerase II transcriptional coactivator p15	89	14418	9.6	2	2	1	1	1	0.19	0.33
IDHP_MOUSE	Isocitrate dehydrogenase [NADP], mitochondrial	89	50874	8.88	12	4	7	3	6	0.27	0.39
DUS3_MOUSE	Dual specificity protein phosphatase 3	89	20459	6.07	2	1	2	1	2	0.19	0.22
LIMA1_MOUSE	LIM domain and actin-binding protein 1	88	84008	6.18	6	2	4	1	4	0.09	0.05
MESD_MOUSE	LRP chaperone MESD	88	25191	6.06	4	3	3	2	3	0.23	0.39
CYGB_MOUSE	Cytoglobin	88	21452	6.32	2	2	1	1	1	0.13	0.21
MA2A1_MOUSE	Alpha-mannosidase 2	88	131548	8.17	9	4	8	4	8	0.14	0.14
DPP3_MOUSE	Dipeptidyl peptidase 3	88	82846	5.26	11	8	7	5	7	0.22	0.29
NDUC2_MOUSE	NADH dehydrogenase [ubiquinone] 1 subunit C2	87	14154	9.24	2	2	1	1	1	0.16	0.33
CNPY4_MOUSE	Protein canopy homolog 4	86	28076	4.72	5	3	3	2	3	0.22	0.34
CATH_MOUSE	Pro-cathepsin H	86	37146	8.68	3	2	2	1	2	0.14	0.12
PSMD8_MOUSE	26S proteasome non-ATPase regulatory subunit 8	86	39905	9.61	9	3	6	2	6	0.4	0.23
RCC1_MOUSE	Regulator of chromosome condensation	86	44903	8.34	5	3	4	2	4	0.19	0.2
ORN_MOUSE	Oligoribonuclease, mitochondrial	86	26722	6.67	2	1	2	1	2	0.18	0.17
G6PC3_MOUSE	Glucose-6-phosphatase 3	86	38756	8.42	2	2	2	2	2	0.1	0.24
IMA5_MOUSE	Importin subunit alpha-5	86	60144	4.93	5	4	2	2	2	0.11	0.15
PSMD4_MOUSE	26S proteasome non-ATPase regulatory subunit 4	86	40678	4.67	7	4	4	2	4	0.17	0.23
GSTT3_MOUSE	Glutathione S-transferase theta-3	85	27385	7.63	3	3	1	1	1	0.14	0.16
PSA1_MOUSE	Proteasome subunit alpha type-1	85	29528	6	9	6	6	3	6	0.41	0.75
GPNMB_MOUSE	Transmembrane glycoprotein NMB	85	63635	7.55	6	4	3	3	3	0.06	0.22
FUBP2_MOUSE	Far upstream element-binding protein 2	84	76728	6.9	9	3	6	2	6	0.12	0.12
RS21_MOUSE	40S ribosomal protein S21	84	9136	8.71	6	3	1	1	1	0.17	0.56
ABRAL_MOUSE	Costars family protein ABRACL	84	9025	5.52	5	1	2	1	2	0.46	0.56
UMPS_MOUSE	Uridine 5′-monophosphate synthase	84	52259	6.17	7	3	3	2	3	0.15	0.17
PSA3_MOUSE	Proteasome subunit alpha type-3	84	28387	5.29	3	2	2	1	2	0.11	0.16
HGS_MOUSE	Hepatocyte growth factor-regulated tyrosine kinase substrate	83	85961	5.84	10	2	7	2	7	0.17	0.1
RIR1_MOUSE	Ribonucleoside-diphosphate reductase large subunit	83	90153	6.27	8	2	6	2	6	0.16	0.1
PGH2_MOUSE	Prostaglandin G/H synthase 2	83	68969	7	9	3	6	2	6	0.21	0.13
CSDE1_MOUSE	Cold shock domain-containing protein E1	83	88735	5.97	5	1	5	1	5	0.12	0.05
SAE1_MOUSE	SUMO-activating enzyme subunit 1	82	38596	5.24	9	6	3	3	3	0.17	0.38
NPS3B_MOUSE	Protein NipSnap homolog 3B	82	28290	9.51	3	3	1	1	1	0.11	0.16
TBCD_MOUSE	Tubulin-specific chaperone D	82	133236	6.08	5	4	4	3	4	0.08	0.1
COR1C_MOUSE	Coronin-1C	82	53087	6.65	7	3	5	3	5	0.17	0.27
SCMC1_MOUSE	Calcium-binding mitochondrial carrier protein SCaMC-1	82	52868	7.02	9	4	4	1	4	0.15	0.08
ECM29_MOUSE	Proteasome-associated protein ECM29 homolog	82	203573	6.68	21	7	16	6	16	0.18	0.13
LICH_MOUSE	Lysosomal acid lipase/cholesteryl ester hydrolase	81	45296	8.16	4	3	3	2	3	0.15	0.2
IMA7_MOUSE	Importin subunit alpha-7	81	59926	4.86	9	4	6	4	6	0.29	0.32
CLPT1_MOUSE	Cleft lip and palate transmembrane protein 1 homolog	81	75243	5.88	4	1	3	1	3	0.11	0.06
RWDD1_MOUSE	RWD domain-containing protein 1	81	27768	4.18	6	4	3	2	3	0.3	0.35
DDX21_MOUSE	Nucleolar RNA helicase 2	80	93493	9.19	7	5	3	2	3	0.08	0.09
XPO5_MOUSE	Exportin-5	80	136883	5.57	16	8	7	3	7	0.11	0.13
SDCB1_MOUSE	Syntenin-1	80	32359	6.66	9	5	4	2	4	0.25	0.29
GLOD4_MOUSE	Glyoxalase domain-containing protein 4	80	33296	5.28	8	5	3	2	3	0.19	0.28
PLD3_MOUSE	Phospholipase D3	80	54354	6.07	10	4	7	3	7	0.34	0.36
LARP1_MOUSE	La-related protein 1	80	121050	8.87	6	3	4	1	4	0.05	0.04
NDUAA_MOUSE	NADH dehydrogenase [ubiquinone] 1 alpha subcomplex subunit 10, mitochondrial	80	40578	7.63	2	2	2	2	2	0.09	0.23
EIF3C_MOUSE	Eukaryotic translation initiation factor 3 subunit C	80	105465	5.55	14	4	10	3	10	0.18	0.13
GNPI1_MOUSE	Glucosamine-6-phosphate isomerase 1	79	32528	6.13	5	3	3	3	3	0.21	0.47
ADDA_MOUSE	Alpha-adducin	79	80596	5.62	6	2	4	1	4	0.12	0.05
SEPT5_MOUSE	Septin-5	79	42721	6.21	16	5	9	4	9	0.42	0.63
TADBP_MOUSE	TAR DNA-binding protein 43	79	44519	6.26	3	3	1	1	1	0.07	0.1
MAP4_MOUSE	Microtubule-associated protein 4	79	117357	4.9	7	2	5	1	5	0.09	0.04
RAB9A_MOUSE	Ras-related protein Rab-9A	79	22895	5.43	4	4	2	2	2	0.19	0.43
AGAL_MOUSE	Alpha-galactosidase A	79	47611	5.44	6	4	5	3	5	0.21	0.3
ULA1_MOUSE	NEDD8-activating enzyme E1 regulatory subunit	78	60236	5.34	4	3	3	2	3	0.15	0.15
EI2BA_MOUSE	Translation initiation factor eIF-2B subunit alpha	78	33795	8.48	4	2	3	2	3	0.17	0.28
LPPRC_MOUSE	Leucine-rich PPR motif-containing protein, mitochondrial	78	156516	6.42	10	6	7	4	7	0.12	0.11
SYK_MOUSE	Lysine–tRNA ligase	78	67796	5.65	10	5	6	4	6	0.17	0.28
SWI5_MOUSE	DNA repair protein SWI5 homolog	78	10254	4.67	1	1	1	1	1	0.19	0.49
FAM3C_MOUSE	Protein FAM3C	77	24737	8.52	5	2	2	1	2	0.13	0.18
BLMH_MOUSE	Bleomycin hydrolase	77	52477	6.04	8	3	6	2	6	0.26	0.17
TOIP1_MOUSE	Torsin-1A-interacting protein 1	77	66740	6.58	2	2	2	2	2	0.04	0.13
NUDC_MOUSE	Nuclear migration protein nudC	77	38334	5.17	2	1	2	1	2	0.06	0.11
GSHB_MOUSE	Glutathione synthetase	77	52214	5.56	4	3	3	2	3	0.14	0.17
SPTC2_MOUSE	Serine palmitoyltransferase 2	77	62941	8.43	8	4	4	1	4	0.14	0.07
CP20A_MOUSE	Cytochrome P450 20A1	77	52116	6.48	4	2	3	1	3	0.13	0.08
USP9X_MOUSE	Probable ubiquitin carboxyl-terminal hydrolase FAF-X	76	290526	5.57	19	6	10	3	10	0.08	0.04
ASPH_MOUSE	Aspartyl/asparaginyl beta-hydroxylase	75	82991	4.97	9	4	8	4	8	0.17	0.22
ALDR_MOUSE	Aldose reductase	76	35709	6.71	4	2	3	1	3	0.19	0.12
MGAT1_MOUSE	Alpha-1,3-mannosyl-glycoprotein 2-beta-N-acetylglucosaminyltransferase	76	51658	9.02	6	5	2	2	2	0.12	0.18
ATP5H_MOUSE	ATP synthase subunit d, mitochondrial	76	18738	5.52	5	3	4	3	4	0.42	0.93
AT5F1_MOUSE	ATP synthase F(0) complex subunit B1, mitochondrial	76	28930	9.11	5	1	5	1	5	0.34	0.15
IF2B_MOUSE	Eukaryotic translation initiation factor 2 subunit 2	76	38068	5.61	6	3	4	2	4	0.19	0.24
HEAT3_MOUSE	HEAT repeat-containing protein 3	75	74258	4.91	5	3	3	2	3	0.08	0.12
LCLT1_MOUSE	Lysocardiolipin acyltransferase 1	75	44371	8.77	2	2	2	2	2	0.14	0.21
MCM6_MOUSE	DNA replication licensing factor MCM6	75	92809	5.32	4	3	4	3	4	0.09	0.14
NDUA8_MOUSE	NADH dehydrogenase [ubiquinone] 1 alpha subcomplex subunit 8	75	19979	8.76	3	2	2	1	2	0.23	0.23
POSTN_MOUSE	Periostin	75	93085	7.27	11	4	6	2	6	0.18	0.09
ACDSB_MOUSE	Short/branched chain specific acyl-CoA dehydrogenase, mitochondrial	74	47843	8	4	1	3	1	3	0.13	0.09
EIF1_MOUSE	Eukaryotic translation initiation factor 1	74	12739	6.89	2	1	2	1	2	0.33	0.38
IF1A_MOUSE	Eukaryotic translation initiation factor 1A	73	16492	5.07	4	1	4	1	1	0.26	0.28
CHMP5_MOUSE	Charged multivesicular body protein 5	73	24560	4.65	2	2	1	1	1	0.12	0.18
S10A4_MOUSE	Protein S100-A4	73	11714	5.23	6	5	3	3	3	0.25	1.82
ITM2C_MOUSE	Integral membrane protein 2C	73	30463	8.83	2	2	1	1	1	0.08	0.15
MEP50_MOUSE	Methylosome protein 50	73	36919	5.09	2	2	1	1	1	0.09	0.12
NDUS8_MOUSE	NADH dehydrogenase [ubiquinone] iron-sulfur protein 8, mitochondrial	73	24023	5.89	3	3	2	2	2	0.09	0.41
ZFPL1_MOUSE	Zinc finger protein-like 1	73	34133	8.57	7	2	3	2	3	0.2	0.27
USMG5_MOUSE	Upregulated during skeletal muscle growth protein 5	72	6377	9.84	2	2	1	1	1	0.26	0.87
VATL_MOUSE	V-type proton ATPase 16 kDa proteolipid subunit	72	15798	9.1	1	1	1	1	1	0.2	0.3
SFRP1_MOUSE	Secreted frizzled-related protein 1	72	35389	9.17	3	2	2	1	2	0.12	0.12
AAAS_MOUSE	Aladin	72	59393	6.39	3	3	1	1	1	0.03	0.07
ABD12_MOUSE	Monoacylglycerol lipase ABHD12	72	45241	8.9	3	1	2	1	2	0.07	0.1
SYIM_MOUSE	Isoleucine–tRNA ligase, mitochondrial	72	112732	6.37	7	5	5	3	5	0.11	0.12
MAPK2_MOUSE	MAP kinase-activated protein kinase 2	71	44022	8.77	7	2	5	2	5	0.19	0.21
BIEA_MOUSE	Biliverdin reductase A	71	33504	6.53	6	5	4	4	4	0.25	0.64
AP3D1_MOUSE	AP-3 complex subunit delta-1	71	134996	7.04	4	3	3	2	3	0.04	0.06
ALG5_MOUSE	Dolichyl-phosphate beta-glucosyltransferase	71	36767	8.83	4	2	2	2	2	0.12	0.25
CD99_MOUSE	CD99 antigen	71	16772	5.14	2	2	1	1	1	0.27	0.28
SYQ_MOUSE	Glutamine–tRNA ligase	70	87621	6.93	8	2	5	2	5	0.12	0.1
TCEA1_MOUSE	Transcription elongation factor A protein 1	70	33859	8.64	5	5	1	1	1	0.09	0.13
XDH_MOUSE	Xanthine dehydrogenase/oxidase	70	146468	7.62	14	6	6	3	6	0.1	0.12
ANXA7_MOUSE	Annexin A7	70	49893	5.91	5	2	5	2	5	0.16	0.18
IPYR2_MOUSE	Inorganic pyrophosphatase 2, mitochondrial	70	38090	6.51	8	3	5	2	5	0.16	0.24
CY1_MOUSE	Cytochrome c1, heme protein, mitochondrial	70	35305	9.24	2	1	2	1	2	0.12	0.12
NP1L4_MOUSE	Nucleosome assembly protein 1-like 4	70	42653	4.56	6	3	5	3	4	0.24	0.34
NU133_MOUSE	Nuclear pore complex protein Nup133	70	128539	5.06	5	2	5	2	5	0.09	0.07
SIAS_MOUSE	Sialic acid synthase	70	39998	6.61	2	1	1	1	1	0.1	0.11
ETFA_MOUSE	Electron transfer flavoprotein subunit alpha, mitochondrial	69	34988	8.62	10	5	5	3	5	0.33	0.43
GALM_MOUSE	Aldose 1-epimerase	69	37775	6.26	3	1	2	1	2	0.12	0.12
ABCF1_MOUSE	ATP-binding cassette sub-family F member 1	69	94887	6.15	3	2	2	1	2	0.06	0.05
T176B_MOUSE	Transmembrane protein 176B	68	28349	8.03	2	1	2	1	2	0.22	0.16
ICAL_MOUSE	Calpastatin	68	84871	5.37	6	3	4	2	4	0.1	0.1
THIM_MOUSE	3-Ketoacyl-CoA thiolase, mitochondrial	68	41803	8.33	3	1	3	1	3	0.13	0.1
CAPR1_MOUSE	Caprin-1	67	78121	5.14	7	2	5	2	5	0.11	0.11
PTPA_MOUSE	Serine/threonine-protein phosphatase 2A activator	67	36687	5.95	2	2	2	2	2	0.11	0.25
ATP5L_MOUSE	ATP synthase subunit g, mitochondrial	67	11417	9.74	3	2	2	1	2	0.32	0.43
UBA6_MOUSE	Ubiquitin-like modifier-activating enzyme 6	67	117891	5.75	7	1	6	1	6	0.11	0.04
VASP_MOUSE	Vasodilator-stimulated phosphoprotein	67	39642	8.69	5	2	3	2	3	0.08	0.23
E41L2_MOUSE	Band 4.1-like protein 2	67	109873	5.31	8	4	5	3	5	0.07	0.12
GLRX3_MOUSE	Glutaredoxin-3	67	37754	5.42	16	7	8	6	8	0.46	0.94
TRXR1_MOUSE	Thioredoxin reductase 1, cytoplasmic	66	67042	7.42	12	4	6	2	6	0.19	0.13
IASPP_MOUSE	RelA-associated inhibitor	66	88921	6.39	3	1	3	1	3	0.1	0.05
NUP85_MOUSE	Nuclear pore complex protein Nup85	66	74728	5.37	6	3	4	1	4	0.09	0.06
DDX18_MOUSE	ATP-dependent RNA helicase DDX18	66	74134	9.54	6	4	3	2	3	0.07	0.12
NEUL_MOUSE	Neurolysin, mitochondrial	66	80378	6.01	10	4	7	2	7	0.18	0.11
MLEC_MOUSE	Malectin	66	32322	5.73	8	3	6	2	6	0.37	0.47
GPC4_MOUSE	Glypican-4	65	62546	5.96	3	1	3	1	3	0.13	0.07
KIME_MOUSE	Mevalonate kinase	65	41851	6.22	4	3	2	1	2	0.14	0.1
EIF3L_MOUSE	Eukaryotic translation initiation factor 3 subunit L	65	66570	6.01	5	3	4	2	4	0.13	0.13
G3BP1_MOUSE	Ras GTPase-activating protein-binding protein 1	65	51797	5.41	10	3	9	3	9	0.31	0.27
CTNB1_MOUSE	Catenin beta-1	64	85416	5.53	10	3	5	2	5	0.13	0.1
ACADL_MOUSE	Long-chain specific acyl-CoA dehydrogenase, mitochondrial	64	47877	8.53	10	3	5	3	3	0.17	0.3
HXK1_MOUSE	Hexokinase-1	64	108234	6.37	7	3	6	3	6	0.09	0.12
TF_MOUSE	Tissue factor	64	32914	9.4	2	2	1	1	1	0.09	0.13
MYEF2_MOUSE	Myelin expression factor 2	64	63254	8.96	3	1	3	1	3	0.05	0.07
ANM5_MOUSE	Protein arginine N-methyltransferase 5	64	72634	5.99	16	3	7	1	7	0.25	0.12
MED11_MOUSE	Mediator of RNA polymerase II transcription subunit 11	63	13123	5.71	3	3	1	1	1	0.21	0.86
HEAT6_MOUSE	HEAT repeat-containing protein 6	63	128840	6.88	5	3	3	1	3	0.06	0.03
NAMPT_MOUSE	Nicotinamide phosphoribosyltransferase	63	55413	6.69	3	2	2	2	2	0.09	0.16
MYO9B_MOUSE	Unconventional myosin-Ixb	63	238685	8.82	3	1	3	1	3	0.03	0.02
AL1L2_MOUSE	Mitochondrial 10-formyltetrahydrofolate dehydrogenase	63	101526	5.93	5	1	4	1	4	0.08	0.04
HSBP1_MOUSE	Heat shock factor-binding protein 1	63	8605	4.11	2	1	1	1	1	0.25	0.59
MYG1_MOUSE	UPF0160 protein MYG1, mitochondrial	63	42696	6.54	1	1	1	1	1	0.04	0.1
RBM3_MOUSE	RNA-binding protein 3	62	16595	6.84	11	4	4	2	4	0.46	0.64
SLIT3_MOUSE	Slit homolog 3 protein	62	167617	7.95	13	2	7	2	7	0.11	0.05
PPM1B_MOUSE	Protein phosphatase 1B	62	42768	5.04	3	1	2	1	2	0.11	0.1
DPEP1_MOUSE	Dipeptidase 1	62	45693	5.9	4	1	3	1	3	0.23	0.1
F10A1_MOUSE	Hsc70-interacting protein	62	41630	5.19	6	3	3	2	3	0.12	0.22
MCFD2_MOUSE	Multiple coagulation factor deficiency protein 2 homolog	62	16158	4.55	1	1	1	1	1	0.17	0.29
RM12_MOUSE	39S ribosomal protein L12, mitochondrial	62	21695	9.34	8	2	3	1	3	0.2	0.46
FDFT_MOUSE	Squalene synthase	61	48123	5.91	5	2	2	2	2	0.06	0.19
LGUL_MOUSE	Lactoylglutathione lyase	61	20796	5.24	8	3	4	1	4	0.33	0.22
CDK4_MOUSE	Cyclin-dependent kinase 4	54	33729	6.16	6	2	5	2	5	0.31	0.28
CDC37_MOUSE	Hsp90 co-chaperone Cdc37	61	44565	5.24	4	2	3	2	2	0.13	0.21
CDK1_MOUSE	Cyclin-dependent kinase 1	61	34085	8.39	10	6	5	3	5	0.27	0.44
LETM1_MOUSE	Mitochondrial proton/calcium exchanger protein	61	82937	6.16	7	1	6	1	6	0.15	0.05
PGM1_MOUSE	Phosphoglucomutase-1	60	61380	6.14	6	3	6	3	6	0.29	0.23
LAMP2_MOUSE	Lysosome-associated membrane glycoprotein 2	60	45652	7.05	12	4	8	3	8	0.25	0.31
PKD2_MOUSE	Polycystin-2	60	108914	5.47	4	1	4	1	4	0.08	0.04
UCHL1_MOUSE	Ubiquitin carboxyl-terminal hydrolase isozyme L1	60	24822	5.14	7	4	5	3	5	0.38	0.65
COPD_MOUSE	Coatomer subunit delta	60	57193	5.89	18	4	12	3	12	0.34	0.24
IRGM1_MOUSE	Immunity-related GTPase family M protein 1	60	46522	8.56	4	3	2	1	2	0.13	0.09
AKA12_MOUSE	A-kinase anchor protein 12	60	180586	4.39	11	2	5	2	5	0.05	0.05
MP2K4_MOUSE	Dual specificity mitogen-activated protein kinase 4	60	44085	8.28	5	2	5	2	5	0.27	0.21
NCLN_MOUSE	Nicalin	59	62868	6.03	3	2	2	2	2	0.06	0.14
LMF2_MOUSE	Lipase maturation factor 2	58	79947	9.99	4	3	2	2	2	0.06	0.11
FHL1_MOUSE	Four and a half LIM domains protein 1	59	31867	8.76	6	3	3	2	3	0.17	0.3
ARHG7_MOUSE	Rho guanine nucleotide exchange factor 7	59	96995	6.35	2	1	2	1	2	0.04	0.04
PP14B_MOUSE	Protein phosphatase 1 regulatory subunit 14B	59	15947	4.74	10	6	2	2	2	0.29	0.67
MANF_MOUSE	Mesencephalic astrocyte-derived neurotrophic factor	59	20361	8.34	12	4	3	3	3	0.21	0.84
ACOT2_MOUSE	Acyl-coenzyme A thioesterase 2, mitochondrial	59	49626	6.88	4	3	2	2	2	0.08	0.18
S23IP_MOUSE	SEC23-interacting protein	59	110711	5.63	11	6	6	3	6	0.14	0.12
ERP29_MOUSE	Endoplasmic reticulum resident protein 29	58	28805	5.9	7	3	2	2	2	0.14	0.33
CO6A1_MOUSE	Collagen alpha-1(VI) chain	58	108422	5.2	5	2	4	1	4	0.1	0.04
DBNL_MOUSE	Drebrin-like protein	58	48670	4.9	4	2	2	1	2	0.1	0.09
SCPDL_MOUSE	Saccharopine dehydrogenase-like oxidoreductase	58	47099	8.86	6	3	3	1	3	0.2	0.09
PP1R7_MOUSE	Protein phosphatase 1 regulatory subunit 7	58	41266	4.85	5	3	2	1	2	0.14	0.22
MGST1_MOUSE	Microsomal glutathione S-transferase 1	58	17540	9.67	2	1	1	1	1	0.21	0.26
MON2_MOUSE	Protein MON2 homolog	58	188958	5.77	6	2	5	2	5	0.08	0.05
PARK7_MOUSE	Protein/nucleic acid deglycase DJ-1	57	20008	6.32	3	2	3	2	3	0.27	0.51
WASC4_MOUSE	WASH complex subunit 4	57	136283	6.99	5	2	5	2	5	0.09	0.06
AKAP1_MOUSE	A-kinase anchor protein 1, mitochondrial	57	92137	4.91	2	1	2	1	2	0.03	0.05
MALD1_MOUSE	MARVEL domain-containing protein 1	57	19074	9.77	5	3	2	1	2	0.21	0.24
SPEE_MOUSE	Spermidine synthase	57	33973	5.31	7	3	5	3	5	0.3	0.44
RFIP5_MOUSE	Rab11 family-interacting protein 5	57	69510	9.17	5	3	2	1	2	0.04	0.06
EXOC1_MOUSE	Exocyst complex component 1	57	101825	6.09	6	3	5	2	5	0.13	0.09
DOCK7_MOUSE	Dedicator of cytokinesis protein 7	56	241286	6.25	15	2	8	2	8	0.08	0.04
NDUA4_MOUSE	Cytochrome c oxidase subunit NDUFA4	56	9321	9.52	4	2	2	1	2	0.46	0.54
TGFI1_MOUSE	Transforming growth factor beta-1-induced transcript 1 protein	56	50068	6.28	8	4	3	2	3	0.18	0.18
HUWE1_MOUSE	E3 ubiquitin-protein ligase HUWE1	56	482332	5.1	10	2	9	1	9	0.04	0.01
LBR_MOUSE	Lamin-B receptor	56	71395	9.43	4	1	4	1	4	0.09	0.06
MARC2_MOUSE	Mitochondrial amidoxime reducing component 2	56	38170	8.95	4	2	3	2	3	0.18	0.24
CMC1_MOUSE	Calcium-binding mitochondrial carrier protein Aralar1	56	74523	8.43	4	3	2	2	2	0.05	0.12
BAX_MOUSE	Apoptosis regulator BAX	56	21381	4.86	1	1	1	1	1	0.07	0.21
BASP1_MOUSE	Brain acid soluble protein 1	56	22074	4.5	14	2	1	1	1	0.14	0.21
HINT1_MOUSE	Histidine triad nucleotide-binding protein 1	56	13768	6.36	4	1	4	1	4	0.6	0.35
SCYL1_MOUSE	N-terminal kinase-like protein	56	89104	6.03	2	1	2	1	2	0.06	0.05
QCR2_MOUSE	Cytochrome b-c1 complex subunit 2, mitochondrial	55	48205	9.26	2	1	2	1	2	0.08	0.09
TMF1_MOUSE	TATA element modulatory factor	55	121729	4.83	4	1	4	1	4	0.07	0.03
RENT1_MOUSE	Regulator of nonsense transcripts 1	55	123889	6.18	7	2	6	2	6	0.13	0.07
IKIP_MOUSE	Inhibitor of nuclear factor kappa-B kinase-interacting protein	55	42505	5.03	6	4	4	3	4	0.19	0.34
HPRT_MOUSE	Hypoxanthine-guanine phosphoribosyltransferase	53	24555	6.21	6	4	3	3	2	0.32	0.66
ISC2A_MOUSE	Isochorismatase domain-containing protein 2A	55	22403	8.25	1	1	1	1	1	0.13	0.2
NU4M_MOUSE	NADH-ubiquinone oxidoreductase chain 4	55	51847	9.42	3	1	2	1	2	0.04	0.08
CTL2_MOUSE	Choline transporter-like protein 2	55	80057	9.06	5	4	1	1	1	0.02	0.05
JIP4_MOUSE	C-Jun-amino-terminal kinase-interacting protein 4	54	146129	5.05	8	2	6	2	6	0.09	0.06
5NT3B_MOUSE	7-Methylguanosine phosphate-specific 5′-nucleotidase	54	34403	5.82	1	1	1	1	1	0.07	0.13
LAP2A_MOUSE	Lamina-associated polypeptide 2, isoforms alpha/zeta	54	75122	8.29	4	1	2	1	2	0.05	0.06
EM55_MOUSE	55 kDa erythrocyte membrane protein	54	52194	6.72	6	3	4	3	4	0.16	0.27
KAP0_MOUSE	cAMP-dependent protein kinase type I-alpha regulatory subunit	54	43158	5.27	5	2	3	1	3	0.15	0.1
GDIR2_MOUSE	Rho GDP-dissociation inhibitor 2	54	22836	4.97	7	4	4	3	4	0.26	0.72
NDUS2_MOUSE	NADH dehydrogenase [ubiquinone] iron-sulfur protein 2, mitochondrial	54	52592	6.52	4	1	3	1	3	0.14	0.08
SYHC_MOUSE	Histidine–tRNA ligase, cytoplasmic	53	57396	5.79	4	3	4	3	4	0.17	0.24
IVD_MOUSE	Isovaleryl-CoA dehydrogenase, mitochondrial	54	46296	8.53	10	2	4	2	4	0.26	0.2
DHRS1_MOUSE	Dehydrogenase/reductase SDR family member 1	54	33983	8.66	1	1	1	1	1	0.06	0.13
SRP68_MOUSE	Signal recognition particle subunit SRP68	53	70530	8.77	5	1	5	1	5	0.13	0.06
PREP_MOUSE	Presequence protease, mitochondrial	53	117297	6.76	6	4	6	4	6	0.13	0.15
GUAA_MOUSE	GMP synthase [glutamine-hydrolyzing]	53	76675	6.29	3	2	2	1	2	0.09	0.06
NT5C_MOUSE	5′(3′)-Deoxyribonucleotidase, cytosolic type	53	23062	5.31	2	1	2	1	2	0.2	0.2
DNMT1_MOUSE	DNA (cytosine-5)-methyltransferase 1	53	183074	7.92	6	1	6	1	6	0.08	0.02
GALK1_MOUSE	Galactokinase	53	42268	5.17	2	1	2	1	2	0.07	0.1
PSD12_MOUSE	26S proteasome non-ATPase regulatory subunit 12	53	52861	6.66	11	3	8	3	8	0.25	0.27
GLMP_MOUSE	Glycosylated lysosomal membrane protein	52	43776	5.73	4	1	3	1	3	0.18	0.1
UD17C_MOUSE	UDP-glucuronosyltransferase 1-7C	52	59719	8.64	6	3	5	3	5	0.18	0.23
DHPR_MOUSE	Dihydropteridine reductase	52	25554	7.67	3	1	2	1	2	0.19	0.18
NAA25_MOUSE	N-Alpha-acetyltransferase 25, NatB auxiliary subunit	52	111637	6.1	2	1	2	1	2	0.04	0.04
LDAH_MOUSE	Lipid droplet-associated hydrolase	52	37349	8.5	4	2	3	1	3	0.22	0.12
TR10B_MOUSE	Tumor necrosis factor receptor superfamily member 10B	52	42138	6.75	1	1	1	1	1	0.06	0.1
BAG2_MOUSE	BAG family molecular chaperone regulator 2	52	23459	6.01	7	1	5	1	5	0.38	0.19
DNPEP_MOUSE	Aspartyl aminopeptidase	52	52174	6.82	10	4	3	1	3	0.19	0.17
ODO2_MOUSE	Dihydrolipoyllysine-residue succinyltransferase component of 2-oxoglutarate dehydrogenase complex, mitochondrial	52	48963	9.11	4	2	2	1	2	0.07	0.09
TBCB_MOUSE	Tubulin-folding cofactor B	52	27368	5.14	2	2	1	1	1	0.08	0.16
PNKP_MOUSE	Bifunctional polynucleotide phosphatase/kinase	51	57188	8.03	1	1	1	1	1	0.04	0.08
ASAH1_MOUSE	Acid ceramidase	51	44641	8.68	10	4	6	3	6	0.24	0.32
VATE1_MOUSE	V-type proton ATPase subunit E 1	51	26141	8.44	4	2	3	1	3	0.3	0.17
ITPA_MOUSE	Inosine triphosphate pyrophosphatase	51	21883	5.6	4	1	2	1	2	0.15	0.21
MCM2_MOUSE	DNA replication licensing factor MCM2	51	102013	5.49	6	2	4	1	4	0.09	0.04
B2MG_MOUSE	Beta-2-microglobulin	50	13770	8.55	2	1	2	1	2	0.31	0.35
MRCKB_MOUSE	Serine/threonine-protein kinase MRCK beta	50	194630	6.05	8	5	5	2	5	0.06	0.04
TCOF_MOUSE	Treacle protein	50	134921	9.35	3	2	2	1	2	0.04	0.03
ACOT9_MOUSE	Acyl-coenzyme A thioesterase 9, mitochondrial	50	50528	8.74	8	3	5	2	5	0.23	0.18
RRP1_MOUSE	Ribosomal RNA processing protein 1 homolog A	50	54743	5.04	1	1	1	1	1	0.05	0.08
CHRD1_MOUSE	Cysteine and histidine-rich domain-containing protein 1	50	37327	8.13	4	1	3	1	3	0.18	0.12
HMGCL_MOUSE	Hydroxymethylglutaryl-CoA lyase, mitochondrial	50	34217	8.7	5	2	4	2	4	0.18	0.27
ANFY1_MOUSE	Rabankyrin-5	49	128571	5.58	6	3	6	3	6	0.11	0.1
MOGS_MOUSE	Mannosyl-oligosaccharide glucosidase	49	91774	9.06	7	4	4	3	4	0.1	0.15
SAMH1_MOUSE	Deoxynucleoside triphosphate triphosphohydrolase SAMHD1	49	72604	8.17	7	3	6	3	6	0.18	0.19
MPRIP_MOUSE	Myosin phosphatase Rho-interacting protein	49	116337	5.9	6	1	4	1	4	0.07	0.04
G6PD1_MOUSE	Glucose-6-phosphate 1-dehydrogenase X	49	59225	6.06	17	4	9	2	9	0.32	0.15
ATP5I_MOUSE	ATP synthase subunit e, mitochondrial	49	8230	9.34	2	1	1	1	1	0.18	0.63
RL14_MOUSE	60S ribosomal protein L14	49	23549	11.03	4	1	4	1	4	0.23	0.19
FABP4_MOUSE	Fatty acid-binding protein, adipocyte	49	14641	8.53	2	1	2	1	2	0.27	0.32
MCM7_MOUSE	DNA replication licensing factor MCM7	49	81160	5.98	6	2	6	2	6	0.16	0.11
S2611_MOUSE	Sodium-independent sulfate anion transporter	49	64068	7.56	4	3	2	1	2	0.08	0.07
UBQL1_MOUSE	Ubiquilin-1	49	61937	4.86	10	4	4	2	4	0.13	0.14
GSH0_MOUSE	Glutamate–cysteine ligase regulatory subunit	49	30516	5.35	2	1	2	1	2	0.17	0.15
PDK3_MOUSE	[Pyruvate dehydrogenase (acetyl-transferring)] kinase isozyme 3, mitochondrial	49	47893	8.89	1	1	1	1	1	0.04	0.09
P3H3_MOUSE	Prolyl 3-hydroxylase 3	48	81650	6.21	7	2	5	1	5	0.14	0.05
ARP10_MOUSE	Actin-related protein 10	48	46178	7.54	4	2	4	2	4	0.2	0.2
ARL2_MOUSE	ADP-ribosylation factor-like protein 2	48	20851	5.67	4	2	1	1	1	0.14	0.22
LAMA5_MOUSE	Laminin subunit alpha-5	48	403792	6.28	6	1	3	1	3	0.02	0.01
IFI5B_MOUSE	Interferon-activable protein 205-B	48	46948	8.2	11	2	8	2	8	0.27	0.19
NXN_MOUSE	Nucleoredoxin	48	48314	4.84	2	1	2	1	2	0.12	0.09
TTC27_MOUSE	Tetratricopeptide repeat protein 27	47	96369	5.56	9	3	4	2	4	0.11	0.09
GPAA1_MOUSE	Glycosylphosphatidylinositol anchor attachment 1 protein	47	67905	8.61	2	1	2	1	2	0.08	0.06
CP062_MOUSE	UPF0505 protein C16orf62 homolog	47	109007	6.97	7	1	5	1	5	0.08	0.04
CNOT1_MOUSE	CCR4-NOT transcription complex subunit 1	47	266637	6.65	8	2	8	2	8	0.07	0.03
NDUS3_MOUSE	NADH dehydrogenase [ubiquinone] iron-sulfur protein 3, mitochondrial	47	30131	6.67	4	1	3	1	3	0.18	0.15
CGNL1_MOUSE	Cingulin-like protein 1	47	148140	5.6	5	1	5	1	5	0.06	0.03
MCES_MOUSE	mRNA cap guanine-N7 methyltransferase	47	53258	6.12	4	2	3	1	3	0.09	0.08
EXOC7_MOUSE	Exocyst complex component 7	47	79910	6.51	6	2	4	1	4	0.14	0.05
GOGA3_MOUSE	Golgin subfamily A member 3	47	167118	5.29	7	1	7	1	7	0.1	0.03
IR3IP_MOUSE	Immediate early response 3-interacting protein 1	47	9011	7.96	2	1	2	1	2	0.32	0.56
CIP4_MOUSE	Cdc42-interacting protein 4	47	68447	5.66	1	1	1	1	1	0.02	0.06
GALT2_MOUSE	Polypeptide N-acetylgalactosaminyltransferase 2	46	64473	8.8	9	1	6	1	6	0.17	0.07
CD9_MOUSE	CD9 antigen	46	25241	6.88	5	4	2	1	2	0.26	0.18
NOP58_MOUSE	Nucleolar protein 58	46	60305	8.53	4	1	3	1	3	0.11	0.07
NIPS2_MOUSE	Protein NipSnap homolog 2	46	32912	9.31	4	2	2	2	2	0.15	0.29
AMPB_MOUSE	Aminopeptidase B	45	72370	5.22	7	3	6	3	6	0.22	0.19
NB5R1_MOUSE	NADH-cytochrome b5 reductase 1	45	34113	8.97	4	1	3	1	3	0.15	0.13
VPP2_MOUSE	V-type proton ATPase 116 kDa subunit a isoform 2	45	98081	6.2	3	1	2	1	2	0.05	0.04
IMPCT_MOUSE	Protein IMPACT	45	36253	4.97	9	2	4	2	4	0.24	0.26
MARE3_MOUSE	Microtubule-associated protein RP/EB family member 3	45	31946	5.33	2	1	2	1	2	0.17	0.14
TMM43_MOUSE	Transmembrane protein 43	45	44755	6.85	3	2	3	2	3	0.14	0.2
FA98B_MOUSE	Protein FAM98B	45	45321	8.77	4	1	3	1	3	0.19	0.1
ERP44_MOUSE	Endoplasmic reticulum resident protein 44	44	46823	5.09	2	1	2	1	2	0.1	0.09
TSP2_MOUSE	Thrombospondin-2	44	129798	4.61	9	2	6	2	5	0.1	0.07
HEM2_MOUSE	Delta-aminolevulinic acid dehydratase	44	36000	6.32	11	7	3	1	3	0.2	0.12
UROK_MOUSE	Urokinase-type plasminogen activator	44	48236	8.53	5	1	3	1	3	0.17	0.09
IF4G2_MOUSE	Eukaryotic translation initiation factor 4 gamma 2	44	102041	6.7	10	5	7	3	7	0.14	0.13
RSU1_MOUSE	Ras suppressor protein 1	44	31531	8.86	10	2	5	1	5	0.39	0.14
ITB5_MOUSE	Integrin beta-5	44	87851	5.81	8	3	8	3	8	0.22	0.15
SEP10_MOUSE	Septin-10	44	52388	6.17	8	2	5	1	5	0.15	0.08
EMC1_MOUSE	ER membrane protein complex subunit 1	44	111535	7	11	3	7	3	7	0.15	0.12
A16L1_MOUSE	Autophagy-related protein 16-1	43	68130	5.96	6	1	2	1	2	0.06	0.06
MINP1_MOUSE	Multiple inositol polyphosphate phosphatase 1	43	54503	7.21	3	2	2	1	2	0.09	0.08
MTAP_MOUSE	S-Methyl-5′-thioadenosine phosphorylase	43	31042	6.71	6	3	3	2	3	0.22	0.31
EMAL2_MOUSE	Echinoderm microtubule-associated protein-like 2	43	70689	5.83	5	2	2	1	2	0.07	0.06
ACADV_MOUSE	Very-long-chain-specific acyl-CoA dehydrogenase, mitochondrial	43	70831	8.91	9	1	6	1	6	0.19	0.06
AOFB_MOUSE	Amine oxidase [flavin-containing] B	43	58520	8.52	7	2	5	2	5	0.16	0.15
NRADD_MOUSE	Death domain-containing membrane protein NRADD	43	24711	4.89	3	3	1	1	1	0.14	0.4
DHSD_MOUSE	Succinate dehydrogenase [ubiquinone] cytochrome b small subunit, mitochondrial	43	17003	9.3	1	1	1	1	1	0.16	0.27
CSN9_MOUSE	COP9 signalosome complex subunit 9	43	6193	3.6	1	1	1	1	1	0.35	0.89
VP13C_MOUSE	Vacuolar protein sorting-associated protein 13C	43	419824	6.37	8	1	8	1	8	0.05	0.01
EMAL1_MOUSE	Echinoderm microtubule-associated protein-like 1	43	89624	6.56	10	2	8	2	8	0.21	0.1
TIP_MOUSE	T cell immunomodulatory protein	42	67422	5.4	6	1	2	1	2	0.09	0.06
CLU_MOUSE	Clustered mitochondria protein homolog	42	147975	5.69	2	2	2	2	2	0.03	0.06
PSME1_MOUSE	Proteasome activator complex subunit 1	40	28655	5.73	5	3	4	3	4	0.26	0.54
MFGM_MOUSE	Lactadherin	42	51208	6.1	8	3	6	3	6	0.19	0.28
E41L3_MOUSE	Band 4.1-like protein 3	42	103274	5.2	1	1	1	1	1	0.01	0.04
LMAN2_MOUSE	Vesicular integral-membrane protein VIP36	42	40404	6.46	6	2	5	2	5	0.23	0.23
PPM1F_MOUSE	Protein phosphatase 1F	42	49580	5.16	6	3	5	3	5	0.28	0.29
SERC3_MOUSE	Serine incorporator 3	42	52588	6.95	1	1	1	1	1	0.06	0.08
ARHG1_MOUSE	Rho guanine nucleotide exchange factor 1	42	102741	5.43	6	2	6	2	6	0.11	0.08
RS27A_MOUSE	Ubiquitin-40S ribosomal protein S27a	34	17939	9.68	7	2	2	2	2	0.22	0.58
MANEA_MOUSE	Glycoprotein endo-alpha-1,2-mannosidase	42	53149	8.66	1	1	1	1	1	0.03	0.08
MYO1B_MOUSE	Unconventional myosin-Ib	41	128483	9.32	3	1	2	1	2	0.04	0.03
PTCD3_MOUSE	Pentatricopeptide repeat domain-containing protein 3, mitochondrial	41	77747	5.61	4	2	3	1	3	0.08	0.06
COG2_MOUSE	Conserved oligomeric Golgi complex subunit 2	41	81988	5.84	2	1	2	1	2	0.09	0.05
KTN1_MOUSE	Kinectin	41	152498	5.67	7	2	7	2	7	0.1	0.06
ARXS1_MOUSE	Adipocyte-related X-chromosome expressed sequence 1	41	20134	9.6	2	1	2	1	2	0.14	0.23
RAB23_MOUSE	Ras-related protein Rab-23	41	26662	6.36	4	1	4	1	4	0.41	0.17
SMRC1_MOUSE	SWI/SNF complex subunit SMARCC1	41	122813	5.5	5	1	5	1	4	0.08	0.03
HGNAT_MOUSE	Heparan-alpha-glucosaminide N-acetyltransferase	41	72458	8.58	2	2	1	1	1	0.05	0.06
GGCT_MOUSE	Gamma-glutamylcyclotransferase	41	21152	5.52	3	1	3	1	3	0.31	0.21
PLPP1_MOUSE	Phospholipid phosphatase 1	41	31871	6.54	6	3	3	2	3	0.16	0.3
RFOX2_MOUSE	RNA binding protein fox-1 homolog 2	40	47301	6.1	3	1	3	1	3	0.1	0.09
DPP9_MOUSE	Dipeptidyl peptidase 9	40	97939	6.18	3	1	3	1	3	0.03	0.04
CD63_MOUSE	CD63 antigen	40	25749	6.69	3	2	1	1	1	0.05	0.17
ARFG1_MOUSE	ADP-ribosylation factor GTPase-activating protein 1	40	45260	5.39	2	1	2	1	2	0.11	0.1
NOP16_MOUSE	Nucleolar protein 16	40	21126	9.91	1	1	1	1	1	0.1	0.22
SNRPA_MOUSE	U1 small nuclear ribonucleoprotein A	40	31814	9.81	3	1	3	1	3	0.18	0.14
KIF1C_MOUSE	Kinesin-like protein KIF1C	40	122358	6.62	6	1	5	1	5	0.1	0.03
LRC40_MOUSE	Leucine-rich repeat-containing protein 40	40	68033	6.43	3	1	3	1	3	0.1	0.06
MAOM_MOUSE	NAD-dependent malic enzyme, mitochondrial	40	65757	7.53	2	2	2	2	2	0.08	0.14
LAMB1_MOUSE	Laminin subunit beta-1	40	196961	4.82	13	2	6	1	6	0.08	0.02
GTR1_MOUSE	Solute carrier family 2, facilitated glucose transporter member 1	40	53949	9.05	5	3	4	2	4	0.13	0.17
CKAP5_MOUSE	Cytoskeleton-associated protein 5	40	225492	8.17	11	2	9	2	9	0.08	0.04
LYAR_MOUSE	Cell growth-regulating nucleolar protein	40	43708	9.52	4	1	3	1	3	0.1	0.1
SAAL1_MOUSE	Protein SAAL1	40	52735	4.4	4	1	4	1	4	0.2	0.08
WDR12_MOUSE	Ribosome biogenesis protein WDR12	40	47317	5.36	2	1	1	1	1	0.08	0.09
TBC15_MOUSE	TBC1 domain family member 15	39	76478	5.15	2	2	1	1	1	0.04	0.06
PGLT1_MOUSE	Protein O-glucosyltransferase 1	39	46350	9.04	2	2	2	2	2	0.11	0.2
ACOD2_MOUSE	Acyl-CoA desaturase 2	39	40890	9.14	2	1	2	1	2	0.12	0.11
ARHG2_MOUSE	Rho guanine nucleotide exchange factor 2	39	111905	6.89	3	2	2	1	2	0.06	0.04
NFKB2_MOUSE	Nuclear factor NF-kappa-B p100 subunit	39	96772	5.93	4	3	2	2	2	0.06	0.09
NU188_MOUSE	Nucleoporin NUP188 homolog	39	196570	6.56	5	1	4	1	4	0.05	0.02
MCA3_MOUSE	Eukaryotic translation elongation factor 1 epsilon-1	39	19846	8.6	4	2	3	1	3	0.33	0.23
STING_MOUSE	Stimulator of interferon genes protein	39	42802	7.12	6	3	4	2	4	0.23	0.21
5NT3A_MOUSE	Cytosolic 5′-nucleotidase 3A	39	37228	6.21	2	1	2	1	2	0.15	0.12
NDUV3_MOUSE	NADH dehydrogenase [ubiquinone] flavoprotein 3, mitochondrial	39	11806	9.34	3	2	2	1	2	0.32	0.41
MTX1_MOUSE	Metaxin-1	39	35601	5.82	2	2	1	1	1	0.04	0.12
EMC3_MOUSE	ER membrane protein complex subunit 3	39	29960	6.33	4	3	2	1	2	0.16	0.15
TULP3_MOUSE	Tubby-related protein 3	38	51199	5.88	3	1	2	1	2	0.12	0.08
SEC63_MOUSE	Translocation protein SEC63 homolog	38	87815	5.28	8	3	5	3	5	0.09	0.15
TFAM_MOUSE	Transcription factor A, mitochondrial	38	27970	9.71	1	1	1	1	1	0.06	0.16
TMX4_MOUSE	Thioredoxin-related transmembrane protein 4	38	37108	4.3	2	1	1	1	1	0.05	0.12
SP16H_MOUSE	FACT complex subunit SPT16	38	119749	5.5	7	3	4	2	4	0.08	0.07
ACADS_MOUSE	Short-chain specific acyl-CoA dehydrogenase, mitochondrial	38	44861	8.68	2	2	1	1	1	0.08	0.1
HEXB_MOUSE	Beta-hexosaminidase subunit beta	38	61077	8.29	4	2	2	1	2	0.11	0.07
GBP2_MOUSE	Guanylate-binding protein 2	38	66697	5.56	2	2	1	1	1	0.04	0.06
SK2L2_MOUSE	Superkiller viralicidic activity 2-like 2	37	117561	6.01	10	2	8	1	8	0.16	0.04
PFKAP_MOUSE	ATP-dependent 6-phosphofructokinase, platelet type	37	85400	6.73	9	1	8	1	8	0.18	0.05
T2FB_MOUSE	General transcription factor IIF subunit 2	37	28364	9.23	3	1	2	1	2	0.11	0.16
SAM50_MOUSE	Sorting and assembly machinery component 50 homolog	37	51831	6.34	3	1	3	1	3	0.12	0.08
XRCC6_MOUSE	X-ray repair cross-complementing protein 6	37	69441	6.35	1	1	1	1	1	0.03	0.06
KBL_MOUSE	2-Amino-3-ketobutyrate coenzyme A ligase, mitochondrial	37	44902	6.92	1	1	1	1	1	0.06	0.1
PRI2_MOUSE	DNA primase large subunit	37	58372	8.47	3	1	3	1	3	0.13	0.07
TGM2_MOUSE	Protein-glutamine gamma-glutamyltransferase 2	37	77012	4.98	2	2	2	2	2	0.07	0.11
SPART_MOUSE	Spartin	37	72610	5.64	4	1	3	1	3	0.12	0.06
SRRM1_MOUSE	Serine/arginine repetitive matrix protein 1	37	106798	11.87	4	1	2	1	2	0.04	0.04
CSPG4_MOUSE	Chondroitin sulfate proteoglycan 4	37	252153	5.23	8	2	7	2	7	0.07	0.03
DHX29_MOUSE	ATP-dependent RNA helicase DHX29	36	153879	8.13	3	1	3	1	3	0.03	0.03
DECR_MOUSE	2,4-Dienoyl-CoA reductase, mitochondrial	36	36191	9.1	6	2	5	1	5	0.22	0.12
CHKB_MOUSE	Choline/ethanolamine kinase	36	45097	5.3	3	1	2	1	2	0.12	0.1
RLA1_MOUSE	60S acidic ribosomal protein P1	36	11468	4.28	2	2	1	1	1	0.14	0.43
MBD3_MOUSE	Methyl-CpG-binding domain protein 3	36	32148	5.64	3	1	2	1	2	0.11	0.14
IF6_MOUSE	Eukaryotic translation initiation factor 6	36	26494	4.63	2	1	2	1	2	0.26	0.17
NISCH_MOUSE	Nischarin	36	174903	5.05	3	2	3	2	3	0.04	0.05
CD44_MOUSE	CD44 antigen	36	85565	4.82	4	3	2	2	2	0.05	0.1
NPM3_MOUSE	Nucleoplasmin-3	36	19011	4.71	1	1	1	1	1	0.19	0.24
SNX2_MOUSE	Sorting nexin-2	35	58435	5.04	2	1	2	1	2	0.1	0.07
MRP1_MOUSE	Multidrug resistance-associated protein 1	35	171075	7.03	5	1	5	1	5	0.07	0.02
SERB_MOUSE	Phosphoserine phosphatase	35	25080	5.81	4	2	3	2	3	0.24	0.39
SMYD5_MOUSE	SET and MYND domain-containing protein 5	35	47065	5.09	2	1	1	1	1	0.06	0.09
PGFRB_MOUSE	Platelet-derived growth factor receptor beta	35	122728	4.99	8	2	6	2	6	0.09	0.07
APAF_MOUSE	Apoptotic protease-activating factor 1	35	140913	5.98	6	1	3	1	3	0.04	0.03
PLS3_MOUSE	Phospholipid scramblase 3	35	31782	5.97	6	2	3	2	3	0.11	0.3
NMRL1_MOUSE	NmrA-like family domain-containing protein 1	35	34355	6.37	3	1	2	1	2	0.15	0.13
EHD2_MOUSE	EH domain-containing protein 2	35	61136	6.08	10	3	6	3	6	0.21	0.23
CRKL_MOUSE	Crk-like protein	34	33809	6.26	9	2	4	1	4	0.23	0.13
EI2BB_MOUSE	Translation initiation factor eIF-2B subunit beta	34	38873	5.82	4	2	3	1	3	0.22	0.11
BLVRB_MOUSE	Flavin reductase (NADPH)	34	22183	6.49	7	1	4	1	4	0.38	0.2
SRSF6_MOUSE	Serine/arginine-rich splicing factor 6	34	39002	11.46	2	1	1	1	1	0.07	0.11
TRI47_MOUSE	Tripartite motif-containing protein 47	34	69868	6	2	2	1	1	1	0.05	0.06
ICLN_MOUSE	Methylosome subunit pICln	34	26005	3.98	2	1	2	1	2	0.15	0.17
GNS_MOUSE	N-Acetylglucosamine-6-sulfatase	34	61136	8.52	16	2	9	2	9	0.35	0.15
PLBL2_MOUSE	Putative phospholipase B-like 2	34	66247	5.77	5	1	4	1	4	0.12	0.07
TBCA_MOUSE	Tubulin-specific chaperone A	34	12750	5.24	3	1	2	1	2	0.37	0.38
SGPL1_MOUSE	Sphingosine-1-phosphate lyase 1	34	63636	9.2	6	1	5	1	5	0.11	0.07
T126A_MOUSE	Transmembrane protein 126A	34	21526	9.45	2	1	2	1	2	0.17	0.21
DHR11_MOUSE	Dehydrogenase/reductase SDR family member 11	34	28256	5.91	3	2	2	1	2	0.17	0.16
KTAP2_MOUSE	Keratinocyte-associated protein 2	34	14665	9.67	3	1	1	1	1	0.2	0.32
LAMC1_MOUSE	Laminin subunit gamma-1	33	177185	5.08	3	1	3	1	3	0.04	0.02
3HIDH_MOUSE	3-Hydroxyisobutyrate dehydrogenase, mitochondrial	33	35417	8.37	3	1	2	1	2	0.19	0.12
BGAL_MOUSE	Beta-galactosidase	33	73074	7.15	2	1	2	1	2	0.06	0.06
FKBP8_MOUSE	Peptidyl-prolyl cis-trans isomerase FKBP8	33	43501	5.08	2	1	2	1	2	0.11	0.1
CASP3_MOUSE	Caspase-3	33	31454	6.45	4	1	3	1	3	0.24	0.14
RT33_MOUSE	28S ribosomal protein S33, mitochondrial	33	12452	10.25	1	1	1	1	1	0.2	0.39
ATX2L_MOUSE	Ataxin-2-like protein	33	110580	8.94	3	1	3	1	3	0.07	0.04
SELB_MOUSE	Selenocysteine-specific elongation factor	33	63498	8.58	2	1	2	1	2	0.09	0.07
TRI16_MOUSE	Tripartite motif-containing protein 16	33	62903	5.57	2	1	2	1	2	0.06	0.07
ARSA_MOUSE	Arylsulfatase A	33	53714	5.5	3	1	2	1	2	0.1	0.08
BL1S6_MOUSE	Biogenesis of lysosome-related organelles complex 1 subunit 6	33	19670	5.91	2	1	1	1	1	0.11	0.23
ECH1_MOUSE	Delta(3,5)-delta(2,4)-dienoyl-CoA isomerase, mitochondrial	33	36095	7.6	4	1	4	1	4	0.31	0.12
DIAP3_MOUSE	Protein diaphanous homolog 3	32	133601	7.53	3	1	3	1	3	0.05	0.03
ERG1_MOUSE	Squalene monooxygenase	32	63730	8.67	5	1	2	1	2	0.1	0.07
DDX46_MOUSE	Probable ATP-dependent RNA helicase DDX46	32	117376	9.3	4	1	3	1	3	0.05	0.04
M4K4_MOUSE	Mitogen-activated protein kinase 4	32	140515	7.1	7	1	7	1	7	0.1	0.03
ABCD3_MOUSE	ATP-binding cassette sub-family D member 3	32	75426	9.32	9	1	7	1	7	0.13	0.06
PSMG1_MOUSE	Proteasome assembly chaperone 1	32	33083	6.05	4	2	2	1	2	0.12	0.29
FRDA_MOUSE	Frataxin, mitochondrial	32	22910	7.81	1	1	1	1	1	0.08	0.2
THOP1_MOUSE	Thimet oligopeptidase	32	77976	5.72	3	2	2	1	2	0.06	0.06
CREL2_MOUSE	Cysteine-rich with EGF-like domain protein 2	32	38194	4.49	3	1	2	1	2	0.05	0.11
SSRD_MOUSE	Translocon-associated protein subunit delta	32	18924	5.5	4	1	4	1	4	0.31	0.24
I2BP2_MOUSE	Interferon regulatory factor 2-binding protein 2	32	59255	9	6	1	5	1	5	0.18	0.07
RL29_MOUSE	60S ribosomal protein L29	31	17576	11.84	2	1	2	1	2	0.17	0.26
B4GT1_MOUSE	Beta-1,4-galactosyltransferase 1	31	44383	9.45	3	1	2	1	2	0.07	0.1
HTR5A_MOUSE	HEAT repeat-containing protein 5A	31	219754	6.31	6	1	5	1	5	0.06	0.02
SYFB_MOUSE	Phenylalanine–tRNA ligase beta subunit	31	65655	6.69	4	2	3	1	3	0.09	0.07
BAG5_MOUSE	BAG family molecular chaperone regulator 5	31	50911	5.75	3	1	3	1	3	0.14	0.09
HSPB8_MOUSE	Heat shock protein beta-8	31	21520	4.92	1	1	1	1	1	0.13	0.21
SNX7_MOUSE	Sorting nexin-7	31	44971	4.99	4	2	2	2	2	0.12	0.2
TMTC3_MOUSE	Transmembrane and TPR repeat-containing protein 3	31	104131	8.75	5	2	4	1	4	0.06	0.04
NNMT_MOUSE	Nicotinamide N-methyltransferase	31	29579	5.3	4	3	2	2	2	0.16	0.32
WIPI1_MOUSE	WD repeat domain phosphoinositide-interacting protein 1	30	48727	6.03	3	2	2	1	2	0.13	0.09
K1468_MOUSE	LisH domain and HEAT repeat-containing protein KIAA1468	30	134502	5.2	7	1	5	1	5	0.09	0.03
FKBP3_MOUSE	Peptidyl-prolyl cis-trans isomerase FKBP3	30	25132	9.29	6	2	6	2	6	0.42	0.39
FHL2_MOUSE	Four and a half LIM domains protein 2	30	32051	7.31	2	1	2	1	2	0.15	0.14
ARI4B_MOUSE	AT-rich interactive domain-containing protein 4B	30	147553	4.98	4	1	3	1	3	0.03	0.03
ANX11_MOUSE	Annexin A11	29	54045	7.53	2	1	1	1	1	0.05	0.08
RM48_MOUSE	39S ribosomal protein L48, mitochondrial	29	23938	9.45	3	1	1	1	1	0.13	0.19
SEN34_MOUSE	tRNA-splicing endonuclease subunit Sen34	29	34176	6.56	2	1	2	1	2	0.13	0.13
HP1B3_MOUSE	Heterochromatin protein 1-binding protein 3	29	60829	9.71	5	2	4	2	4	0.16	0.15
COX8A_MOUSE	Cytochrome c oxidase subunit 8A, mitochondrial	29	7643	9.74	3	1	1	1	1	0.49	0.69
LSM7_MOUSE	U6 snRNA-associated Sm-like protein LSm7	29	11629	5.1	4	2	1	1	1	0.24	0.42
CND1_MOUSE	Condensin complex subunit 1	29	155567	5.98	2	1	2	1	2	0.03	0.03
ARSB_MOUSE	Arylsulfatase B	29	59609	6.78	2	1	1	1	1	0.05	0.07
RBP2_MOUSE	E3 SUMO-protein ligase RanBP2	29	340907	5.82	7	1	7	1	7	0.04	0.01
SRPK1_MOUSE	SRSF protein kinase 1	29	73043	5.82	2	1	1	1	1	0.02	0.06
VAC14_MOUSE	Protein VAC14 homolog	29	87992	5.75	3	2	2	1	2	0.05	0.05
UBR2_MOUSE	E3 ubiquitin-protein ligase UBR2	29	199026	5.9	8	1	5	1	5	0.06	0.02
NCEH1_MOUSE	Neutral cholesterol ester hydrolase 1	29	45711	6.56	3	1	1	1	1	0.07	0.1
LMAN1_MOUSE	Protein ERGIC-53	29	57753	5.92	7	2	5	2	5	0.21	0.16
DREB_MOUSE	Drebrin	29	77239	4.45	2	1	2	1	2	0.06	0.06
AURKB_MOUSE	Aurora kinase B	29	39360	9.47	1	1	1	1	1	0.05	0.11
STOM_MOUSE	Erythrocyte band 7 integral membrane protein	29	31355	6.45	2	1	2	1	2	0.17	0.14
SETD3_MOUSE	Histone-lysine N-methyltransferase setd3	29	67134	5.47	4	2	2	2	2	0.08	0.13
DAAM1_MOUSE	Disheveled-associated activator of morphogenesis 1	29	123293	7.12	6	1	5	1	5	0.08	0.03
IF2B2_MOUSE	Insulin-like growth factor 2 mRNA-binding protein 2	28	65543	7.81	5	1	4	1	4	0.1	0.07
GSDMD_MOUSE	Gasdermin-D	28	53204	5.03	4	2	3	2	3	0.17	0.17
ACON_MOUSE	Aconitate hydratase, mitochondrial	28	85410	8.08	8	2	5	2	5	0.12	0.1
IF4B_MOUSE	Eukaryotic translation initiation factor 4B	28	68799	5.47	3	1	2	1	2	0.09	0.06
XRCC5_MOUSE	X-ray repair cross-complementing protein 5	28	83004	5.04	5	1	5	1	5	0.16	0.05
GALE_MOUSE	UDP-glucose 4-epimerase	28	38200	6.27	5	2	2	1	2	0.14	0.11
SYMPK_MOUSE	Symplekin	28	142194	5.76	11	1	6	1	6	0.07	0.03
NAA10_MOUSE	N-Alpha-acetyltransferase 10	28	26503	5.41	8	3	3	1	3	0.28	0.37
GALD1_MOUSE	Glutamine amidotransferase-like class 1 domain-containing protein 1	28	23263	6.58	1	1	1	1	1	0.09	0.19
ZO1_MOUSE	Tight junction protein ZO-1	28	194622	6.17	6	1	6	1	6	0.07	0.02
PIGS_MOUSE	GPI transamidase component PIG-S	27	61671	6.45	5	2	3	1	3	0.13	0.07
NRP1_MOUSE	Neuropilin-1	27	102935	5.6	3	1	3	1	3	0.07	0.04
LEMD2_MOUSE	LEM domain-containing protein 2	27	57471	9.18	1	1	1	1	1	0.05	0.08
RTCA_MOUSE	RNA 3′-terminal phosphate cyclase	27	39229	8.06	4	1	2	1	2	0.13	0.11
SULF1_MOUSE	Extracellular sulfatase Sulf-1	27	100858	9.17	4	1	3	1	3	0.04	0.04
UFC1_MOUSE	Ubiquitin-fold modifier-conjugating enzyme 1	27	19469	6.9	5	1	3	1	3	0.28	0.24
IMPA3_MOUSE	Inositol monophosphatase 3	27	38592	6.03	2	1	2	1	2	0.14	0.11
RINT1_MOUSE	RAD50-interacting protein 1	27	90036	5.02	5	1	4	1	4	0.06	0.05
KCY_MOUSE	UMP-CMP kinase	26	22151	5.68	4	1	4	1	4	0.28	0.2
TRIP6_MOUSE	Thyroid receptor-interacting protein 6	26	50901	7.03	4	1	2	1	2	0.09	0.09
PLXB2_MOUSE	Plexin-B2	26	206099	5.58	7	1	6	1	6	0.07	0.02
UFL1_MOUSE	E3 UFM1-protein ligase 1	26	89464	6.24	4	1	3	1	3	0.1	0.05
SMRC2_MOUSE	SWI/SNF complex subunit SMARCC2	26	132522	5.41	4	1	4	1	3	0.07	0.03
PPOX_MOUSE	Protoporphyrinogen oxidase	26	50839	9.07	1	1	1	1	1	0.06	0.09
PDLI7_MOUSE	PDZ and LIM domain protein 7	26	50087	8.82	1	1	1	1	1	0.07	0.09
CLN3_MOUSE	Battenin	26	47627	5.25	1	1	1	1	1	0.05	0.09
TRM61_MOUSE	tRNA (adenine(58)-N(1))-methyltransferase catalytic subunit TRMT61A	26	31619	6.3	1	1	1	1	1	0.07	0.14
TBRG4_MOUSE	Protein TBRG4	26	71468	8.55	2	1	2	1	2	0.04	0.06
AN13A_MOUSE	Ankyrin repeat domain-containing protein 13A	26	67136	4.99	1	1	1	1	1	0.03	0.06
TMED3_MOUSE	Transmembrane emp24 domain-containing protein 3	26	25449	5.62	2	1	2	1	2	0.16	0.18
NPL4_MOUSE	Nuclear protein localization protein 4 homolog	26	67974	6.01	4	1	4	1	4	0.13	0.06
SAP18_MOUSE	Histone deacetylase complex subunit SAP18	26	17584	9.38	2	1	2	1	2	0.24	0.26
SUMF2_MOUSE	Sulfatase-modifying factor 2	26	34715	6.61	1	1	1	1	1	0.06	0.13
SYMC_MOUSE	Methionine–tRNA ligase, cytoplasmic	25	101366	6.78	10	1	8	1	8	0.15	0.04
NOL6_MOUSE	Nucleolar protein 6	25	129146	6.33	3	1	3	1	3	0.07	0.03
CRIP2_MOUSE	Cysteine-rich protein 2	25	22712	8.94	4	1	4	1	4	0.39	0.2
GSKIP_MOUSE	GSK3B-interacting protein	25	15632	4.3	3	1	1	1	1	0.17	0.3
STAR5_MOUSE	StAR-related lipid transfer protein 5	25	23907	5.97	3	1	3	1	3	0.18	0.19
LARP4_MOUSE	La-related protein 4	25	79713	6.02	2	1	2	1	2	0.06	0.05
OFUT1_MOUSE	GDP-fucose protein O-fucosyltransferase 1	25	44660	8.67	1	1	1	1	1	0.05	0.1
RHG10_MOUSE	Rho GTPase-activating protein 10	25	89309	6.75	6	1	4	1	4	0.1	0.05
SRA1_MOUSE	Steroid receptor RNA activator 1	25	25541	6.01	2	1	2	1	2	0.17	0.18
COR1B_MOUSE	Coronin-1B	25	53878	5.54	9	1	6	1	6	0.18	0.08
NIF3L_MOUSE	NIF3-like protein 1	25	41719	6.28	4	2	1	1	1	0.05	0.1
LYOX_MOUSE	Protein-lysine 6-oxidase	25	46671	8.73	3	1	1	1	1	0.08	0.09
GSH1_MOUSE	Glutamate–cysteine ligase catalytic subunit	25	72525	5.59	8	2	3	1	3	0.09	0.06
GIT2_MOUSE	ARF GTPase-activating protein GIT2	25	78717	7.64	5	1	5	1	5	0.12	0.05
BL1S2_MOUSE	Biogenesis of lysosome-related organelles complex 1 subunit 2	24	16289	4.82	3	2	1	1	1	0.19	0.29
IBP7_MOUSE	Insulin-like growth factor-binding protein 7	24	28951	8.71	1	1	1	1	1	0.07	0.15
PLIN3_MOUSE	Perilipin-3	24	47233	5.45	8	2	4	2	4	0.24	0.19
TM9S2_MOUSE	Transmembrane 9 superfamily member 2	24	75280	7.21	5	2	2	1	2	0.09	0.06
CAP2_MOUSE	Adenylyl cyclase-associated protein 2	24	52829	6	3	1	2	1	2	0.06	0.08
LPP_MOUSE	Lipoma-preferred partner homolog	24	65848	7.19	7	1	5	1	5	0.17	0.07
STXB3_MOUSE	Syntaxin-binding protein 3	24	67899	8.28	5	1	3	1	3	0.12	0.06
EDC4_MOUSE	Enhancer of mRNA-decapping protein 4	23	152389	5.51	5	1	4	1	4	0.06	0.03
CO6A2_MOUSE	Collagen alpha-2(VI) chain	23	110266	6.01	2	1	2	1	2	0.04	0.04
ATG4B_MOUSE	Cysteine protease ATG4B	23	44347	4.93	1	1	1	1	1	0.04	0.1
GGPPS_MOUSE	Geranylgeranyl pyrophosphate synthase	23	34685	6.03	2	1	1	1	1	0.09	0.13
TT39B_MOUSE	Tetratricopeptide repeat protein 39B	23	70248	6.22	3	1	2	1	2	0.06	0.06
VPS11_MOUSE	Vacuolar protein sorting-associated protein 11 homolog	22	107650	6.54	3	1	2	1	2	0.05	0.04
KANK2_MOUSE	KN motif and ankyrin repeat domain-containing protein 2	22	90190	5.38	2	1	2	1	2	0.06	0.05
ANO6_MOUSE	Anoctamin-6	22	106186	6.34	2	1	1	1	1	0.01	0.04
ACAD8_MOUSE	Isobutyryl-CoA dehydrogenase, mitochondrial	22	44990	8.46	1	1	1	1	1	0.06	0.1
BASI_MOUSE	Basigin	22	42418	5.56	3	1	1	1	1	0.04	0.1
RO60_MOUSE	60 kDa SS-A/Ro ribonucleoprotein	22	60085	8.11	4	1	3	1	3	0.09	0.07
AHSA2_MOUSE	Activator of 90 kDa heat shock protein ATPase homolog 2	22	37624	6.1	2	1	2	1	2	0.11	0.12
CND2_MOUSE	Condensin complex subunit 2	21	82251	4.81	2	1	2	1	2	0.05	0.05
TM214_MOUSE	Transmembrane protein 214	21	76381	9.41	3	1	2	1	2	0.06	0.06
MPC1_MOUSE	Mitochondrial pyruvate carrier 1	21	12446	9.67	3	1	1	1	1	0.19	0.39
PIGT_MOUSE	GPI transamidase component PIG-T	21	65663	8.68	1	1	1	1	1	0.03	0.07
UBP4_MOUSE	Ubiquitin carboxyl-terminal hydrolase 4	21	108274	5.42	6	2	3	1	3	0.09	0.04
DNLI1_MOUSE	DNA ligase 1	21	102226	6.43	1	1	1	1	1	0.02	0.04
FUCO_MOUSE	Tissue alpha-L-fucosidase	20	52247	6.47	1	1	1	1	1	0.04	0.08
CEBPZ_MOUSE	CCAAT/enhancer-binding protein zeta	20	120187	5.53	5	1	3	1	3	0.08	0.04
E2AK2_MOUSE	Interferon-induced, double-stranded RNA-activated protein kinase	20	58243	8.76	4	1	3	1	3	0.12	0.07
AFG31_MOUSE	AFG3-like protein 1	20	86992	9	1	1	1	1	1	0.02	0.05
RICTR_MOUSE	Rapamycin-insensitive companion of mTOR	20	191449	6.8	9	1	5	1	5	0.05	0.02
NOC4L_MOUSE	Nucleolar complex protein 4 homolog	19	58638	6.29	3	1	1	1	1	0.04	0.07
SOAT1_MOUSE	Sterol O-acyltransferase 1	19	63757	9.15	6	1	4	1	4	0.15	0.07
SH3G1_MOUSE	Endophilin-A2	19	41492	5.53	2	1	1	1	1	0.04	0.11
ILVBL_MOUSE	Acetolactate synthase-like protein	19	68113	8.96	2	1	2	1	2	0.09	0.06
LASP1_MOUSE	LIM and SH3 domain protein 1	19	29975	6.61	6	1	5	1	5	0.19	0.15
NUMA1_MOUSE	Nuclear mitotic apparatus protein 1	18	235487	5.68	10	1	10	1	10	0.08	0.02
DPP8_MOUSE	Dipeptidyl peptidase 8	18	102121	5.51	3	1	2	1	2	0.04	0.04
MTX2_MOUSE	Metaxin-2	18	29739	5.44	1	1	1	1	1	0.11	0.15
RM15_MOUSE	39S ribosomal protein L15, mitochondrial	18	33521	10.08	2	1	2	1	2	0.1	0.13
PM34_MOUSE	Peroxisomal membrane protein PMP34	17	34391	10.11	1	1	1	1	1	0.07	0.13
PPP5_MOUSE	Serine/threonine-protein phosphatase 5	17	56840	5.83	4	1	4	1	4	0.17	0.08
DOK1_MOUSE	Docking protein 1	16	52419	6.16	4	1	3	1	3	0.14	0.08
LEG9_MOUSE	Galectin-9	16	40010	9.41	3	1	1	1	1	0.06	0.11
PGM2_MOUSE	Phosphoglucomutase-2	16	68704	5.78	2	1	2	1	2	0.08	0.06
HSP7E_MOUSE	Heat shock 70 kDa protein 14	16	54616	5.63	4	1	4	1	4	0.17	0.08
PUM2_MOUSE	Pumilio homolog 2	15	114243	6.61	3	1	3	1	3	0.08	0.04

^a^Protein score is calculated from the score of the peptide attributed to the protein. ^b^pI is (predicted) isoelectric point. ^c^Number of matches is spectrum number matched to protein^#1^. ^d^Number of significant matches is spectrum number that matches protein and exceeds the identification criteria. ^e^Number of sequences is number of peptides matched to protein^#2^. ^f^Number of significant sequences is number of peptides exceeding the identification criteria matched to proteins. ^g^Number of unique sequences is a unique^#3^ number of peptides matched to proteins. ^h^Sequence coverage is the ratio of the total number of matched peptide residues to the total length of the protein. ^i^Exponentially modified protein abundance index (http://www.matrixscience.com/help/quant_empai_help.html). ^#1^When multiple spectra are matched to the same peptide, the way of counting into one is called "peptide number". ^#2^When multiple spectra are matched to the same peptide, the method of counting is called "spectrum number". ^#3^"Unique" is a peptide that is not matched to other proteins and has been assigned only to the relevant protein.

**Table 3:** Identification of endogenous proteins contained in mMSC-AT_P0 only, not in mMSC-AT_P3.

Exclusive P0 group		
UniProt/Swiss-Prot ID	Description	Molecular function
FABP5_MOUSE	Fatty acid-binding protein, epidermal	Fatty acid binding, transporter activity
AMPN_MOUSE	Aminopeptidase N	Metal ion binding, metalloaminopeptidase activity, metallopeptidase activity, peptide binding, zinc ion binding
AOFA_MOUSE	Amine oxidase [flavin-containing] A	Oxidoreductase activity, primary amine oxidase activity, protein binding
HXK3_MOUSE	Hexokinase-3	ATP binding, fructokinase activity, glucokinase activity, glucose binding, kinase activity, mannokinase activity, nucleotide binding
CTGF_MOUSE	Connective tissue growth factor	Fibronectin binding, growth factor activity, heparin binding, insulin-like growth factor binding, integrin binding, protein C-terminus binding
THIL_MOUSE	Acetyl-CoA acetyltransferase, mitochondrial	Acetyl-CoA C-acetyltransferase activity, carbon-carbon lyase activity, coenzyme binding, enzyme binding, ligase activity, forming carbon-carbon bonds, metal ion binding, protein homodimerization activity, transferase activity, transferring acyl groups
ATPD_MOUSE	ATP synthase subunit delta, mitochondrial	ATPase activity, hydrogen ion transmembrane transporter activity, proton-transporting ATP synthase activity, rotational mechanism
CATS_MOUSE	Cathepsin S	Collagen binding, cysteine-type endopeptidase activity, cysteine-type peptidase activity, fibronectin binding, hydrolase activity, laminin binding, proteoglycan binding
CYR61_MOUSE	Protein CYR61	Extracellular matrix binding, growth factor binding, heparin binding, insulin-like growth factor binding, integrin binding
ETFB_MOUSE	Electron transfer flavoprotein subunit beta	Electron carrier activity
SFXN3_MOUSE	Sideroflexin-3	Ion transmembrane transporter activity, molecular function
STML2_MOUSE	Stomatin-like protein 2, mitochondrial	GTPase binding, cardiolipin binding, lipid binding
RDH11_MOUSE	Retinol dehydrogenase 11	NADP-retinol dehydrogenase activity, retinol dehydrogenase activity
NSF1C_MOUSE	NSFL1 cofactor p47	ATPase binding, lipid binding, phospholipid binding, ubiquitin binding
BAG3_MOUSE	BAG family molecular chaperone regulator 3	Adenyl-nucleotide exchange factor activity, cadherin binding involved in cell-cell adhesion, chaperone binding, protein complex binding
CSF1_MOUSE	Macrophage colony-stimulating factor 1	Cytokine activity, growth factor activity, macrophage colony-stimulating factor receptor binding, protein homodimerization activity
LYZ2_MOUSE	Lysozyme C-2	Hydrolase activity, identical protein binding, lysozyme activity
VATC1_MOUSE	V-type proton ATPase subunit C 1	Hydrogen-exporting ATPase activity, phosphorylative mechanism
UBP2L_MOUSE	Ubiquitin-associated protein 2-like	RNA binding
RL28_MOUSE	60S ribosomal protein L28	RNA binding, structural constituent of ribosome
ACSL4_MOUSE	Long-chain-fatty-acid–CoA ligase 4	ATP binding, arachidonate-CoA ligase activity, decanoate–CoA ligase activity, ligase activity, long-chain fatty acid-CoA ligase activity, nucleotide binding, very-long-chain fatty acid-CoA ligase activity
THIKA_MOUSE	3-Ketoacyl-CoA thiolase A, peroxisomal	Acetate CoA-transferase activity, acetyl-CoA C-acetyltransferase activity, acetyl-CoA C-acyltransferase activity, palmitoyl-CoA oxidase activity
FA49B_MOUSE	Protein FAM49B	Protein binding
CO5A2_MOUSE	Collagen alpha-2(V) chain	SMAD binding, extracellular matrix structural constituent, metal ion binding
DCTN2_MOUSE	Dynactin subunit 2	Motor activity, spectrin binding
SUCB1_MOUSE	Succinate–CoA ligase [ADP-forming] subunit beta, mitochondrial	ATP binding, ligase activity, metal ion binding, nucleotide binding, succinate-CoA ligase (ADP-forming) activity
SYUG_MOUSE	Gamma-synuclein	Protein binding
GUAD_MOUSE	Guanine deaminase	Guanine deaminase activity, hydrolase activity, metal ion binding, zinc ion binding
ISG15_MOUSE	Ubiquitin-like protein ISG15	Protein binding, protein tag
STK24_MOUSE	Serine/threonine-protein kinase 24	ATP binding, cadherin binding involved in cell-cell adhesion, metal ion binding, nucleotide binding, signal transducer, downstream of receptor, with serine/threonine kinase activity, transferase activity
STX7_MOUSE	Syntaxin-7	SNAP receptor activity, chloride channel inhibitor activity, syntaxin binding
EFHD2_MOUSE	EF-hand domain-containing protein D2	Cadherin binding involved in cell-cell adhesion, calcium ion binding, metal ion binding
NECP2_MOUSE	Adaptin ear-binding coat-associated protein 2	Molecular function
ATP5J_MOUSE	ATP synthase-coupling factor 6, mitochondrial	ATPase activity, hydrogen ion transmembrane transporter activity
ERH_MOUSE	Enhancer of rudimentary homolog	RNA binding, methyl-CpG binding
HAP28_MOUSE	28 kDa heat- and acid-stable phosphoprotein	RNA binding, platelet-derived growth factor binding
TOM20_MOUSE	Mitochondrial import receptor subunit TOM20 homolog	P-P-bond-hydrolysis-driven protein transmembrane transporter activity, mitochondrion targeting sequence binding, protein channel activity, unfolded protein binding
CD109_MOUSE	CD109 antigen	Serine-type endopeptidase inhibitor activity, transforming growth factor beta binding
CD36_MOUSE	Platelet glycoprotein 4	High-density lipoprotein particle binding, lipid binding, lipoteichoic acid receptor activity, low-density lipoprotein particle binding, low-density lipoprotein receptor activity, protein binding
ECE1_MOUSE	Endothelin-converting enzyme 1	Metal ion binding, metalloendopeptidase activity, metallopeptidase activity, protein homodimerization activity
STX12_MOUSE	Syntaxin-12	SNAP receptor activity, SNARE binding, protein binding
TOM1_MOUSE	Target of Myb protein 1	Clathrin binding
CO3A1_MOUSE	Collagen alpha-1(III) chain	SMAD binding, extracellular matrix structural constituent, integrin binding, metal ion binding, platelet-derived growth factor binding
NPC2_MOUSE	Epididymal secretory protein E1	Cholesterol binding, enzyme binding
HA1B_MOUSE	H-2 class I histocompatibility antigen, K-B alpha chain	RNA binding, beta-2-microglobulin binding, peptide antigen binding, receptor binding
VKOR1_MOUSE	Vitamin K epoxide reductase complex subunit 1	Oxidoreductase activity, quinone binding, vitamin-K-epoxide reductase (warfarin-sensitive) activity
CUL3_MOUSE	Cullin-3	POZ domain binding, cyclin binding, protein binding, protein heterodimerization activity, protein homodimerization activity, ubiquitin protein ligase activity, ubiquitin protein ligase binding
HA11_MOUSE	H-2 class I histocompatibility antigen, D-B alpha chain	RNA binding, beta-2-microglobulin binding, peptide antigen binding, protein binding
ACAD9_MOUSE	Acyl-CoA dehydrogenase family member 9, mitochondrial	Acyl-CoA dehydrogenase activity, electron carrier activity, fatty-acyl-CoA binding, flavin adenine dinucleotide binding, oxidoreductase activity, acting on the CH-CH group of donors, with a flavin as acceptor
TGBR3_MOUSE	Transforming growth factor beta receptor type 3	PDZ domain binding, SMAD binding, coreceptor activity, glycosaminoglycan binding, protein binding, transforming growth factor beta binding, transforming growth factor beta receptor activity, type III, transforming growth factor beta-activated receptor activity, type II transforming growth factor beta receptor binding
A4_MOUSE	Amyloid-beta A4 protein	DNA binding, PTB domain binding, enzyme binding, growth factor receptor binding, heparin binding, identical protein binding, peptidase activator activity, peptidase inhibitor activity, serine-type endopeptidase inhibitor activity, transition metal ion binding
RBMS2_MOUSE	RNA-binding motif, single-stranded-interacting protein 2	RNA binding, nucleotide binding
PAPS2_MOUSE	Bifunctional 3′-phosphoadenosine 5′-phosphosulfate synthase 2	ATP binding, adenylylsulfate kinase activity, catalytic activity, nucleotide binding, nucleotidyltransferase activity, sulfate adenylyltransferase (ATP) activity
SPA3N_MOUSE	Serine protease inhibitor A3N	Peptidase inhibitor activity, serine-type endopeptidase inhibitor activity
DDX17_MOUSE	Probable ATP-dependent RNA helicase DDX17	ATP binding, ATP-dependent RNA helicase activity, RNA binding, estrogen receptor binding, nucleotide binding, transcription coactivator activity
RIPK3_MOUSE	Receptor-interacting serine/threonine-protein kinase 3	ATP binding, NF-kappaB-inducing kinase activity, identical protein binding, nucleotide binding, protein complex binding
MCAT_MOUSE	Mitochondrial carnitine/acylcarnitine carrier protein	Acyl carnitine transmembrane transporter activity
GDN_MOUSE	Glia-derived nexin	Glycosaminoglycan binding, heparin binding, receptor binding, serine-type endopeptidase inhibitor activity
ERLN2_MOUSE	Erlin-2	Cholesterol binding, protein binding, ubiquitin protein ligase binding
ARPC5_MOUSE	Actin-related protein 2/3 complex subunit 5	Actin filament binding, structural constituent of cytoskeleton
AAAT_MOUSE	Neutral amino acid transporter B(0)	Neutral amino acid transmembrane transporter activity, symporter activity
NAGK_MOUSE	N-Acetyl-D-glucosamine kinase	ATP binding, N-acetylglucosamine kinase activity, N-acylmannosamine kinase activity, kinase activity, nucleotide binding
STAT1_MOUSE	Signal transducer and activator of transcription 1	DNA binding, RNA polymerase II core promoter proximal region sequence-specific DNA binding, RNA polymerase II core promoter sequence-specific DNA binding, cadherin binding involved in cell-cell adhesion, double-stranded DNA binding, enzyme binding, nuclear hormone receptor binding, protein homodimerization activity, signal transducer activity, transcription factor activity, RNA polymerase II core promoter sequence-specific, transcription factor activity, sequence-specific DNA binding, tumor necrosis factor receptor binding
CCD47_MOUSE	Coiled-coil domain-containing protein 47	RNA binding, calcium ion binding
NDUB7_MOUSE	NADH dehydrogenase [ubiquinone] 1 beta subcomplex subunit 7	NADH dehydrogenase (ubiquinone) activity
F13A_MOUSE	Coagulation factor XIII A chain	Metal ion binding, protein-glutamine gamma-glutamyltransferase activity
SUCA_MOUSE	Succinate–CoA ligase [ADP/GDP-forming] subunit alpha, mitochondrial	GTP binding, RNA binding, cofactor binding, ligase activity, nucleotide binding, succinate-CoA ligase (ADP-forming) activity, succinate-CoA ligase (GDP-forming) activity
FACR1_MOUSE	Fatty acyl-CoA reductase 1	Fatty-acyl-CoA reductase (alcohol-forming) activity, long-chain-fatty-acyl-CoA reductase activity, oxidoreductase activity, acting on the aldehyde or oxo group of donors, NAD or NADP as acceptor
EFTS_MOUSE	Elongation factor Ts, mitochondrial	RNA binding, translation elongation factor activity
TM165_MOUSE	Transmembrane protein 165	Molecular function
ADA15_MOUSE	Disintegrin and metalloproteinase domain-containing protein 15	SH3 domain binding, integrin binding, metal ion binding, metalloendopeptidase activity, metallopeptidase activity, peptidase activity, protein binding
SNP23_MOUSE	Synaptosomal-associated protein 23	SNAP receptor activity, syntaxin binding
PRIO_MOUSE	Major prion protein	ATP-dependent protein binding, chaperone binding, copper ion binding, identical protein binding, ion channel binding, lamin binding, microtubule binding
LY6A_MOUSE	Lymphocyte antigen 6A-2/6E-1	
PLP2_MOUSE	Proteolipid protein 2	Chemokine binding
TSNAX_MOUSE	Translin-associated protein X	A2A adenosine receptor binding, RNA binding, metal ion binding, protein complex binding, sequence-specific DNA binding, single-stranded DNA binding
T106A_MOUSE	Transmembrane protein 106A	Molecular function
UBFD1_MOUSE	Ubiquitin domain-containing protein UBFD1	RNA binding, cadherin binding involved in cell-cell adhesion
PLEK_MOUSE	Pleckstrin	Phosphatidylinositol-3,4-bisphosphate binding, protein homodimerization activity, protein kinase C binding
TPBG_MOUSE	Trophoblast glycoprotein	
ILEUA_MOUSE	Leukocyte elastase inhibitor A	Serine-type endopeptidase inhibitor activity
TIMP2_MOUSE	Metalloproteinase inhibitor 2	Enzyme activator activity, integrin binding, metal ion binding, metalloendopeptidase inhibitor activity, peptidase inhibitor activity, protease binding
FMR1_MOUSE	Synaptic functional regulator FMR1	G-quadruplex RNA binding, RNA stem-loop binding, RNA strand annealing activity, chromatin binding, dynein complex binding, ion channel binding, mRNA 3′-UTR binding, mRNA 5′-UTR binding, methylated histone binding, miRNA binding, microtubule binding, poly(G) binding, poly(U) RNA binding, protein heterodimerization activity, protein homodimerization activity, ribosome binding, sequence-specific mRNA binding, siRNA binding, translation initiation factor binding, translation repressor activity
DCUP_MOUSE	Uroporphyrinogen decarboxylase	Carboxy-lyase activity, uroporphyrinogen decarboxylase activity
PLIN4_MOUSE	Perilipin-4	
RHEB_MOUSE	GTP-binding protein Rheb	GTP binding, metal ion binding, nucleotide binding, protein kinase binding
TPD52_MOUSE	Tumor protein D52	Calcium ion binding, protein binding, protein heterodimerization activity, protein homodimerization activity
LDLR_MOUSE	Low-density lipoprotein receptor	Calcium ion binding, glycoprotein binding, identical protein binding, low-density lipoprotein particle binding, low-density lipoprotein receptor activity, protease binding, very-low-density lipoprotein particle receptor activity
PDC10_MOUSE	Programmed cell death protein 10	Protein N-terminus binding, protein homodimerization activity, protein kinase binding
UFM1_MOUSE	Ubiquitin-fold modifier 1	Molecular function
SRXN1_MOUSE	Sulfiredoxin-1	ATP binding, antioxidant activity, nucleotide binding, oxidoreductase activity, oxidoreductase activity, acting on a sulfur group of donors, sulfiredoxin activity
GAPR1_MOUSE	Golgi-associated plant pathogenesis-related protein 1	Protein homodimerization activity
CSN5_MOUSE	COP9 signalosome complex subunit 5	Hydrolase activity, metal ion binding, metallopeptidase activity, protein binding, thiol-dependent ubiquitin-specific protease activity, transcription coactivator activity
OCAD1_MOUSE	OCIA domain-containing protein 1	Molecular function
DCAKD_MOUSE	Dephospho-CoA kinase domain-containing protein	ATP binding, dephospho-CoA kinase activity, nucleotide binding
CD166_MOUSE	CD166 antigen	Protein binding
ATG3_MOUSE	Ubiquitin-like-conjugating enzyme ATG3	Atg12 transferase activity, Atg8 ligase activity, enzyme binding, ligase activity, ubiquitin-like protein transferase activity
DAG1_MOUSE	Dystroglycan	SH2 domain binding, actin binding, alpha-actinin binding, calcium ion binding, dystroglycan binding, protein binding, protein complex binding, structural constituent of muscle, tubulin binding, vinculin binding
RAP2A_MOUSE	Ras-related protein Rap-2a	GTP binding, GTPase activity, nucleotide binding, protein binding
MPEG1_MOUSE	Macrophage-expressed gene 1 protein	
RBX1_MOUSE	E3 ubiquitin-protein ligase RBX1	NEDD8 transferase activity, cullin family protein binding, eukaryotic initiation factor 4E binding, ligase activity, protein complex binding, ubiquitin protein ligase activity, ubiquitin protein ligase binding, ubiquitin-ubiquitin ligase activity, zinc ion binding	
VPS16_MOUSE	Vacuolar protein sorting-associated protein 16 homolog	Actin binding	
PKHO2_MOUSE	Pleckstrin homology domain-containing family O member 2	Molecular function	
GAS6_MOUSE	Growth arrest-specific protein 6	Binding, bridging, calcium ion binding, cysteine-type endopeptidase inhibitor activity involved in apoptotic process, phosphatidylserine binding, protein tyrosine kinase activator activity, receptor agonist activity, receptor binding, receptor tyrosine kinase binding, voltage-gated calcium channel activity	
GMPPA_MOUSE	Mannose-1-phosphate guanyltransferase alpha	Nucleotidyltransferase activity, transferase activity	
COMD4_MOUSE	COMM domain-containing protein 4	Molecular function	
MECR_MOUSE	Enoyl-[acyl-carrier-protein] reductase, mitochondrial	Ligand-dependent nuclear receptor binding, oxidoreductase activity, trans-2-enoyl-CoA reductase (NADPH) activity, zinc ion binding	
PTMS_MOUSE	Parathymosin	Zinc ion binding	
RMD3_MOUSE	Regulator of microtubule dynamics protein 3	Molecular function	
SRSF1_MOUSE	Serine/arginine-rich splicing factor 1	RNA binding, RS domain binding, mRNA binding, nucleotide binding, protein kinase B binding	
TMCO1_MOUSE	Calcium load-activated calcium channel	Calcium channel activity	
TMX1_MOUSE	Thioredoxin-related transmembrane protein 1	Disulfide oxidoreductase activity, protein disulfide isomerase activity	
VATG1_MOUSE	V-type proton ATPase subunit G 1	ATPase activity, ATPase binding, hydrogen-exporting ATPase activity, phosphorylative mechanism, hydrolase activity, acting on acid anhydrides, catalyzing transmembrane movement of substances	
STMN1_MOUSE	Stathmin	Protein binding, tubulin binding	
H14_MOUSE	Histone H1.4	DNA binding, RNA binding, chromatin DNA binding, protein binding	
PROS_MOUSE	Vitamin K-dependent protein S	Calcium ion binding	
PP2BA_MOUSE	Serine/threonine-protein phosphatase 2B catalytic subunit alpha isoform	Calcium-dependent protein serine/threonine phosphatase activity, calmodulin binding, calmodulin-dependent protein phosphatase activity, drug binding, enzyme binding, hydrolase activity, metal ion binding, protein dimerization activity, protein heterodimerization activity	
VAPB_MOUSE	Vesicle-associated membrane protein-associated protein B	FFAT motif binding, beta-tubulin binding, cadherin binding involved in cell-cell adhesion, enzyme binding, microtubule binding, protein heterodimerization activity, protein homodimerization activity	
TBL1R_MOUSE	F-box-like/WD repeat-containing protein TBL1XR1	DNA binding, beta-catenin binding, histone binding, protein N-terminus binding, protein binding, transcription corepressor activity, transcription regulatory region DNA binding	
RAB35_MOUSE	Ras-related protein Rab-35	GDP binding, GTP binding, GTPase activity, nucleotide binding, phosphatidylinositol-4,5-bisphosphate binding, protein binding	
BID_MOUSE	BH3-interacting domain death agonist	Protein heterodimerization activity, ubiquitin protein ligase binding	
GLTP_MOUSE	Glycolipid transfer protein	Glycolipid binding, glycolipid transporter activity	
IL1RA_MOUSE	Interleukin-1 receptor antagonist protein	Cytokine activity, interleukin-1 Type I receptor antagonist activity, interleukin-1 Type II receptor antagonist activity, interleukin-1, Type I receptor binding, interleukin-1, Type II receptor binding	
PDXK_MOUSE	Pyridoxal kinase	ATP binding, kinase activity, lithium ion binding, magnesium ion binding, nucleotide binding, potassium ion binding, protein homodimerization activity, pyridoxal kinase activity, pyridoxal phosphate binding, sodium ion binding, zinc ion binding	
TRI25_MOUSE	E3 ubiquitin/ISG15 ligase TRIM25	RNA binding, acid-amino acid ligase activity, cadherin binding involved in cell-cell adhesion, ligase activity, metal ion binding, ubiquitin protein ligase activity involved in ERAD pathway, ubiquitin-protein transferase activity, zinc ion binding	
VATD_MOUSE	V-type proton ATPase subunit D	ATPase activity, coupled to transmembrane movement of substances	
IL1AP_MOUSE	Interleukin-1 receptor accessory protein	Interleukin-1 receptor activity, interleukin-33 receptor activity, protein tyrosine kinase binding, signal transducer activity	
SFXN1_MOUSE	Sideroflexin-1	Ion transmembrane transporter activity	
EEA1_MOUSE	Early endosome antigen 1	1-Phosphatidylinositol binding, GTP-dependent protein binding, metal ion binding, protein homodimerization activity	
CBR2_MOUSE	Carbonyl reductase [NADPH] 2	Carbonyl reductase (NADPH) activity, oxidoreductase activity, protein binding, protein self-association	
FKB14_MOUSE	Peptidyl-prolyl cis-trans isomerase FKBP14	FK506 binding, calcium ion binding, isomerase activity, peptidyl-prolyl cis-trans isomerase activity	
DEGS1_MOUSE	Sphingolipid delta(4)-desaturase DES1	Sphingolipid delta-4 desaturase activity	
FCGRN_MOUSE	IgG receptor FcRn large subunit p51	IgG binding, IgG receptor activity, antigen binding, beta-2-microglobulin binding, peptide antigen binding	
ACSF2_MOUSE	Acyl-CoA synthetase family member 2, mitochondrial	ATP binding, ligase activity, molecular function, nucleotide binding	
ADA_MOUSE	Adenosine deaminase	Adenosine deaminase activity, deaminase activity, hydrolase activity, purine nucleoside binding, zinc ion binding	
ASC_MOUSE	Apoptosis-associated speck-like protein containing a CARD	BMP receptor binding, Pyrin domain binding, cysteine-type endopeptidase activity involved in apoptotic process, enzyme binding, interleukin-6 receptor binding, ion channel binding, myosin I binding, peptidase activator activity involved in apoptotic process, protease binding, protein dimerization activity, protein homodimerization activity, tropomyosin binding	
TXD17_MOUSE	Thioredoxin domain-containing protein 17	Peroxidase activity, protein-disulfide reductase activity	
MXRA8_MOUSE	Matrix remodeling-associated protein 8	Molecular function	
COX6C_MOUSE	Cytochrome c oxidase subunit 6C	Cytochrome c oxidase activity, molecular function	
COASY_MOUSE	Bifunctional coenzyme A synthase	ATP binding, dephospho-CoA kinase activity, nucleotide binding, pantetheine-phosphate adenylyltransferase activity, transferase activity	
CHCH2_MOUSE	Coiled-coil-helix-coiled-coil-helix domain-containing protein 2	Sequence-specific DNA binding, transcription factor binding	
SHOC2_MOUSE	Leucine-rich repeat protein SHOC-2	Protein phosphatase 1 binding, protein phosphatase binding	
CDV3_MOUSE	Protein CDV3	Molecular function	
ERAP1_MOUSE	Endoplasmic reticulum aminopeptidase 1	Endopeptidase activity, interleukin-6 receptor binding, metalloaminopeptidase activity, metalloexopeptidase activity, peptide binding, zinc ion binding	
AL3A2_MOUSE	Fatty aldehyde dehydrogenase	3-Chloroallyl aldehyde dehydrogenase activity, aldehyde dehydrogenase (NAD) activity, aldehyde dehydrogenase [NAD(P)+] activity, long-chain-alcohol oxidase activity	
ABHEB_MOUSE	Protein ABHD14B	Hydrolase activity	
GLYG_MOUSE	Glycogenin-1	Glycogenin glucosyltransferase activity, metal ion binding, transferase activity, transferase activity, transferring glycosyl groups	
BPNT1_MOUSE	3′(2′),5′-Bisphosphate nucleotidase 1	3′(2′),5′-Bisphosphate nucleotidase activity, hydrolase activity, magnesium ion binding, metal ion binding	
IFM2_MOUSE	Interferon-induced transmembrane protein 2	Molecular function	
PFD6_MOUSE	Prefoldin subunit 6	Chaperone binding, unfolded protein binding	
COMD8_MOUSE	COMM domain-containing protein 8	Molecular function	
S10AD_MOUSE	Protein S100-A13	RAGE receptor binding, calcium ion binding, copper ion binding, fibroblast growth factor binding, lipid binding, metal ion binding, protein homodimerization activity, zinc ion binding	
T176A_MOUSE	Transmembrane protein 176A	Protein binding	
SPD2B_MOUSE	SH3 and PX domain-containing protein 2B	SH2 domain binding, phosphatidylinositol-3,5-bisphosphate binding, phosphatidylinositol-3-phosphate binding, phosphatidylinositol-4-phosphate binding, phosphatidylinositol-5-phosphate binding, superoxide-generating NADPH oxidase activator activity	
RM46_MOUSE	39S ribosomal protein L46, mitochondrial	Hydrolase activity, structural constituent of ribosome	
ADAS_MOUSE	Alkyldihydroxyacetonephosphate synthase, peroxisomal	FAD binding, alkylglycerone-phosphate synthase activity, flavin adenine dinucleotide binding, oxidoreductase activity, acting on CH-OH group of donors	
APMAP_MOUSE	Adipocyte plasma membrane-associated protein	Arylesterase activity, hydrolase activity, acting on ester bonds, strictosidine synthase activity	
ARK72_MOUSE	Aflatoxin B1 aldehyde reductase member 2	Oxidoreductase activity, oxidoreductase activity, acting on the CH-OH group of donors, NAD or NADP as acceptor, phenanthrene-9,10-epoxide hydrolase activity	
BST2_MOUSE	Bone marrow stromal antigen 2	RNA binding, metalloendopeptidase inhibitor activity, protein homodimerization activity, signal transducer activity	
CASP1_MOUSE	Caspase-1	Cysteine-type endopeptidase activity, cysteine-type endopeptidase activity involved in apoptotic process, cysteine-type peptidase activity, protein binding	
CD180_MOUSE	CD180 antigen	Receptor activity	
CK054_MOUSE	Ester hydrolase C11orf54 homolog	Hydrolase activity, acting on ester bonds, metal ion binding, zinc ion binding	
COX7C_MOUSE	Cytochrome c oxidase subunit 7C, mitochondrial	Cytochrome c oxidase activity	
CPSF7_MOUSE	Cleavage and polyadenylation specificity factor subunit 7	RNA binding, nucleotide binding	
DCAF8_MOUSE	DDB1- and CUL4-associated factor 8	Molecular function	
DHB11_MOUSE	Estradiol 17-beta-dehydrogenase 11	Estradiol 17-beta-dehydrogenase activity, steroid dehydrogenase activity	
ERGI1_MOUSE	Endoplasmic reticulum-Golgi intermediate compartment protein 1	Molecular function	
ERGI3_MOUSE	Endoplasmic reticulum-Golgi intermediate compartment protein 3	Molecular function	
FKB15_MOUSE	FK506-binding protein 15	Actin binding, peptidyl-prolyl cis-trans isomerase activity	
GSTA1_MOUSE	Glutathione S-transferase A1	Glutathione transferase activity, transferase activity	
IL4RA_MOUSE	Interleukin-4 receptor subunit alpha	Cytokine receptor activity, protein binding	
ILF2_MOUSE	Interleukin enhancer-binding factor 2	ATP binding, DNA binding, RNA binding, double-stranded RNA binding, transferase activity	
ITAM_MOUSE	Integrin alpha-M	Glycoprotein binding, heparan sulfate proteoglycan binding, heparin binding, metal ion binding, opsonin binding	
LSG1_MOUSE	Large subunit GTPase 1 homolog	GTP binding, GTPase activity, nucleotide binding	
NDUA2_MOUSE	NADH dehydrogenase [ubiquinone] 1 alpha subcomplex subunit 2	NADH dehydrogenase (ubiquinone) activity	
NINJ1_MOUSE	Ninjurin-1		
OSMR_MOUSE	Oncostatin-M-specific receptor subunit beta	Cytokine binding, cytokine receptor activity, growth factor binding, oncostatin-M receptor activity	
P2RX4_MOUSE	P2X purinoceptor 4	ATP binding, cadherin binding, extracellular ATP-gated cation channel activity, ion channel activity, purinergic nucleotide receptor activity	
PFD1_MOUSE	Prefoldin subunit 1	Protein binding involved in protein folding, unfolded protein binding	
PGFS_MOUSE	Prostamide/prostaglandin F synthase	Prostaglandin-F synthase activity, thioredoxin peroxidase activity	
PR2C2_MOUSE	Prolactin-2C2	Growth factor activity, hormone activity	
PRRX1_MOUSE	Paired mesoderm homeobox protein 1	DNA binding, HMG box domain binding, RNA polymerase II transcription coactivator activity, sequence-specific DNA binding	
RL35_MOUSE	60S ribosomal protein L35	RNA binding, mRNA binding, structural constituent of ribosome	
S10A1_MOUSE	Protein S100-A1	ATPase binding, S100 protein binding, calcium ion binding, protein homodimerization activity	
SBDS_MOUSE	Ribosome maturation protein SBDS	Microtubule binding, rRNA binding, ribosome binding	
SDF2_MOUSE	Stromal cell-derived factor 2	Dolichyl-phosphate-mannose-protein mannosyltransferase activity	
SERC1_MOUSE	Serine incorporator 1	L-Serine transmembrane transporter activity	
TIAR_MOUSE	Nucleolysin TIAR	AU-rich element binding, RNA binding, nucleotide binding	
TRADD_MOUSE	Tumor necrosis factor receptor type 1-associated DEATH domain protein	Binding, bridging, death domain binding, identical protein binding, kinase binding, protein complex binding, signal transducer activity, tumor necrosis factor receptor binding	
TRNT1_MOUSE	CCA tRNA nucleotidyltransferase 1, mitochondrial	ATP binding, ATP:3′-cytidine-cytidine-tRNA adenylyltransferase activity, CTP:3′-cytidine-tRNA cytidylyltransferase activity, CTP:tRNA cytidylyltransferase activity, nucleotide binding, tRNA binding, tRNA nucleotidyltransferase activity	
VPS52_MOUSE	Vacuolar protein sorting-associated protein 52 homolog	Syntaxin binding	
ZFYV1_MOUSE	Zinc finger FYVE domain-containing protein 1	1-Phosphatidylinositol binding, metal ion binding, phosphatidylinositol-3,4,5-trisphosphate binding, phosphatidylinositol-3,4-bisphosphate binding	
ZWINT_MOUSE	ZW10 interactor	Protein N-terminus binding, protein binding	
FBLN3_MOUSE	EGF-containing fibulin-like extracellular matrix protein 1	Calcium ion binding, epidermal growth factor receptor binding, epidermal growth factor-activated receptor activity, growth factor activity	
NDUB9_MOUSE	NADH dehydrogenase [ubiquinone] 1 beta subcomplex subunit 9	NADH dehydrogenase (ubiquinone) activity	
MIC10_MOUSE	MICOS complex subunit Mic10	Molecular function	
NPL_MOUSE	N-Acetylneuraminate lyase	N-Acetylneuraminate lyase activity, lyase activity	
UCRI_MOUSE	Cytochrome b-c1 complex subunit Rieske, mitochondrial	2 iron, 2 sulfur cluster binding, metal ion binding, oxidoreductase activity, acting on diphenols and related substances as donors, protein complex binding, ubiquinol-cytochrome c reductase activity	
CD97_MOUSE	CD97 antigen	G-protein coupled receptor activity, calcium ion binding, signal transducer activity	
WDR26_MOUSE	WD repeat-containing protein 26	Molecular function	
XIRP2_MOUSE	Xin actin-binding repeat-containing protein 2	Actin binding, alpha-actinin binding, protein binding, zinc ion binding	
DYR_MOUSE	Dihydrofolate reductase	NADP binding, NADPH binding, dihydrofolate reductase activity, dihydrofolic acid binding, folic acid binding, mRNA binding, methotrexate binding, oxidoreductase activity	
AIMP1_MOUSE	Aminoacyl tRNA synthase complex-interacting multifunctional protein 1	GTPase binding, cytokine activity, protein homodimerization activity, tRNA binding	
SYTC2_MOUSE	Probable threonine–tRNA ligase 2, cytoplasmic	ATP binding, RNA binding, nucleotide binding, threonine-tRNA ligase activity	
RAB4B_MOUSE	Ras-related protein Rab-4B	GTP binding, nucleotide binding	
RAB8A_MOUSE	Ras-related protein Rab-8A	GDP binding, GTP binding, GTPase activity, Rab GTPase binding, myosin V binding, nucleotide binding, protein binding, protein kinase binding	
AT2B1_MOUSE	Plasma membrane calcium-transporting ATPase 1	ATP binding, PDZ domain binding, calcium-transporting ATPase activity, calmodulin binding, hydrolase activity, metal ion binding, nucleotide binding	
GNA11_MOUSE	Guanine nucleotide-binding protein subunit alpha-11	G-protein beta/gamma-subunit complex binding, GTP binding, GTPase activity, alkylglycerophosphoethanolamine phosphodiesterase activity, guanyl nucleotide binding, metal ion binding, signal transducer activity, type 2A serotonin receptor binding	
SUCB2_MOUSE	Succinate–CoA ligase [GDP-forming] subunit beta, mitochondrial	ATP binding, GDP binding, GTP binding, ligase activity, metal ion binding, nucleotide binding, protein heterodimerization activity, succinate-CoA ligase (GDP-forming) activity, succinate-semialdehyde dehydrogenase (NAD+) activity	
GIPC1_MOUSE	PDZ domain-containing protein GIPC1	GTPase activator activity, PDZ domain binding, actin binding, cadherin binding involved in cell-cell adhesion, myosin binding, protein homodimerization activity, receptor binding	
ACPM_MOUSE	Acyl carrier protein, mitochondrial	Molecular function	
ALG11_MOUSE	GDP-Man:Man(3)GlcNAc(2)-PP-Dol alpha-1,2-mannosyltransferase	GDP-Man:Man3GlcNAc2-PP-Dol alpha-1,2-mannosyltransferase activity, transferase activity, transferase activity, transferring glycosyl groups	
AN32E_MOUSE	Acidic leucine-rich nuclear phosphoprotein 32 family member E	Histone binding, phosphatase inhibitor activity	
APOE_MOUSE	Apolipoprotein E	Antioxidant activity, beta-amyloid binding, cholesterol binding, cholesterol transporter activity, heparin binding, lipid transporter activity, lipoprotein particle binding, low-density lipoprotein particle receptor binding, metal chelating activity, phosphatidylcholine-sterol O-acyltransferase activator activity, phospholipid binding, protein binding, protein homodimerization activity, tau protein binding, very-low-density lipoprotein particle receptor binding	
ARMC6_MOUSE	Armadillo repeat-containing protein 6	Molecular function	
BAP31_MOUSE	B cell receptor-associated protein 31	MHC class I protein binding, protein complex binding	
CC90B_MOUSE	Coiled-coil domain-containing protein 90B, mitochondrial	Molecular function	
CCD22_MOUSE	Coiled-coil domain-containing protein 22	Cullin family protein binding, protein binding	
CDIPT_MOUSE	CDP-diacylglycerol–inositol 3-phosphatidyltransferase	CDP-diacylglycerol-inositol 3-phosphatidyltransferase activity, alcohol binding, carbohydrate binding, diacylglycerol binding, manganese ion binding	
CGAT1_MOUSE	Chondroitin sulfate N-acetylgalactosaminyltransferase 1	Glucuronosyl-N-acetylgalactosaminyl-proteoglycan 4-beta-N-acetylgalactosaminyltransferase activity, glucuronosyltransferase activity, glucuronylgalactosylproteoglycan 4-beta-N-acetylgalactosaminyltransferase activity, metal ion binding, peptidoglycan glycosyltransferase activity	
CN37_MOUSE	2′,3′-Cyclic-nucleotide 3′-phosphodiesterase	2′,3′-Cyclic-nucleotide 3′-phosphodiesterase activity, RNA binding, cyclic nucleotide binding, hydrolase activity	
CO4A2_MOUSE	Collagen alpha-2(IV) chain	Extracellular matrix structural constituent	
CPSF6_MOUSE	Cleavage and polyadenylation specificity factor subunit 6	RNA binding, mRNA binding, nucleotide binding	
CRK_MOUSE	Adapter molecule crk	SH2 domain binding, SH3/SH2 adaptor activity, enzyme binding, ephrin receptor binding, protein binding, bridging, protein phosphorylated amino acid binding	
CTBP2_MOUSE	C-terminal-binding protein 2	NAD binding, chromatin binding, oxidoreductase activity, oxidoreductase activity, acting on the CH-OH group of donors, NAD or NADP as acceptor, protein homodimerization activity, retinoic acid receptor binding, transcription coactivator activity, transcription corepressor activity	
DAD1_MOUSE	Dolichyl-diphosphooligosaccharide–protein glycosyltransferase subunit DAD1	Dolichyl-diphosphooligosaccharide-protein glycotransferase activity, transferase activity, transferring glycosyl groups	
DERL2_MOUSE	Derlin-2	Molecular function	
DHSO_MOUSE	Sorbitol dehydrogenase	D-Xylulose reductase activity, L-iditol 2-dehydrogenase activity, NAD binding, identical protein binding, zinc ion binding	
DNJB4_MOUSE	DnaJ homolog subfamily B member 4	Chaperone binding, unfolded protein binding	
DYLT1_MOUSE	Dynein light chain Tctex-type 1	G-protein beta-subunit binding, GTP-dependent protein binding, identical protein binding, motor activity	
DYLT3_MOUSE	Dynein light chain Tctex-type 3	Identical protein binding, motor activity	
EFL1_MOUSE	Elongation factor-like GTPase 1	GTP binding, GTPase activity, nucleotide binding, ribosome binding	
EH1L1_MOUSE	EH domain-binding protein 1-like protein 1	Molecular function	
EMC8_MOUSE	ER membrane protein complex subunit 8	Molecular function	
ETHE1_MOUSE	Persulfide dioxygenase ETHE1, mitochondrial	Dioxygenase activity, iron ion binding, metal ion binding, sulfur dioxygenase activity	
FKB11_MOUSE	Peptidyl-prolyl cis-trans isomerase FKBP11	FK506 binding, isomerase activity, peptidyl-prolyl cis-trans isomerase activity	
FNTB_MOUSE	Protein farnesyltransferase subunit beta	Drug binding, farnesyltranstransferase activity, isoprenoid binding, peptide binding, protein farnesyltransferase activity, zinc ion binding	
FUND2_MOUSE	FUN14 domain-containing protein 2	Molecular function	
GGA1_MOUSE	ADP-ribosylation factor-binding protein GGA1		
GGT5_MOUSE	Glutathione hydrolase 5 proenzyme	Gamma-glutamyltransferase activity, glutathione hydrolase activity	
GINM1_MOUSE	Glycoprotein integral membrane protein 1	Molecular function	
GLRX5_MOUSE	Glutaredoxin-related protein 5, mitochondrial	2 iron, 2 sulfur cluster binding, electron carrier activity, metal ion binding, protein disulfide oxidoreductase activity	
HDAC7_MOUSE	Histone deacetylase 7	14-3-3 protein binding, NAD-dependent histone deacetylase activity (H3-K14 specific), activating transcription factor binding, chromatin binding, metal ion binding, protein kinase C binding, protein kinase binding, repressing transcription factor binding, transcription corepressor activity	
HEBP1_MOUSE	Heme-binding protein 1	Heme binding	
HIG1A_MOUSE	HIG1 domain family member 1A, mitochondrial	Molecular function	
HOME3_MOUSE	Homer protein homolog 3	G-protein coupled glutamate receptor binding, protein C-terminus binding, protein domain specific binding	
HRG1_MOUSE	Heme transporter HRG1	Heme transporter activity, molecular function	
HS2ST_MOUSE	Heparan sulfate 2-O-sulfotransferase 1	Heparan sulfate 2-O-sulfotransferase activity, transferase activity	
IC1_MOUSE	Plasma protease C1 inhibitor	Peptidase inhibitor activity, serine-type endopeptidase inhibitor activity	
IDHG1_MOUSE	Isocitrate dehydrogenase [NAD] subunit gamma 1, mitochondrial	ATP binding, NAD binding, isocitrate dehydrogenase (NAD+) activity, magnesium ion binding, oxidoreductase activity	
LRN4L_MOUSE	LRRN4 C-terminal-like protein	Molecular function	
LTOR5_MOUSE	Ragulator complex protein LAMTOR5	Guanyl-nucleotide exchange factor activity, protein complex scaffold	
LXN_MOUSE	Latexin	Enzyme inhibitor activity, heparin binding, metalloendopeptidase inhibitor activity	
MA2B2_MOUSE	Epididymis-specific alpha-mannosidase	Alpha-mannosidase activity, carbohydrate binding, hydrolase activity, mannosidase activity, zinc ion binding	
MDR1A_MOUSE	Multidrug resistance protein 1A	ATP binding, ATPase activity, coupled, ATPase activity, coupled to transmembrane movement of substances, ceramide-translocating ATPase activity, nucleotide binding, phosphatidylcholine-translocating ATPase activity, phosphatidylethanolamine-translocating ATPase activity, xenobiotic-transporting ATPase activity	
MIC13_MOUSE	MICOS complex subunit MIC13	Molecular function	
MMAB_MOUSE	Cob(I)yrinic acid a,c-diamide adenosyltransferase, mitochondrial	ATP binding, cob(I)yrinic acid a,c-diamide adenosyltransferase activity, nucleotide binding, transferase activity	
MMSA_MOUSE	Methylmalonate-semialdehyde dehydrogenase [acylating], mitochondrial	RNA binding, aldehyde dehydrogenase (NAD) activity, malonate-semialdehyde dehydrogenase (acetylating) activity, methylmalonate-semialdehyde dehydrogenase (acylating) activity, oxidoreductase activity	
MPI_MOUSE	Mannose-6-phosphate isomerase	Isomerase activity, mannose-6-phosphate isomerase activity, zinc ion binding	
NAGA_MOUSE	N-Acetylglucosamine-6-phosphate deacetylase	N-Acetylglucosamine-6-phosphate deacetylase activity, hydrolase activity, metal ion binding	
NDUS4_MOUSE	NADH dehydrogenase [ubiquinone] iron-sulfur protein 4, mitochondrial	NADH dehydrogenase (ubiquinone) activity	
NDUS5_MOUSE	NADH dehydrogenase [ubiquinone] iron-sulfur protein 5	Molecular function	
NHLC3_MOUSE	NHL repeat-containing protein 3	Molecular function	
NU160_MOUSE	Nuclear pore complex protein Nup160	Nucleocytoplasmic transporter activity	
NUP50_MOUSE	Nuclear pore complex protein Nup50	Ran GTPase binding	
OAS1A_MOUSE	2′-5′-Oligoadenylate synthase 1A	2′-5′-Oligoadenylate synthetase activity, ATP binding, double-stranded RNA binding, metal ion binding, nucleotide binding, protein binding, transferase activity	
PEDF_MOUSE	Pigment epithelium-derived factor	Serine-type endopeptidase inhibitor activity	
PEX14_MOUSE	Peroxisomal membrane protein PEX14	Beta-tubulin binding, microtubule binding, protein N-terminus binding, receptor binding, transcription corepressor activity	
PEX19_MOUSE	Peroxisomal biogenesis factor 19	ATPase binding, peroxisome membrane class-1 targeting sequence binding, protein N-terminus binding, protein binding	
PIEZ1_MOUSE	Piezo-type mechanosensitive ion channel component 1	Cation channel activity, mechanically-gated ion channel activity	
PRAF1_MOUSE	Prenylated Rab acceptor protein 1	Identical protein binding, proline-rich region binding, protein C-terminus binding	
PRAF2_MOUSE	PRA1 family protein 2	Molecular function	
PREB_MOUSE	Prolactin regulatory element-binding protein	ARF guanyl-nucleotide exchange factor activity, DNA binding, GTPase binding, Rab guanyl-nucleotide exchange factor activity, Sar guanyl-nucleotide exchange factor activity, transcription factor activity, sequence-specific DNA binding	
PSN1_MOUSE	Presenilin-1	PDZ domain binding, aspartic-type endopeptidase activity, beta-catenin binding, cadherin binding, calcium channel activity, endopeptidase activity	
RAB31_MOUSE	Ras-related protein Rab-31	GDP binding, GTP binding, nucleotide binding	
RDH13_MOUSE	Retinol dehydrogenase 13	Oxidoreductase activity	
RT11_MOUSE	28S ribosomal protein S11, mitochondrial	mRNA 5′-UTR binding, small ribosomal subunit rRNA binding, structural constituent of ribosome	
S2546_MOUSE	Solute carrier family 25 member 46	Molecular function	
S35F6_MOUSE	Solute carrier family 35 member F6	Molecular function	
S39AE_MOUSE	Zinc transporter ZIP14	Ferrous iron transmembrane transporter activity, metal ion transmembrane transporter activity, zinc ion transmembrane transporter activity	
SCFD2_MOUSE	Sec1 family domain-containing protein 2	Molecular function	
SSRP1_MOUSE	FACT complex subunit SSRP1	DNA binding, RNA binding, chromatin binding	
STX2_MOUSE	Syntaxin-2	SNAP receptor activity, SNARE binding, calcium-dependent protein binding, protein dimerization activity	
SYVN1_MOUSE	E3 ubiquitin-protein ligase synoviolin	ATPase binding, chaperone binding, ligase activity, ubiquitin protein ligase activity, ubiquitin protein ligase activity involved in ERAD pathway, ubiquitin-specific protease binding, unfolded protein binding, zinc ion binding	
TENS3_MOUSE	Tensin-3	Molecular function	
TI8AB_MOUSE	Putative mitochondrial import inner membrane translocase subunit Tim8 A-B	Metal ion binding	
TMX2_MOUSE	Thioredoxin-related transmembrane protein 2	Molecular function	
TNR12_MOUSE	Tumor necrosis factor receptor superfamily member 12A	Protein binding	
TOIP2_MOUSE	Torsin-1A-interacting protein 2	ATPase activator activity, ATPase binding	
TOM5_MOUSE	Mitochondrial import receptor subunit TOM5 homolog	Protein transporter activity	
TPC13_MOUSE	Trafficking protein particle complex subunit 13	Molecular function	
UBP19_MOUSE	Ubiquitin carboxyl-terminal hydrolase 19	Hsp90 protein binding, Lys48-specific deubiquitinase activity, metal ion binding, thiol-dependent ubiquitin-specific protease activity, ubiquitin protein ligase binding	
UFSP2_MOUSE	Ufm1-specific protease 2	UFM1 hydrolase activity, cysteine-type peptidase activity, thiolester hydrolase activity	
VMP1_MOUSE	Vacuole membrane protein 1	Molecular function	
TIMP1_MOUSE	Metalloproteinase inhibitor 1	Cytokine activity, growth factor activity, metal ion binding, metalloendopeptidase inhibitor activity, protease binding	
TM9S4_MOUSE	Transmembrane 9 superfamily member 4	Molecular function	
PSB8_MOUSE	Proteasome subunit beta type-8	Endopeptidase activity, peptidase activity, threonine-type endopeptidase activity	
QCR7_MOUSE	Cytochrome b-c1 complex subunit 7	Ubiquinol-cytochrome c reductase activity	
ADA10_MOUSE	Disintegrin and metalloproteinase domain-containing protein 10	SH2 domain binding, SH3 domain binding, hydrolase activity, metal ion binding, metalloendopeptidase activity, metallopeptidase activity, protein binding, protein homodimerization activity, protein kinase binding	
KCT2_MOUSE	Keratinocyte-associated transmembrane protein 2	Molecular function	
NDRG2_MOUSE	Protein NDRG2		
YIF1B_MOUSE	Protein YIF1B	Molecular function	
TIM8B_MOUSE	Mitochondrial import inner membrane translocase subunit Tim8 B	Metal ion binding	
DJC24_MOUSE	DnaJ homolog subfamily C member 24	ATPase activator activity, ferrous iron binding, zinc ion binding	
VWA8_MOUSE	von Willebrand factor A domain-containing protein 8	ATP binding, ATPase activity, nucleotide binding	
FEN1_MOUSE	Flap endonuclease 1	5′-3′ exonuclease activity, 5′-flap endonuclease activity, DNA binding, RNA-DNA hybrid ribonuclease activity, catalytic activity, exonuclease activity, flap endonuclease activity, magnesium ion binding, manganese ion binding	
TPD54_MOUSE	Tumor protein D54	RNA binding	
MCL1_MOUSE	Induced myeloid leukemia cell differentiation protein Mcl-1 homolog	BH3 domain binding, protein heterodimerization activity, protein homodimerization activity	
PCBP4_MOUSE	Poly(rC)-binding protein 4	DNA binding, RNA binding, mRNA 3 ′-UTR binding	
GCR_MOUSE	Glucocorticoid receptor	RNA polymerase II core promoter proximal region sequence-specific DNA binding, glucocorticoid-activated RNA polymerase II transcription factor binding transcription factor activity, lipid binding, metal ion binding, protein complex binding, protein dimerization activity, sequence-specific DNA binding, steroid hormone binding, transcriptional activator activity, RNA polymerase II core promoter proximal region sequence-specific binding, zinc ion binding	
RT16_MOUSE	28S ribosomal protein S16, mitochondrial	Structural constituent of ribosome	
CD47_MOUSE	Leukocyte surface antigen CD47	Protein binding, thrombospondin receptor activity	
UBP24_MOUSE	Ubiquitin carboxyl-terminal hydrolase 24	Peptidase activity, thiol-dependent ubiquitinyl hydrolase activity	
ROMO1_MOUSE	Reactive oxygen species modulator 1	Molecular function	
GOT1B_MOUSE	Vesicle transport protein GOT1B	Signal transducer activity	
OLR1_MOUSE	Oxidized low-density lipoprotein receptor 1	Carbohydrate binding, low-density lipoprotein receptor activity	
SRSF2_MOUSE	Serine/arginine-rich splicing factor 2	RNA binding, nucleotide binding, pre-mRNA binding, protein kinase C binding	
COMD2_MOUSE	COMM domain-containing protein 2	Molecular function	
PYGL_MOUSE	Glycogen phosphorylase, liver form	AMP binding, ATP binding, bile acid binding, carbohydrate binding, catalytic activity, drug binding, glycogen phosphorylase activity, nucleotide binding, protein homodimerization activity, purine nucleobase binding, pyridoxal phosphate binding, vitamin binding	
NCF2_MOUSE	Neutrophil cytosol factor 2	Rac GTPase binding, protein C-terminus binding, superoxide-generating NADPH oxidase activity	
YIF1A_MOUSE	Protein YIF1A	Molecular function	
PEPL1_MOUSE	Probable aminopeptidase NPEPL1	Aminopeptidase activity, manganese ion binding, metalloexopeptidase activity	
WWOX_MOUSE	WW domain-containing oxidoreductase	RNA polymerase II transcription coactivator activity, enzyme binding, oxidoreductase activity	
PTH_MOUSE	Probable peptidyl-tRNA hydrolase	RNA binding, aminoacyl-tRNA hydrolase activity	
PYRD_MOUSE	Dihydroorotate dehydrogenase (quinone), mitochondrial	FMN binding, dihydroorotate dehydrogenase activity, drug binding, ubiquinone binding	
CTL1_MOUSE	Choline transporter-like protein 1	Choline transmembrane transporter activity	
LY6C1_MOUSE	Lymphocyte antigen 6C1		
OTU6B_MOUSE	OTU domain-containing protein 6B	Thiol-dependent ubiquitinyl hydrolase activity	
NECT2_MOUSE	Nectin-2	Cell adhesion molecule binding, identical protein binding, protein heterodimerization activity, protein homodimerization activity, receptor activity, receptor binding	
TTYH2_MOUSE	Protein tweety homolog 2	Chloride channel activity, molecular function	
LMOD1_MOUSE	Leiomodin-1	Actin binding, tropomyosin binding	
UD16_MOUSE	UDP-glucuronosyltransferase 1-6	Glucuronosyltransferase activity, protein heterodimerization activity, protein homodimerization activity, transferase activity, transferring hexosyl groups	
MYL6B_MOUSE	Myosin light chain 6B	Calcium ion binding, motor activity, structural constituent of muscle	

## Data Availability

The data used to support the findings of this study are available from the corresponding author upon request.

## Supplementary Materials

Supplementary Materials Supplementary Figure 1: a scatter plot of the housekeeping genes' quantitative values. A scatter plot showing the correlation (*R* ^2^ = 0.7964) between the quantitative value of the mouse primary cultured cells (P0) and cells passaged 3 times (P3): Atp5f1, B2m, Hprt1, Rplp1, Ppia, Rps18, Pgk1, Tfrc, Ywhaz, and Gapdh (*n* = 10). The dotted line is the regression line. Each dot shows the abbreviated name of the protein.
