# Supplementary figures and images for: A Liquid Chromatography with Tandem Mass Spectrometry-Based Proteomic Analysis of Primary Cultured Cells and Subcultured Cells Using Mouse Adipose-Derived Mesenchymal Stem Cells
Source: Stem Cells Int. 2019 Jan 10;2019:7274057. doi: 10.1155/2019/7274057 (PMC6362508; doi:10.1155/2019/7274057)

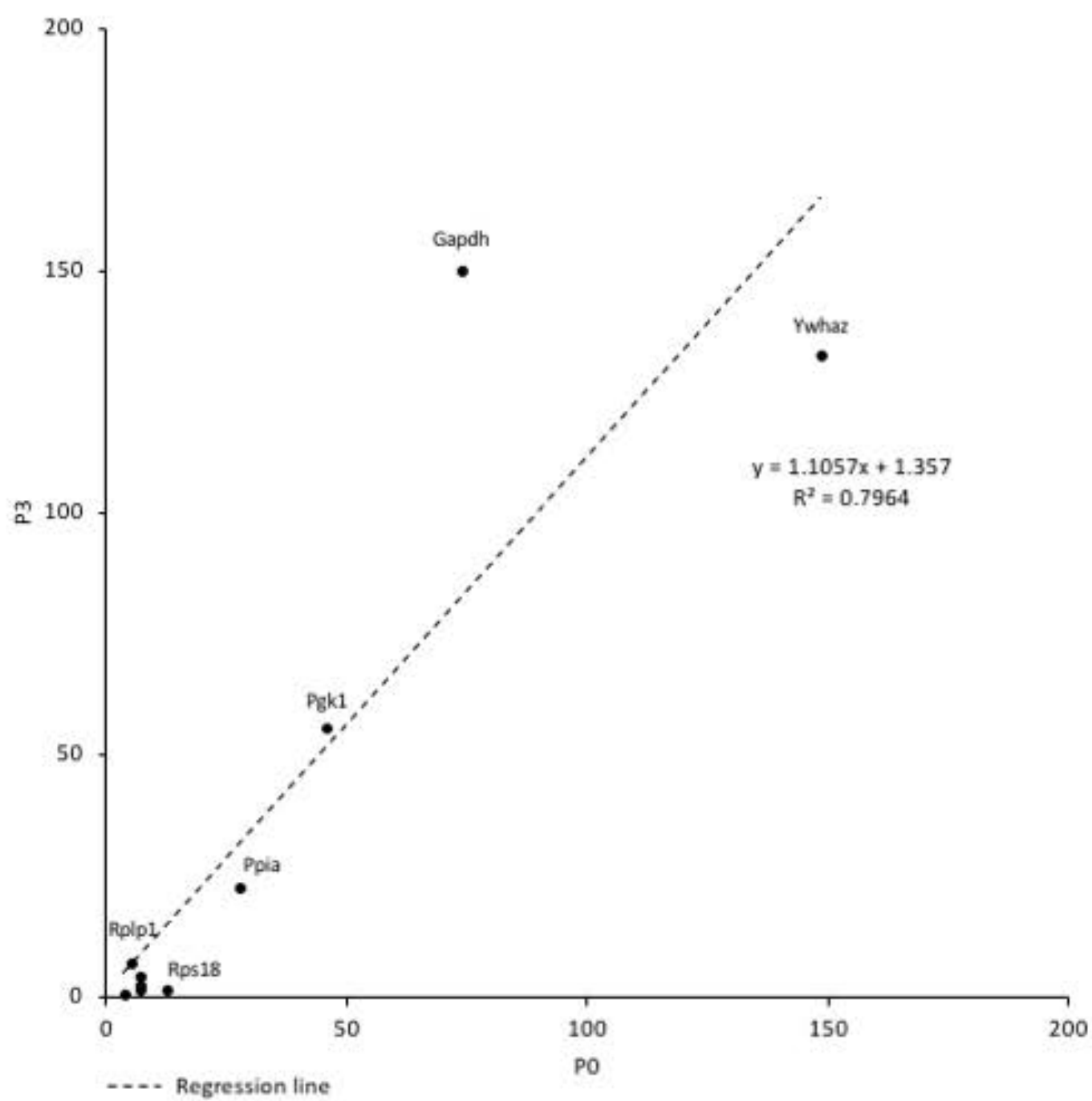

Supplement: Supplementary Materials — Supplementary Figure 1: a scatter plot of the housekeeping genes' quantitative values. A scatter plot showing the correlation (R 2 = 0.7964) between the quantitative value of the mouse primary cultured cells (P0) and cells passaged 3 times (P3): Atp5f1, B2m, Hprt1, Rplp1, Ppia, Rps18, Pgk1, Tfrc, Ywhaz, and Gapdh (n = 10). The dotted line is the regression line. Each dot shows the abbreviated name of the protein. [file 7274057.f1.pdf]
